# Short and Efficient Syntheses of Protoberberine Alkaloids using Palladium-Catalyzed Enolate Arylation**

**DOI:** 10.1002/anie.201409164

**Published:** 2014-10-27

**Authors:** Alice E Gatland, Ben S Pilgrim, Panayiotis A Procopiou, Timothy J Donohoe

**Affiliations:** Department of Chemistry, University of Oxford, Chemistry Research LaboratoryMansfield Road, Oxford, OX1 3TA (UK); GlaxoSmithKline Medicines Research Centre, Gunnels Wood Road, StevenageSG1 2NY (UK)

**Keywords:** alkaloids, natural products, palladium, synthetic methods, total synthesis

## Abstract

A concise synthesis of the biologically active alkaloid berberine is reported, and a versatile palladium-catalyzed enolate arylation is used to form the isoquinoline core. The overall yield of 50 % is a large improvement over the single, previous synthesis. By design, this modular route allows the rapid synthesis of other members of the protoberberine family (e.g., pseudocoptisine and palmatine) by substitution of the readily available aryl bromide and ketone coupling partners. Moreover, by combining enolate arylation with in situ functionalization, substituents can be rapidly and regioselectively introduced at the alkaloid C13 position, as demonstrated by the total synthesis of dehydrocorydaline. The avoidance of electrophilic aromatic substitution reactions to make the isoquinoline allows direct access to analogues possessing more varied electronic properties, such as the fluorine-containing derivative synthesized here.

The isoquinoline skeleton is one of the most prevalent core structures in alkaloid natural product chemistry. A subdivision of this class is the protoberberine alkaloids, an important group of secondary metabolites possessing significant biological activities as a result of their ability to bind or intercalate DNA.[1] Isolated from an extensive range of plants, all members feature a 5,6-dihydrodibenzo[*a*,*g*]quinolizinium moiety (the protoberberine skeleton), typically functionalized with hydroxy, methoxy, and methylenedioxy substituents. The parent compound, berberine (Scheme 1), is the most widely distributed and intensely studied of such alkaloids, thus having demonstrated antifungal, antibacterial, anti-inflammatory, antimalarial, antidiabetic, and anticancer activity.[1,2] Yet it has seen only one previous total synthesis, by Kametani and co-workers in 1969, which provided berberine iodide in low yield from a commercially unavailable starting material.[3] The majority of synthetic approaches to related compounds rely on electrophilic aromatic substitution to form the central rings. Whilst this allows access to analogues with electron-rich scaffolds, those with more varied electronic properties are not readily accessible.

**Scheme 1:**
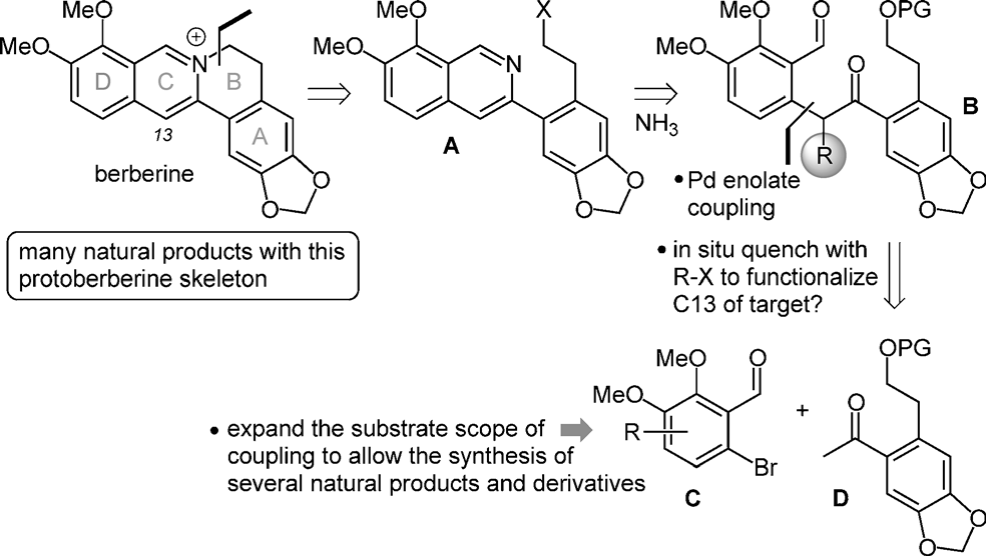
Retrosynthesis of berberine and skeletally related protoberberine natural products. PG=protecting group.

As we have previously reported, recent work in our laboratories has culminated in the development of a versatile method for the synthesis of isoquinolines.[4] This protocol employed the palladium-catalyzed α-arylation[5] of readily available ketones (using aryl bromides bearing an *ortho*-acetal) to generate masked 1,5-dicarbonyl intermediates, which could be cyclized with a source of NH_3_ to provide an extensive array of isoquinolines in excellent yields.[6] We therefore sought to apply this approach to the short total synthesis of berberine and related targets. Furthermore, an extension of our initial isoquinoline-forming methodology introduced functional groups at the C4 position of the isoquinoline skeleton.[7] This method exploited the fact that the product of enolate arylation is more acidic than the starting ketone and therefore exists as an enolate in the reaction mixture before quenching. Consequently, this enolate may be trapped in situ by a variety of alkyl, allyl, benzyl, and heteroatom electrophiles,[8] and these are carried through to occupy the C4 position in the resulting isoquinoline. It was anticipated that this highly effective methodology would be well suited to the introduction of substituents at C13 in the protoberberine alkaloids (Scheme 1). It was envisaged that subsequent formation of the B ring of berberine could be achieved through nucleophilic displacement of the leaving group X (anticipated to be chloride) by the isoquinoline nitrogen atom.[9] The isoquinoline **A** in question would be generated by aromatization of the dicarbonyl compound **B**, which is the product of a palladium-catalyzed coupling between the aryl bromide **C** and the enolate of ketone **D**. Trapping of the product enolate with an alkyl halide in situ would install a functional group (R) at C13.

The synthesis of berberine commenced with the preparation of the aryl bromide **2** by methylation and acetal protection of the commercially available benzaldehyde **1** in 98 % yield over two steps (Scheme 2). Its coupling partner, **4**, was accessed by BH_3_ reduction of the commercially available acid **3** and subsequent protection of the free alcohol as the pivaloate ester. Regioselective Friedel–Crafts acylation using Ac_2_O and ZnCl_2_ afforded **4** in 73 % yield over three steps.

**Scheme 2:**
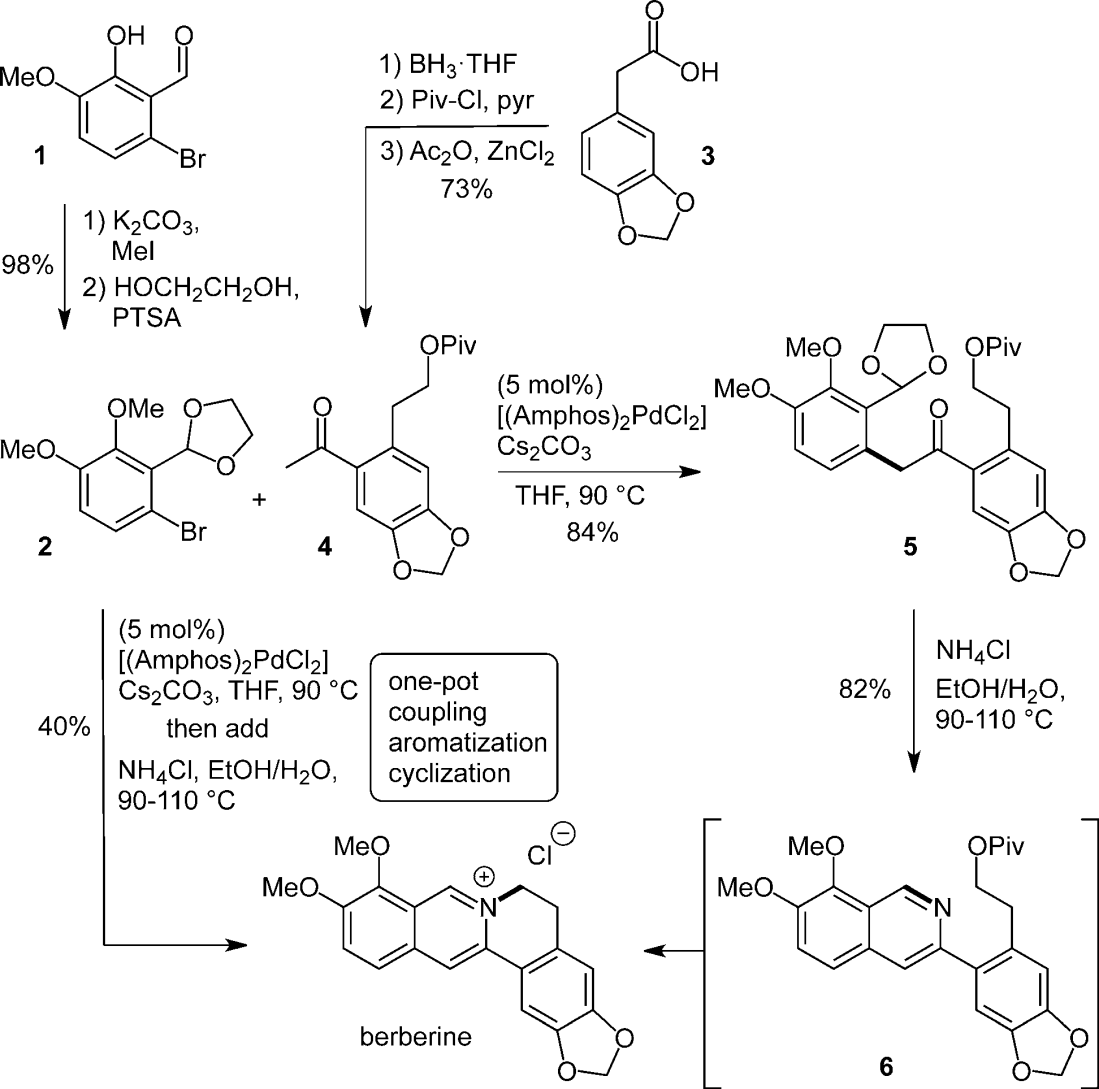
Synthesis of berberine by a sequential enolate coupling, aromatization, and cyclization sequence. Piv=pivaloyl, PTSA=*para*-toluenesulfonic acid, pyr=pyridine, THF=tetrahydrofuran.

With the easily accessible coupling partners **2** and **4** in hand, the key catalytic α-arylation step was investigated. The ketone **4** was found to be unstable in the presence of NaO*t*Bu, but pleasingly, the milder base Cs_2_CO_3_, in combination with 5 mol % [(Amphos)_2_PdCl_2_], facilitated coupling to provide **5** in an excellent yield of 84 %. Note that the reaction was performed in a sealed vial to allow temperature elevation to 90 °C, and to achieve full conversion of the aryl bromide it was necessary here (and in the following arylations) to use two equivalents of the ketone, which could be recovered easily after the reaction was complete. However, the product **5** could still be obtained in very good yield (74 %) when using just 1.2 equivalents of **4**.

As anticipated, treating **5** with NH_4_Cl in EtOH/H_2_O at 90 °C effected hydrolysis of the acetal and subsequent aromatization to provide the desired isoquinoline **6**. However, upon workup, the characteristic bright yellow color of berberine was obvious in the aqueous phase, thus indicating partial displacement of pivaloate by the isoquinoline nitrogen atom. This reactivity was therefore exploited to negate the need for pivaloate ester cleavage and hydroxy activation. Following formation of **6** from **5**, the direct displacement of pivaloate was promoted simply by increasing the reaction temperature from 90 °C to 110 °C, to give a smooth one-pot conversion into berberine in 82 % yield. Pure berberine chloride was obtained by adding NaOH to convert the isoquinolinium salt into its hydroxide adduct which was extracted with CH_2_Cl_2_.[1] Subsequent treatment with HCl regenerated the chloride salt.

Furthermore, the miscibility of THF, EtOH, and H_2_O permitted one-pot enolate arylation, isoquinoline formation, and cyclization of the B ring, thus providing berberine chloride from **2** and **4** in 40 % yield (equivalent to 74 % per step). Hence, the synthesis of berberine chloride was achieved in an overall yield of 68 % from compound **1** (the most valuable starting material) or 50 % from compound **3** (the longest linear sequence).

A key and advantageous feature of this modular route is the ability to access structurally diverse alkaloids simply by varying the aryl bromide and/or ketone coupling partners. This modularity is exemplified in the synthesis of pseudocoptisine by an identical route to that used for berberine, but starting from the alternative aryl bromide **7** (Scheme 3). Pseudocoptisine has been shown to inhibit acetylcholinesterase and possesses cognitive enhancing anti-amnesic properties.[10] One other synthesis has been reported, which invloves a photochemical enamide cyclization to form the C ring.[11]

**Scheme 3:**
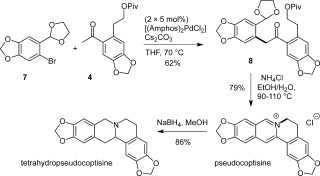
Synthesis of pseudocoptisine.

While previously reported NMR data for pseudocoptisine were obtained in CDCl_3_[10] we found our material to be insoluble in this solvent, as had others.[12] Data were obtained in both [D_6_]DMSO and CD_3_OD but a close match with literature data in CD_3_OD[13] was not made (this may be attributable to a difference in counterion, which is not described in the literature). Therefore, a known precursor and a tetrahydro derivative of pseudocoptisine were also synthesized separately (see the Supporting Information for details).[9,14] The ^1^H and ^13^C NMR data for these compounds were in agreement with those previously reported, and COSY, HSQC, and HMBC analyses of our synthetic pseudocoptisine were fully consistent with the published structure.

Our attention turned to the total synthesis of the C13-methylated protoberberine dehydrocorydaline,[15] which has previously been prepared once before[16] and also semi-synthesized by methylation of palmatine (Scheme 4).[17] It was anticipated that the application of in situ enolate functionalization methodology would allow the rapid introduction of this methyl group following enolate arylation.[7]

**Scheme 4:**
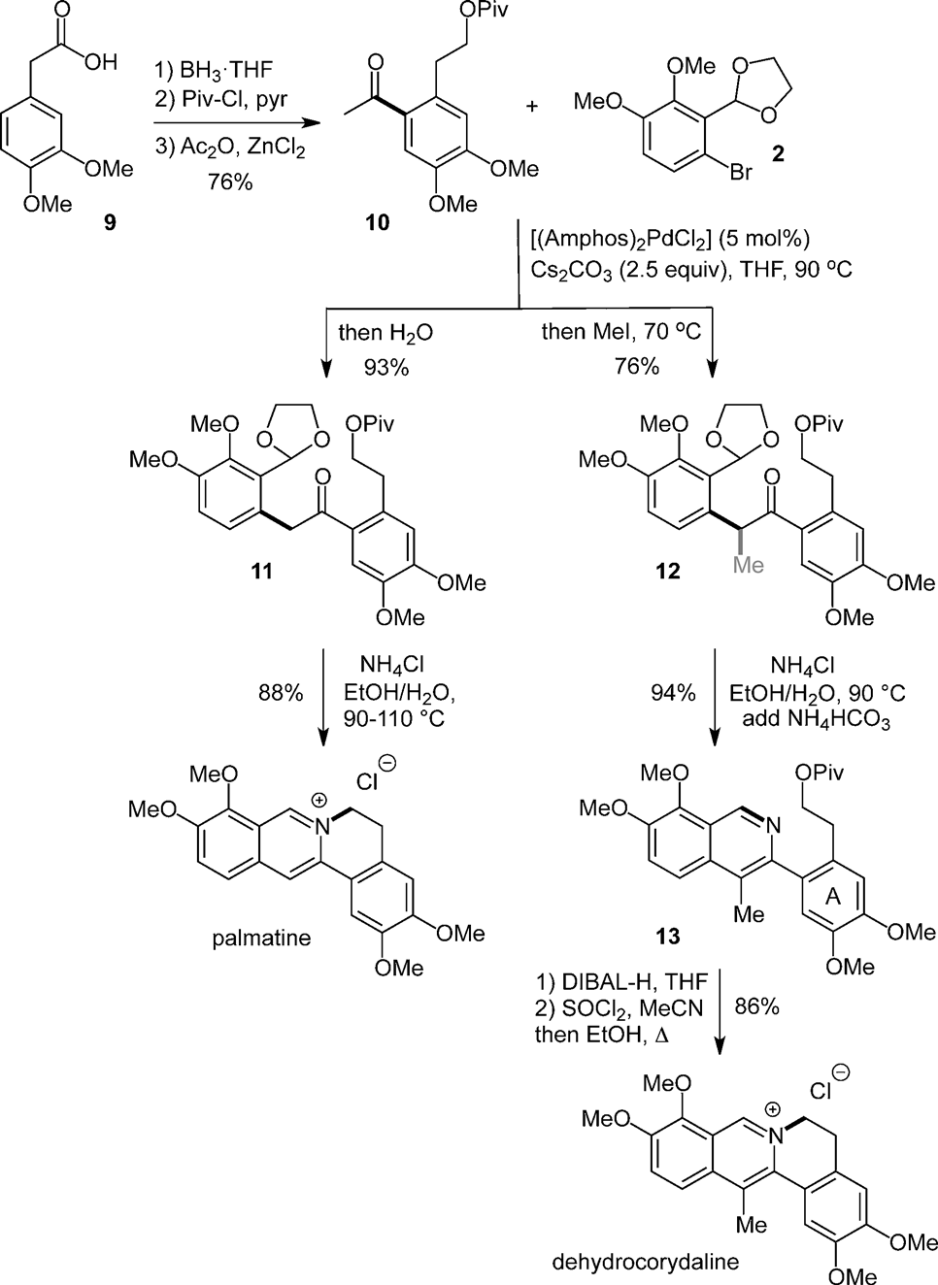
Palladium-catalyzed enolate coupling with in situ enolate derivatization using methyl iodide. DIBAL=diisobutylaluminum hydride

Before exploring this route, however, it was necessary to obtain the appropriate ketone coupling partner **10**, which was accomplished in 76 % overall yield by reduction, protection, and regioselective Friedel–Crafts acylation of the corresponding acid **9** (Scheme 4). The coupling of ketone **10** and aryl bromide **2** required no optimization, and was successful under identical reaction conditions to those used in the synthesis of berberine, thus highlighting the flexibility and synthetic utility of this route. Quenching the reaction with water provided the ketone **11** in 93 % yield, and in a single step, could be efficiently converted into the alkaloid palmatine in 88 % yield by our one-pot aromatization/cyclization protocol.[18] Pleasingly, as anticipated, in situ functionalization of the arylation product could easily be achieved through the addition of MeI to the reaction mixture, thus providing the α-methylated ketone **12** in 76 % yield.

Surprisingly at first, subjecting the masked 1,5-dicarbonyl **12** to the aromatization/cyclization conditions (NH_4_Cl, EtOH/H_2_O, 90–110 °C) did not facilitate a one-pot conversion into dehydrocorydaline (Scheme 4). It is likely that the presence of the C4-methyl group in the isoquinoline **13** restricts rotation about the isoquinoline–aryl bond, thereby disfavoring the near planar conformation required for displacement of pivaloate by the nitrogen atom. This hypothesis is supported by the ^1^H NMR spectrum of **13**, in which the CH_2_ protons adjacent to the A ring are diastereotopic. Reverting to our original proposal for protoberberine synthesis, **13** could be isolated in 94 % yield by the treating precursor **12** first with NH_4_Cl, which gave a mixture of **13** and an isochromene side product (see the Supporting Information for details), then basification with NH_4_HCO_3_ and further heating to convert the isochromene into **13**. To introduce a more effective leaving group and so increase the rate of cyclization, the pivaloate ester was removed by DIBAL-H reduction. Transformation of the alcohol to the chloride by the action of SOCl_2_ in MeCN, with subsequent solvent exchange and heating afforded dehydrocorydaline chloride in 86 % yield over three steps, and hence an overall yield of 47 % for the total synthesis. The spectroscopic data for this compound were a good match with those reported for the natural product.[15a,c]

Finally, the scope of this methodology was tested in the preparation of an unnatural, fluorinated, protoberberine analogue (**16**) by coupling the ketone **4** with aryl bromide **14** in 74 % yield (Scheme 5). The ability to access such analogues, with tunable electronic and steric properties, is potentially important from a medicinal chemistry perspective, particularly those that do not contain solely electron-rich aromatic rings. The susceptibility of the fluorinated isoquinoline core to decomposition, presumably by nucleophilic aromatic substitution, necessitated mild reaction conditions for formation of the B and C rings from **15**. To this end, acetal cleavage was conducted at 60 °C, followed by in situ aromatization at 90 °C, and conversion of the pivaloate ester into a chloride leaving group in the manner described for dehydrocorydaline. Nucleophilic displacement of chloride by nitrogen followed rapidly in the same vessel, with no need for solvent exchange or elevation of temperature. The unnatural analogue **16** was obtained in 50 % yield over this sequence from **15**.

**Scheme 5:**
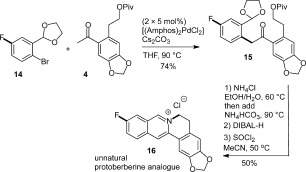
Formation of a fluorinated, unnatural analogue.

To conclude, berberine chloride was synthesized by a five-step (longest linear sequence) route in 50 % overall yield. This synthesis features a flexible palladium-catalyzed enolate arylation as the key step, and hence by substitution of the aryl bromide and ketone coupling partners, analogous syntheses of pseudocoptisine and palmatine (in 36 % and 62 % yields respectively), and of an unnatural fluorinated analogue (in 27 % yield) were readily achieved. Finally, enolate arylation was combined with in situ methylation to allow rapid derivatization of the alkaloid C13 position, thus resulting in the total synthesis of dehydrocorydaline in 47 % yield.
