# Supplementary material for: Short and Efficient Syntheses of Protoberberine Alkaloids using Palladium-Catalyzed Enolate Arylation
Source: Angew Chem Int Ed Engl. 2014 Oct 27;53(52):14555–8. doi: 10.1002/anie.201409164 (PMC4502971; doi:10.1002/anie.201409164)
Supplement: Supplementary file 1 [file anie0053-14555-sd1.pdf]

Angewandte Chemie
Eine Zeitschrift der Gesellschaft Deutscher Chemiker

Supporting Information

© Wiley-VCH 2014

69451 Weinheim, Germany

Short and Efficient Syntheses of Protoberberine Alkaloids using Palladium-Catalyzed Enolate Arylation**

*Alice E. Gatland, Ben S. Pilgrim, Panayiotis A. Procopiou, and Timothy J. Donohoe**

anie_201409164_sm_miscellaneous_information.pdf

Supporting Information

Table of Contents:

General Methods 2

General Procedures 3

Methods of Preparation and Spectroscopic Data for Compounds 3

NMR comparison tables 21

^{1}H NMR and ^{13}C NMR Spectra of Compounds 27

References 51

General Methods

^{1}H NMR, ^{13}C NMR and ^{19}F NMR spectra were recorded on a 400 MHz or 500 MHz spectrometer in $CDCl_3$, CD_3OD or DMSO-d_6 and referenced to residual solvent peaks or to $SiMe_4$ as an internal standard. Chemical shifts are quoted in ppm (parts per million) to the nearest 0.01 ppm (^{1}H NMR) or 0.1 ppm (^{13}C and ^{19}F NMR) with signal splittings recorded as singlet (s), doublet (d), triplet (t), quartet (q), multiplet (m) and broad (br.). Coupling constants, *J*, are measured in Hz to the nearest 0.1 Hz. ^{1}H, ^{13}C and ^{19}F NMR spectra were recorded at room temperature. Assignments were based upon DEPT, COSY, HSQC and HMBC experiments and by comparison of the spectral data with that of known compounds.

Infrared spectra were recorded neat on a Bruker Tensor 27 FT-IR spectrometer equipped with Attenuated Total Reflectance sampling accessories. Absorption maxima are quoted in wavenumbers (cm^{-1}) for the range 3500-1000 cm^{-1}. Mass spectra were recorded on a Bruker MicroTof (resolution = 10000 FWHM) under conditions of electrospray ionisation (ESI) or field ionisation (FI). Calibration was *via* the lock-mass of tetraoctyl ammonium bromide for positive ions and sodium dodecyl sulfate for negative ions. Melting points (m.p.) were obtained using a Lecia VMTG heated-stage microscope and are uncorrected. Flash column chromatography was performed using silica gel (60 Å, 0.033-0.070 mm, BDH) and TLC analyses were performed on Merck Kiesegel 60 F_{254} 0.25 mm precoated silica plates. Product spots were visualized under UV light (λ_{max} = 254 nm) and/or by staining with vanillin solution.

Reagents obtained from Sigma-Aldrich, Alfa, Fluorochem and TCI suppliers were used directly as supplied. Bis(di-*tert*-butyl(4-dimethylaminophenyl)phosphine)-dichloropalladium(II) ($(Amphos)_2PdCl_2$) was obtained from Johnson Matthey and stored in a desiccator. Compounds that contained acetals of electron-rich benzaldehydes were found to undergo slow hydrolysis (over a period of weeks) due to atmospheric moisture and hence were also stored in a desiccator. All anhydrous reactions were carried out in flame-dried glassware and under an inert atmosphere of argon. THF and MeCN were dried by purification through two activated alumina purification columns. Dry pyridine was used directly from Sure/Seal® bottles from Sigma-Aldrich.

General Procedures

General Procedure 1: Cyclic acetal formation

A solution of the aldehyde (1.0 eq.), ethylene glycol (1.5 eq.), and *para*-toluenesulfonic acid (0.02 eq.) in toluene (10 mL $mmol^{-1}$) was heated at reflux for 18 h using a Dean-Stark apparatus. After cooling to room temperature, the reaction was quenched by the addition of saturated aqueous $NaHCO_3$ (10 mL). The aqueous layer was extracted with Et_2O (3 × 10 mL) and the combined organic extracts were dried over $MgSO_4$, filtered and concentrated *in vacuo*. The crude residue was then purified as specified.

Methods of Preparation and Spectroscopic Data for Compounds

2-(6-Bromo-2,3-dimethoxyphenyl)-1,3-dioxolane (2)

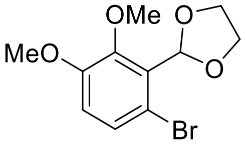

To a suspension of K_2CO_3 (558 mg, 4.04 mmol) in DMF (5.0 mL) was added 6-bromo-2-hydroxy-3-methoxybenzaldehyde (**1**) (467 mg, 2.02 mmol) and iodomethane (379 µL, 6.06 mmol) and the resulting mixture was stirred at 45 ºC for 18 h. After cooling to room temperature, the reaction was diluted with H_2O (20 mL) and quenched with a 1 M aqueous solution of HCl (20 mL). The resulting mixture was extracted with EtOAc (3 × 25 mL) and the combined organic extracts were washed with brine (2 × 50 mL), dried over Na_2SO_4, filtered and concentrated *in vacuo*. The crude residue was then subjected to **General Procedure 1**. Purification by flash column chromatography (SiO_2, petrol/EtOAc 9:1) afforded acetal **2** as a white solid (572 mg, 1.98 mmol, 98%).

M.p. 76-79 °C (lit. 79 °C); **^{1}H NMR** (400 MHz, $CDCl_3$) δ_H 7.29 (1 H, d, *J* = 8.8, *H*C(5)), 6.81 (1 H, d, *J* = 8.8, *H*C(4)), 6.35 (1 H, s, *H*C(OR)$_2$), 4.30-4.27 (2 H, m, OCH_aH$_b$CH_aH$_b$CO), 4.06-4.03 (2 H, m, OCH$_a$$H_bCH_a$$H_b$CO), 3.86 (3 H, s, OC$H_3$), 3.85 (3 H, s, OC$H_3$); **^{13}C NMR** (101 MHz, $CDCl_3$) δ_C 152.8, 150.2 (2 × C_{Ar}OMe), 129.4 (C_{Ar}), 129.1, 114.4 (2 × HC_{Ar}), 113.3 (C_{Ar}Br), 101.7 (*H*C(OR)$_2$), 65.9 (OCH$_2$$CH_2$O), 61.6, 56.1 (2 × O$CH_3$). Spectroscopic data were consistent with those previously reported.[1]

2-(Benzo[d][1,3]dioxol-5-yl)ethyl pivalate (S1)

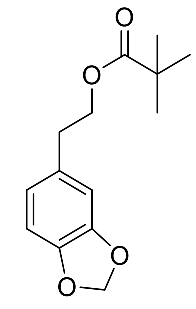

Borane-THF complex solution (11.7 mL, 11.7 mmol) was added slowly to a solution of 3,4-(methylenedioxy)phenylacetic acid (**3**) (1.05 g, 5.83 mmol) in THF (30 mL) at 0 ºC. The resulting solution was allowed to warm to room temperature and stirred for 2 h then quenched by the dropwise addition of a 1 M aqueous solution of NaOH (10 mL). The organic phase was washed with H_2O (2 × 50 mL) and brine (50 mL), dried over $MgSO_4$, filtered and concentrated *in vacuo*. The crude residue was then dissolved in CH_2Cl_2 (30 mL) and cooled to 0 ºC before pyridine (0.944 mL, 11.7 mmol) and pivaloyl chloride (1.44 mL, 11.7 mmol) were added sequentially. The reaction mixture was allowed to warm to room temperature and stirred for 18 h. The reaction was then diluted with H_2O (25 mL) and the resulting mixture was extracted with CH_2Cl_2 (3 × 25 mL). The combined organic extracts were dried over $MgSO_4$, filtered and concentrated *in vacuo*. Purification by flash column chromatography (SiO_2, 96:4 petrol/Et_2O) afforded pivalate **S1** as a colourless oil (1.34 g, 5.35 mmol, 92%).

^{1}H NMR (400 MHz, $CDCl_3$) δ_H 6.74-6.71 (2 H, m, 2 × HC_{Ar}), 6.65 (1 H, dd, J = 7.8 and 1.5, HC_{Ar}), 5.92 (2 H, s, OCH_2O), 4.22 (2 H, t, J = 6.9, CH_2OPiv), 2.85 (2 H, t, J = 6.8, CH_2CH_2OR), 1.18 (9 H, s, $(CH_3)_3$); **^{13}C NMR** (101 MHz, $CDCl_3$) δ_C 178.3 (C=O), 147.6, 146.1 (2 × $C_{Ar}OMe$), 131.7 (C_{Ar}), 121.8, 109.3, 108.1 (3 × HC_{Ar}), 100.8 (OCH_2O), 64.9 (CH_2OPiv), 38.6 ($C(CH_3)_3$), 34.8 (CH_2CH_2OPiv), 27.1 ($(CH_3)_3$); **IR** ν_{max} (neat)/cm^{-1} 2972, 1725, 1504, 1489, 1443, 1398, 1365, 1282, 1246, 1150, 1037; **HRMS** (ESI$^+$) $C_{14}H_{18}NaO_4$ requires 273.1097, found [M+Na$^+$] 273.1099 (–0.8 ppm).

2-(6-Acetylbenzo[d][1,3]dioxol-5-yl)ethyl pivalate (4)

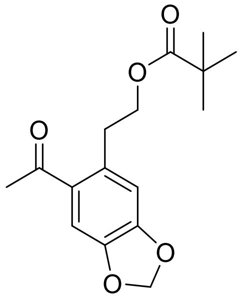

Zinc chloride (11.0 g, 80.5 mmol) was added to a solution of pivalate **S1** (4.02 g, 16.1 mmol) in acetic anhydride (40 mL) at 0 °C. The resulting suspension was allowed to warm to room temperature and stirred for 18 h. The reaction mixture was then concentrated *in vacuo* and the residue redissolved in EtOAc (100 mL), which was washed with a saturated, aqueous solution of $NaHCO_3$ (2 × 100 mL), H_2O (100 mL) and brine (100 mL). The organic phase was dried over $MgSO_4$, filtered and concentrated *in vacuo*. Purification by flash column chromatography (SiO_2, 92.5:7.5 petrol/EtOAc) afforded ketone **4** as a white solid (3.76 g, 12.9 mmol, 80%).

M.p. 51-53 °C; **^{1}H NMR** (400 MHz, $CDCl_3$) δ_H 7.22 (1 H, s, *H*C_{Ar}), 6.74 (1 H, s, *H*C_{Ar}), 6.01 (2 H, s, O*C*H_2O), 4.26 (2 H, t, *J* = 6.7, C*H*$_2$OPiv), 3.17 (2 H, t, *J* = 6.7, C*H*$_2$CH$_2$OPiv), 2.53 (3 H, s, C*H*$_3$C=O), 1.16, (9 H, s, (C*H*$_3$)$_3$); **^{13}C NMR** (101 MHz, $CDCl_3$) δ_C 199.0 (*C*=O), 178.4 (RO*C*=O), 150.0, 146.1, 135.1, 130.8 (4 × *C*$_{Ar}$), 112.3, 109.9 (2 × H*C*$_{Ar}$), 101.8 (O*C*H$_2$O), 64.9 (*C*H$_2$OPiv), 38.7 (*C*(CH$_3$)$_3$), 33.8 (*C*H$_2$CH$_2$OPiv), 29.4 (*C*H$_3$C=O), 27.2 ((*C*H$_3$)$_3$); **IR** ν_{max} (neat)/cm^{-1} 2972, 1715, 1681, 1611, 1504, 1479, 1372, 1270, 1247, 1157, 1109, 1036; **HRMS** (ESI$^+$) $C_{16}H_{20}NaO_5$ requires 315.1203, found [M+Na$^+$] 315.1202 (–0.2 ppm).

2-(6-(2-(2-(1,3-Dioxolan-2-yl)-3,4-dimethoxyphenyl)acetyl)benzo[d][1,3]dioxol-5-yl)ethyl pivalate (5)

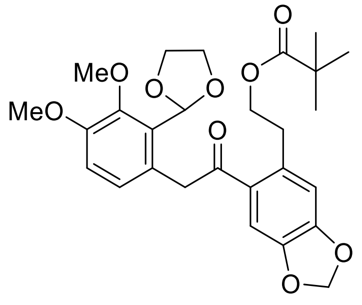

To a microwave vial fitted with a rubber septum was added aryl bromide **2** (308 mg, 1.07 mmol), ketone **4** (623 mg, 2.13 mmol), Cs_2CO_3 (868 mg, 2.66 mmol) and (Amphos)$_2$PdCl$_2$ (37.9 mg, 0.0535 mmol). The septum was replaced with a microwave cap and the vessel was evacuated and backfilled with nitrogen before the solids were dissolved in anhydrous THF (5.4 mL). The reaction mixture was stirred at 90 ºC for 18 h then cooled to room temperature and quenched with H_2O (5 mL). The resulting mixture was extracted with Et_2O (3 × 10 mL) and the combined organic extracts were dried over $MgSO_4$, filtered and concentrated *in vacuo*. Purification by automated flash column chromatography (70 g SiO_2 cartridge, 0-50% EtOAc/cyclohexane) afforded ketone **5** as an off-white solid (447 mg, 0.893 mmol, 84%).

M.p. 124-127 °C; **^{1}H NMR** (400 MHz, $CDCl_3$) δ_H 7.32 (1 H, s, *H*C_{Ar}), 6.95-6.89 (2 H, m, 2 × *H*C_{Ar}), 6.77 (1 H, s, *H*C_{Ar}), 6.12 (1 H, s, *H*C(OR)$_2$), 6.02 (2 H, s, OC*H*$_2$O), 4.29 (2 H, t, *J* = 6.6, C*H*$_2$OPiv), 4.26 (2 H, s, C*H*$_2$C=O), 3.86-3.75 (10 H, m, 2 × OC*H*$_3$ and OC*H*$_2$C*H*$_2$O), 3.13 (2 H, t, *J* = 6.6, C*H*$_2$CH$_2$OPiv), 1.16 (9 H, s, (C*H*$_3$)$_3$); **^{13}C NMR** (101 MHz, $CDCl_3$) δ_C 198.8 (*C*=O), 178.4 (RO*C*=O), 151.5, 149.6, 149.3, 145.9, 135.2, 131.1 (6 × *C*$_{Ar}$), 128.3 (*H*C$_{Ar}$), 127.5, 127.0 (2 × *C*$_{Ar}$), 113.1, 112.3, 108.8 (3 × H*C*$_{Ar}$), 101.7 (O*C*H$_2$O), 99.3 (H*C*(OR)$_2$), 65.2 (*C*H$_2$OPiv), 64.6 (O*C*H$_2$*C*H$_2$O), 61.7, 55.7 (2 × O*C*H$_3$), 44.5 (*C*H$_2$C=O), 38.6 (*C*(CH$_3$)$_3$), 33.6 (*C*H$_2$CH$_2$OPiv), 27.2 ((*C*H$_3$)$_3$); **IR** ν_{max} (neat)/cm^{-1} 2965, 1717, 1687, 1493, 1379, 1271, 1241, 1149, 1083, 1062, 1043; **HRMS** (ESI$^+$) $C_{27}H_{32}NaO_9$ requires 523.1939, found [M+Na$^+$] 523.1917 (+4.0 ppm).

2-(6-(7,8-Dimethoxyisoquinolin-3-yl)benzo[d][1,3]dioxol-5-yl)ethyl pivalate (6)

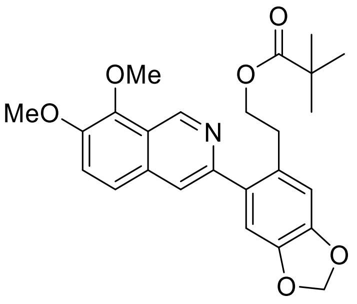

To a microwave vial containing ketone **5** (83.1 mg, 0.166 mmol) was added a 1 M solution of NH_4Cl in 3:1 EtOH/H_2O (1.7 mL) and the resulting mixture was stirred at 90 °C for 18 h. After cooling to room temperature, the reaction was diluted with H_2O (5 mL) and the resulting mixture was extracted with EtOAc (3 × 10 mL). The combined organic extracts were dried over $MgSO_4$, filtered and concentrated *in vacuo.* Purification by automated flash column chromatography (10 g SiO_2 cartridge, 0-50% EtOAc/cyclohexane) afforded isoquinoline **6** as a pale yellow oil (53.1 mg, 0.121 mmol, 73%).

^{1}H NMR (400 MHz, $CDCl_3$) δ_H 9.58 (1 H, s, *H*C(1)), 7.63 (1 H, d, *J* = 0.8, *H*C(4)), 7.59 (1 H, d, *J* = 8.9, *H*C(6)), 7.51 (1 H, d, *J* = 8.9, *H*C(5)), 6.95 (1 H, s, HC_{Ar}), 6.86 (1 H, s, HC_{Ar}), 5.98 (2 H, s, OCH_2O), 4.18 (2 H, t, *J* = 6.9, CH_2OPiv), 4.11 (3 H, s, OCH_3), 4.02 (3 H, s, OCH_3), 3.01 (2 H, t, *J* = 6.9, CH_2CH_2OPiv), 1.14 (9 H, s, $(CH_3)_3$); **^{13}C NMR** (101 MHz, $CDCl_3$) δ_C 178.4 (*C*=O), 151.4 (*C*(3)), 148.8, 147.3 (2 × C_{Ar}), 146.7 (H*C*(1)), 146.2, 144.0, 134.4, 132.2, 130.2 (5 × C_{Ar}), 122.8 (H*C*(6)), 122.7 (C_{Ar}), 120.5 (H*C*(5)), 119.4 (H*C*(4)), 110.5 (2 × HC_{Ar}), 101.1 (OCH_2O), 65.0 (CH_2OPiv), 61.6 (OCH_3), 57.1 (OCH_3), 38.6 ($C(CH_3)_3$), 32.3 (CH_2CH_2OPiv), 27.1 ($(CH_3)_3$); **IR** ν_{max} (neat)/cm^{-1} 2969, 1721, 1585, 1567, 1494, 1371, 1281, 1260, 1223, 1151, 1115, 1093, 1036; **HRMS** (ESI$^+$) $C_{25}H_{28}NO_6$ requires 438.1911, found [M+H$^+$] 438.1918 (−1.3 ppm).

Berberine chloride

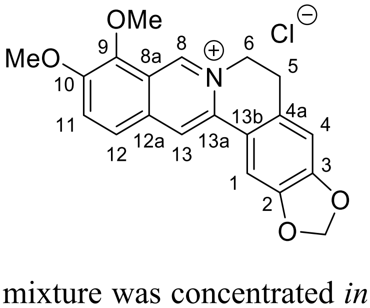

Method A: To a microwave vial containing ketone **5** (72.5 mg, 0.145 mmol) was added a 1 M solution of NH_4Cl in 3:1 EtOH/H_2O (1.5 mL) and the resulting mixture was stirred at 90 °C for 18 h. The temperature was then increased to 110 °C and the reaction was stirred for a further 48 h. The reaction mixture was concentrated *in vacuo* and the residue redissolved in H_2O (10 mL). A 20% w/w aqueous solution of NaOH (10 mL) was added and the solution was stirred for 5 min then extracted with CH_2Cl_2 (5 × 15 mL). The combined organic extracts were poured into a separating funnel containing H_2O (20 mL) before 1 M aqueous HCl (20 mL) was added to regenerate berberine chloride. The resulting mixture was washed with H_2O (5 × 20 mL) and the aqueous washes were combined and concentrated *in vacuo*. The solid residue was

triturated with CH_2Cl_2 (2 × 5 mL) to afford berberine chloride as a bright yellow solid (44.2 mg, 0.119 mmol, 82%).

Method B: To a microwave vial fitted with a rubber septum was added aryl bromide **2** (200 mg, 0.692 mmol), ketone **4** (403 mg, 1.38 mmol), Cs_2CO_3 (564 mg, 1.73 mmol) and $(Amphos)_2PdCl_2$ (24.5 mg, 0.0346 mmol). The septum was replaced with a microwave cap and the vessel was evacuated and backfilled with nitrogen before the solids were dissolved in anhydrous THF (3.5 mL). The reaction mixture was stirred at 90 ºC for 18 h then cooled to room temperature. A 1 M solution of NH_4Cl in 3:1 EtOH/H_2O (10.4 mL, 10.4 mmol) was then added and the reaction was stirred at 90 °C for a further 24 h, then at 110 °C for 72 h. After cooling to room temperature, the suspension was diluted with Et_2O and filtered. The solid was washed with cold H_2O (5 × 5 mL) then dissolved in MeOH (15 mL). The MeOH solution was concentrated *in vacuo* and the resulting solid was triturated with Et_2O (2 × 5 mL), EtOAc (2 × 5 mL) and cold H_2O (2 × 2 mL) then redissolved in MeOH (15 mL). Concentration *in vacuo* afforded berberine chloride as a bright yellow solid (103 mg, 0.277 mmol, 40%).

M.p. decomposed 207 °C (lit. 203-205 °C); **^{1}H NMR** (400 MHz, DMSO-d_6) δ_H 9.90 (1 H, s, *H*C(8)), 8.97 (1 H, s, *H*C(13)), 8.19 (1 H, d, *J* = 9.1, *H*C(11)), 8.01 (1 H, d, *J* = 9.1, *H*C(12)), 7.79 (1 H, s, *H*C(1)), 7.08 (1 H, s, *H*C(4)), 6.17 (2 H, s, OCH_2O), 4.95 (2 H, t, *J* = 6.3, C(6)H_2), 4.10 (3 H, s, C(9)OCH_3), 4.07 (3 H, s, C(10)OCH_3), 3.21 (2 H, t, *J* = 6.2, C(5)H_2); **^{13}C NMR** (101 MHz, DMSO-d_6) δ_C 150.3 (*C*(10)), 149.8 (*C*(3)), 147.6 (*C*(2)), 145.4 (H*C*(8)), 143.6 (*C*(9)), 137.4 (*C*(13a)), 133.0 (*C*(12a), 130.6 (*C*(4a)), 126.7 (H*C*(11)), 123.5 (H*C*(12)), 121.4 (*C*(8a)), 120.4 (*C*(13b)), 120.2 (H*C*(13)), 108.4 (H*C*(4)), 105.4 (H*C*(1)), 102.0 (O*C*H_2O), 61.9 (C(9)O*C*H_3), 57.1 (C(10)O*C*H_3), 55.1 (*C*(6)H_2), 26.3 (*C*(5)H_2). Spectroscopic data were consistent with those previously reported.[2,3]

5-Bromo-6-(1,3-dioxolan-2-yl)benzo[d][1,3]dioxole (7)

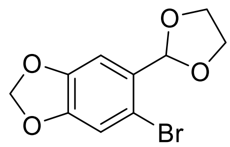

6-Bromo-1,3-benzodioxole-5-carboxaldehyde (2.05 g, 8.68 mmol) was subjected to **General Procedure 1**. Purification by automated flash column chromatography (100 g SiO_2 cartridge, 0-25% TBME/cyclohexane) afforded acetal **7** as a white solid (2.20 g, 8.06 mmol, 93%).

M.p. 64-68 °C (lit. 68-69 °C); **^{1}H NMR** ($CDCl_3$, 400 MHz) δ_H 7.08 (1 H, s, *H*C_{Ar}), 7.00 (1 H, s, *H*C_{Ar}), 6.02 (1 H, s, *H*C(OR)$_2$), 5.99 (2 H, s, OCH_2O), 4.18-4.10 (2 H, m, OC$H_a$$H_bCH_a$$H_b$O), 4.09-4.01 (2 H, m, OC$H_a$$H_bCH_a$$H_b$O); **^{13}C NMR** ($CDCl_3$, 101 MHz) δ_C

149.0 (C_{Ar}OR), 147.5 (C_{Ar}OR), 130.0 (C_{Ar}), 113.9 (C_{Ar}Br), 112.8 (HC_{Ar}), 107.7 (HC_{Ar}), 102.6 (H*C*(OR)$_2$), 101.9 (O*C*H$_2$O), 65.4 (O*C*H$_2$*C*H$_2$O). Spectroscopic data were consistent with those previously reported.[4]

2-(6-(2-(6-(1,3-Dioxolan-2-yl)benzo[d][1,3]dioxol-5-yl)acetyl)benzo[d][1,3]dioxol-5-yl)ethyl pivalate (8)

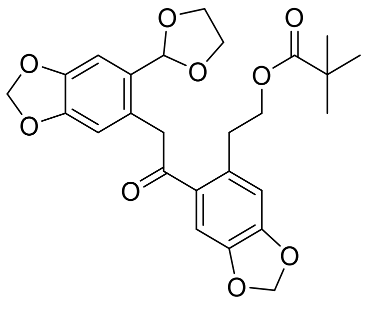

To a microwave vial fitted with a rubber septum was added aryl bromide **7** (119 mg, 0.436 mmol), ketone **4** (255 mg, 0.872 mmol), Cs_2CO_3 (355 mg, 1.08 mmol) and $(Amphos)_2PdCl_2$ (15.4 mg, 0.0218 mmol). The septum was replaced with a microwave cap and the vessel was evacuated and backfilled with argon before the solids were dissolved in anhydrous THF (2.2 mL). The reaction mixture was stirred at 70 ºC for 24 h then cooled to room temperature. The crimped cap was removed and replaced with a rubber septum and the flask was purged with argon whilst additional $(Amphos)_2PdCl_2$ (15.4 mg, 0.0218 mmol) was added. A new microwave cap was fitted and the vessel purged with argon for 5 mins before stirring at 70 °C for a further 24 h. After cooling to room temperature, the reaction was quenched with H_2O (5 mL). The resulting mixture was extracted with EtOAc (3 × 10 mL) and the combined organic extracts were dried over $MgSO_4$, filtered and concentrated *in vacuo*. Purification by flash column chromatography (SiO_2, 8:2 petrol/EtOAc) afforded ketone **8** as an off-white solid (130 mg, 0.268 mmol, 62%).

M.p. 119-121 °C; **^{1}H NMR** (400 MHz, $CDCl_3$) δ_H 7.34 (1 H, s, *H*C_{Ar}), 7.07 (1 H, s, *H*C_{Ar}), 6.77 (1 H, s, *H*C_{Ar}), 6.65 (1 H, s, *H*C_{Ar}), 6.03 (2 H, s, OC*H*$_2$O), 5.95 (2 H, s, OC*H*$_2$O), 5.75 (1 H, s, *H*C(OR)$_2$), 4.25 (2 H, s, C*H*$_2$C=O), 4.25 (2 H, t, J = 6.6, C*H*$_2$OPiv), 4.00-3.90 (4 H, m, OC*H*$_2$C*H*$_2$O), 3.10 (2 H, t, J = 6.6, C*H*$_2$CH$_2$OPiv), 1.16 (9 H, s, (C*H*$_3$)$_3$); **^{13}C NMR** (101 MHz, $CDCl_3$) δ_C 198.5 (*C*=O), 178.3 (RO*C*=O), 149.9, 148.0, 146.6, 146.1 (4 × C_{Ar}OR), 135.3, 130.6, 129.3, 127.4 (4 × C_{Ar}), 112.4, 111.5, 109.1, 107.3 (4 × HC_{Ar}), 102.1 (H*C*(OR)$_2$), 101.8, 101.2 (2 × O*C*H$_2$O), 65.0 (*C*H$_2$OPiv), 64.9 (O*C*H$_2$*C*H$_2$O), 44.6 (*C*H$_2$C=O), 38.6 (*C*(CH$_3$)$_3$), 33.7 (*C*H$_2$CH$_2$OPiv), 27.2 ((*C*H$_3$)$_3$); **IR** ν_{max} (neat)/cm^{-1} 2923, 1722, 1681, 1610, 1502, 1487, 1371, 1263, 1241, 1155, 1100, 1078, 1031; **HRMS** (ESI$^+$) $C_{26}H_{28}NaO_9$ requires 507.1626, found [M+Na$^+$] 507.1618 (+1.1 ppm).

Pseudocoptisine chloride

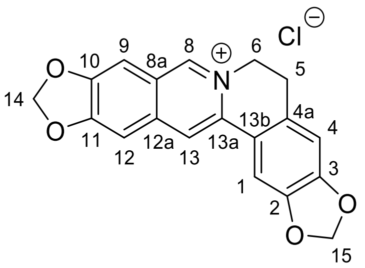

To a screw-capped tube containing ketone **8** (59.1 mg, 0.122 mmol) was added a 1 M solution of NH_4Cl in 3:1 EtOH/H_2O (1.2 mL) and the resulting mixture was stirred at 90 °C for 18 h. The temperature was then increased to 110 °C and the reaction was stirred for a further 72 h. The reaction mixture was concentrated *in vacuo* and the residue redissolved in H_2O (5 mL). A 20% w/w aqueous solution of NaOH (10 mL) was added and the solution was stirred for 5 min then extracted with CH_2Cl_2 (3 × 15 mL). The combined organic extracts were poured into a separating funnel containing 1 M aqueous HCl (20 mL) and the resulting mixture was washed with 1 M HCl (3 × 20 mL). The combined aqueous washes were concentrated *in vacuo* and the solid residue was triturated with Et_2O (5 × 10 mL) to afford afford pseudocoptisine chloride as a pale yellow solid (34.4 mg, 0.0967 mmol, 79%).

M.p. decomposed 318 °C (lit. decomposed 300 °C)[5]; **^{1}H NMR** (400 MHz, DMSO-d$_6$) δ_H 9.58 (1 H, s, *H*C(8)), 8.78 (1 H, s, *H*C(13)), 7.75 (1 H, s, *H*C(1)), 7.72 (1 H, s, *H*C(9)), 7.54 (1 H, s, *H*C(12)), 7.10 (1 H, s, *H*C(4)), 6.42 (2 H, s, C(15)H_2), 6.17 (2 H, s, C(14)H_2), 4.76 (2 H, t, J = 6.2, C(6)H_2), 3.19 (2 H, t, J = 6.3, C(5)H_2); **^{1}H NMR** (500 MHz, CD_3OD) δ_H 9.31 (1 H, s, *H*C(8)), 8.55 (1 H, s, *H*C(13)), 7.64 (1 H, s, *H*C(1)), 7.55 (1 H, s, *H*C(9)), 7.51 (1 H, s, *H*C(12)), 6.96 (1 H, s, *H*C(4)), 6.35 (2 H, s, C(15)H_2), 6.11 (2 H, s, C(14)H_2), 4.78 (2 H, t, J = 6.5, C(6)H_2), 3.23 (2 H, t J = 6.4, C(5)H_2); **^{13}C NMR** (101 MHz, DMSO-d$_6$) δ_C 156.4 (*C*(10)), 151.3 (*C*(11)), 150.5 (*C*(2)), 148.1 (*C*(3)), 146.3 (H*C*(8)), 139.3 (*C*(8a)), 139.1 (*C*(13a)), 131.3 (*C*(4a)), 124.0 (*C*(12a)), 120.8 (*C*(13b)), 119.4 (H*C*(13)), 109.0 (H*C*(4)), 105.9 (H*C*(1)), 104.4 (*C*(15)H_2), 104.2 (H*C*(9)), 103.1 (H*C*(12)), 102.6 (*C*(14)H_2), 54.9 (*C*(6)H_2), 26.8 (*C*(5)H_2); **^{13}C NMR** (126 MHz, CD_3OD) δ_C 158.5 (*C*(10)), 153.3 (*C*(11)), 152.5 (*C*(2)), 150.1 (*C*(3)), 146.7 (H*C*(8)), 141.3 (*C*(8a)), 141.0 (*C*(13a)), 132.2 (*C*(13b)), 125.8 (*C*(12a)), 121.9 (*C*(4a)), 120.4 (H*C*(13)), 109.6 (H*C*(4)), 106.7 (H*C*(1)), 105.6 (*C*(15)H_2), 104.9 (H*C*(9)), 104.2 (H*C*(12)), 103.9 (*C*(14)H_2), 56.4 (*C*(6)H_2), 28.3 (*C*(5)H_2); **IR** ν_{max} (neat)/cm^{-1} 3385, 1613, 1495, 1458, 1369, 1270, 1233, 1207, 1189, 1098, 1033; **HRMS** (ESI$^+$) $C_{19}H_{14}NO_4$ requires 320.0917, found [M^+] 320.0914 (+0.6 ppm).

2-(6-([1,3]Dioxolo[4,5-g]isoquinolin-7-yl)benzo[d][1,3]dioxol-5-yl)ethan-1-ol (S2)

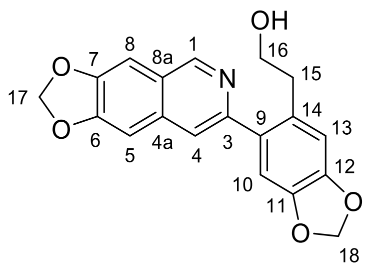

To a screw-capped tube containing ketone **8** (83.8 mg, 0.173 mmol) was added a 1 M solution of NH_4Cl in 3:1 EtOH/H_2O (1.7 mL) and the resulting mixture was stirred at 90 °C for 18 h. After cooling to room temperature, the reaction was diluted with H_2O (5 mL) and the resulting mixture was extracted with EtOAc (3 × 5 mL). The combined organic extracts were dried over $MgSO_4$, filtered and concentrated *in vacuo*. The resulting residue was redissolved in anhydrous THF (1.7 mL) and cooled to −78 °C before a 1 M solution of DIBAL-H in hexanes (433 µL, 0.433 mmol) was added slowly *via* syringe. The reaction mixture was warmed to room temperature and stirred for 4 h then quenched with H_2O (2 mL). The resulting mixture was extracted with EtOAc (3 × 5 mL) and the combined organic extracts were dried over $MgSO_4$, filtered and concentrated *in vacuo*. Purification by flash column chromatography (SiO_2, 1:1 petrol/Et_2O) afforded isoquinoline **S2** as a white solid (33.7 mg, 0.0999 mmol, 58%).

M.p. 184-186 °C; **^{1}H NMR** (400 MHz, ($CDCl_3$) δ_H 8.95 (1 H, s, *H*C(1)), 7.63 (1 H, s, *H*C(4)), 7.22 (1 H, s, *H*C(8)), 7.10 (1 H, s, *H*C(5)), 6.91 (1 H, s, *H*C(10)), 6.87 (1 H, s, *H*C(13)), 6.13 (2 H, s, C(17)H_2), 6.00 (2 H, s, C(18)H_2), 3.98 (2 H, t, J = 5.6, C(16)H_2), 2.82 (2 H, t, J = 5.6, C(15)H_2); **^{13}C NMR** (101 MHz, ($CDCl_3$) δ_C 151.6 (*C*(7)), 151.4 (*C*(3)), 148.6 (*C*(6)), 148.1 (*C*(12)), 147.9 (H*C*(1)), 146.1 (*C*(11)), 135.5 (*C*(8a)), 133.5 (*C*(9)), 132.7 (*C*(14)), 124.4 (*C*(4a)), 120.3 (H*C*(4)), 110.0 (H*C*(13)), 109.9 (H*C*(10)), 103.0 (H*C*(8)), 102.5 (H*C*(5)), 101.8 (*C*(17)H_2), 101.2 (*C*(18)H_2), 63.8 (*C*(16)H_2), 35.2 (*C*(15)H_2); **IR** ν_{max} (neat)/cm^{-1} 3174, 2907, 2850, 1600, 1503, 1482, 1453, 1234, 1039; **HRMS** (ESI$^+$) $C_{19}H_{16}NO_5$ requires 338.1023, found [M+H$^+$] 338.1014 (−2.5 ppm). NMR data were consistent with those previously reported.[6]

Tetrahydropseudocoptisine

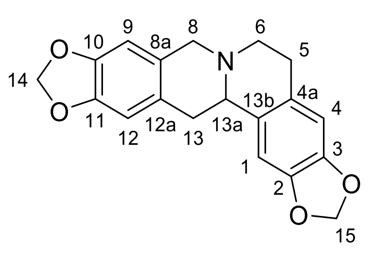

To a solution of pseudocoptisine (17.5 mg, 0.0492 mmol) in MeOH (2.0 mL) at 45 °C was added $NaBH_4$ (7.45 mg, 0.197 mmol) portionwise. The reaction mixture was allowed to cool to room temperature and stirred for 2 h then concentrated *in vacuo*. The residue was dissolved in $CHCl_3$ (5 mL) and poured into a separating funnel containing H_2O (5 mL). The resulting mixture was extracted with $CHCl_3$ (4 × 5 mL) and the combined organic extracts were dried over $MgSO_4$, filtered

and concentrated *in vacuo*. Purification by flash column chromatography (SiO_2, $CHCl_3$) afforded tetrahydropseudocoptisine as a white solid (13.7 mg, 0.0424 mmol, 86%).

M.p. 214-217 °C (lit. 212-214 °C);[7] **^{1}H NMR** (400 MHz, ($CDCl_3$) δ_H 6.73 (1 H, s, *H*C(1)), 6.62 (1 H, s, *H*C(12)), 6.59 (1 H, s, *H*C(4)), 6.55 (1 H, s, *H*C(9)), 5.92 (2 H, s, C(15)H_2), 5.91 (2 H, s, C(14)H_2), 3.91 (1 H, d, J = 14.6, C(8)H_aH$_b$), 3.64 (1 H, d, J = 14.6, C(8)H$_a$$H_b$), 3.54 (1 H, dd, J = 11.2, 3.6, *H*C(13a)), 3.17 (1 H, dd, J = 16.1, 3.9, C(13)H_aH$_b$), 3.14-3.07 (2 H, m, C(5)H_aH$_b$ and C(6)H_aH$_b$), 2.80 (1 H, dd, J = 15.7, 11.4, C(13)H$_a$$H_b$), 2.71-2.54 (2 H, m, C(5)H$_a$$H_b$ and C(6)H$_a$$H_b$); **^{13}C NMR** (101 MHz, ($CDCl_3$) δ_C 146.1, 146.1, 145.9, 145.8 (*C*(2), *C*(3), *C*(10), *C*(11)), 130.7 (*C*(4a)), 127.7 (*C*(13b)), 127.0 (*C*(8a), *C*(12a)), 108.4 (H*C*(12)), 108.4 (H*C*(4)), 106.0 (H*C*(9)), 105.4 (H*C*(1)), 100.8 (*C*(15)H_2), 100.6 (*C*(14)H_2), 59.8 (H*C*(13a)), 58.5 (*C*(8)H_2), 51.2 (*C*(6)H_2), 36.9 (*C*(13)H_2), 29.5 (*C*(5)H_2); **IR** ν_{max} (neat)/cm^{-1} 2903, 2795, 2746, 1483, 1389, 1345, 1248, 1228, 1162, 1127, 1034; **HRMS** (ESI$^+$) $C_{19}H_{18}NO_4$ requires 324.1230, found [M+H$^+$] 324.1228 (−0.8 ppm). NMR data were consistent with those previously reported.[8,9]

3,4-Dimethoxyphenethyl pivalate (S3)

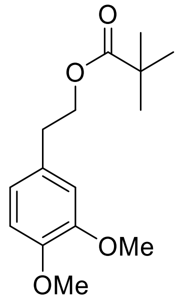

Borane-THF complex solution (11.3 mL, 11.3 mmol) was added slowly to a solution of 3,4-dimethoxyphenylacetic acid (**9**) (1.11 g, 5.66 mmol) in THF (30 mL) at 0 ºC. The resulting solution was allowed to warm to room temperature and stirred for 2 h then quenched by the dropwise addition of a 1 M aqueous solution of NaOH (10 mL). The organic phase was washed with H_2O (2 × 25 mL) and brine (25 mL), dried over $MgSO_4$, filtered and concentrated *in vacuo*. The crude residue was then dissolved in CH_2Cl_2 (30 mL) and cooled to 0 ºC before pyridine (0.912 mL, 11.3 mmol) and pivaloyl chloride (1.39 mL, 11.3 mmol) were added. The reaction mixture was allowed to warm to room temperature and stirred for 18 h then diluted with H_2O (25 mL). The resulting mixture was extracted with CH_2Cl_2 (3 × 25 mL) and the combined organic extracts were dried over $MgSO_4$, filtered and concentrated *in vacuo*. Purification by flash column chromatography (SiO_2, 8:2 petrol/Et_2O) afforded pivalate **S3** as a white solid (1.40 g, 5.23 mmol, 92%).

M.p. 46-50 °C; **^{1}H NMR** (400 MHz, $CDCl_3$) δ_H 6.82-6.80 (1 H, m, *H*C$_{Ar}$), 6.77-6.75 (2 H, m, 2 × *H*C$_{Ar}$), 4.25 (2 H, t, J = 6.9, CH_2OPiv), 3.87 (3 H, s, OCH_3), 3.86 (3 H, s, OCH_3), 2.88 (2 H, t, J = 7.0, CH_2CH$_2$OPiv); **^{13}C NMR** (101 MHz, $CDCl_3$) δ_C 178.4 (*C*=O), 148.8, 147.6 (2 × *C*$_{Ar}$OMe), 130.5 (*C*$_{Ar}$), 120.9 (H*C*$_{Ar}$), 112.1, 111.2 (2 × H*C*$_{Ar}$), 65.0 (*C*H$_2$OPiv), 55.8,

55.7 (2 × OCH$_3$), 38.6 (C(CH$_3$)), 34.7 (CH$_2$CH$_2$OPiv), 27.1 ((CH$_3$)$_3$); **IR** ν_{max} (neat)/cm^{-1} 2961, 1721, 1589, 1515, 1467, 1284, 1258, 1234, 1151, 1029; **HRMS** (ESI$^+$) $C_{15}H_{22}NaO_4$ requires 289.1410, found [M+Na$^+$] 289.1416 (–1.6 ppm).

2-Acetyl-4,5-dimethoxyphenethyl pivalate (10)

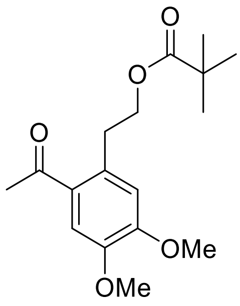

Zinc chloride (9.40 g, 69.0 mmol) was added to a solution of pivalate **S3** (3.67 g, 13.8 mmol) in acetic anhydride (35 mL) at 0 °C. The resulting suspension was allowed to warm to room temperature and stirred for 18 h. The reaction mixture was concentrated *in vacuo* and the residue redissolved in EtOAc (100 mL), which was then washed with a saturated, aqueous solution of $NaHCO_3$ (2 × 100 mL), H_2O (100 mL) and brine (100 mL). The organic phase was dried over $MgSO_4$, filtered and concentrated *in vacuo*. Purification by flash column chromatography (SiO_2, 8:2 petrol/Et_2O) afforded ketone **10** as a colourless oil (3.53 g, 11.4 mmol, 83%).

^{1}H NMR (400 MHz, $CDCl_3$) δ_H 7.24 (1 H, s, *H*C(3)), 6.73 (1 H, s, *H*C(6)), 4.25 (2 H, t, J = 6.7, CH_2OPiv), 3.89 (6 H, s, 2 × OCH_3), 3.18 (2 H, t, J = 6.7, CH_2CH$_2$OPiv), 2.55 (3 H, s, CH_3C=O), 1.12 (9 H, s, (CH_3)$_3$); **^{13}C NMR** (101 MHz, $CDCl_3$) δ_C 199.1 (C=O), 178.3 (ROC=O), 151.4, 146.7 (2 × C_{Ar}OMe), 133.3, 129.2 (2 × C_{Ar}), 114.8 (HC(6)), 113.4 (HC(3)), 64.8 (CH$_2$OPiv), 56.0, 55.8 (2 × OCH$_3$), 38.5 (C(CH$_3$)$_3$), 33.6 (CH$_2$CH$_2$OPiv), 29.2 (CH$_3$C=O), 27.1 ((CH$_3$)$_3$); **IR** ν_{max} (neat)/cm^{-1} 2969, 1722, 1673, 1604, 1568, 1518, 1462, 1360, 1266, 1206, 1148, 1058; **HRMS** (ESI$^+$) $C_{17}H_{24}NaO_5$ requires 331.1516, found [M+Na$^+$] 331.1526 (–3.8 ppm).

2-(2-(2-(1,3-Dioxolan-2-yl)-3,4-dimethoxyphenyl)acetyl)-4,5-dimethoxyphenethyl pivalate (11)

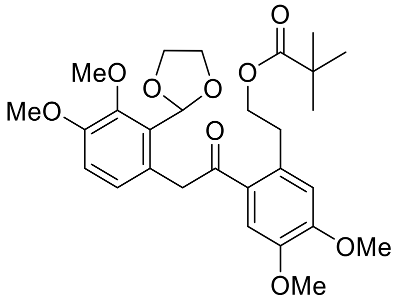

To a microwave vial fitted with a rubber septum was added aryl bromide **2** (196 mg, 0.679 mmol), Cs_2CO_3 (553 mg, 1.70 mmol) and (Amphos)$_2$PdCl$_2$ (24.1 mg, 0.0340 mmol). The septum was replaced with a microwave cap and the vessel was evacuated and backfilled with argon before a solution of ketone **10** (419 mg, 1.36 mmol) in anhydrous THF (3.4 mL) was added *via* syringe. The reaction mixture was stirred at 90 ºC for 18 h then cooled to room temperature and quenched with H_2O (5 mL). The resulting mixture was extracted with EtOAc (3 × 10 mL) and the combined organic extracts were dried

over $MgSO_4$, filtered and concentrated *in vacuo*. Purification by flash column chromatography (SiO_2, 7:3 petrol/EtOAc) afforded ketone **11** as an off-white solid (325 mg, 0.629 mmol, 93%).

M.p. 130-134 °C; **^{1}H NMR** (400 MHz, $CDCl_3$) δ_H 7.34 (1 H, s, HC_{Ar}), 6.91-6.86 (2 H, m, 2 × HC_{Ar}), 6.76 (1 H, s, HC_{Ar}), 6.14 (1 H, s, $HC(OR)_2$), 4.31 (2 H, s, CH_2C=O), 4.31 (2 H, t, J = 6.7, CH_2OPiv), 3.91 (3 H, s, OCH_3), 3.87-3.75 (4 H, m, OCH_2CH_2O), 3.85 (3 H, s, OCH_3), 3.84 (3 H, s, OCH_3), 3.81 (3 H, s, OCH_3), 3.19 (2 H, t, J = 6.7, CH_2CH_2OPiv), 1.15 (9 H, s, $(CH_3)_3$); **^{13}C NMR** (101 MHz, $CDCl_3$) δ_C 199.2 (C=O), 178.4 (ROC=O), 151.3, 150.9, 149.1, 146.6 (4 × C_{Ar}OMe), 133.7, 129.1, 127.8 (3 × C_{Ar}), 127.8 (HC_{Ar}), 126.6 (C_{Ar}), 114.9, 113.1, 112.7 (3 × HC_{Ar}), 99.2 ($HC(OR)_2$), 65.1 (CH_2OPiv), 64.6 (OCH_2CH_2O), 61.7, 56.1, 55.7, 55.6 (4 × OCH_3), 44.5 (CH_2C=O), 38.6 ($C(CH_3)_3$), 33.5 (CH_2CH_2OPiv), 27.2 ($(CH_3)_3$); **IR** ν_{max} (neat)/cm^{-1} 2965, 1721, 1684, 1570, 1518, 1494, 1456, 1391, 1349, 1266, 1199, 1157, 1125, 1061, 1043; **HRMS** (ESI$^+$) $C_{28}H_{36}NaO_9$ requires 539.2252, found [M+Na$^+$] 539.2250 (+0.1 ppm).

Palmatine chloride

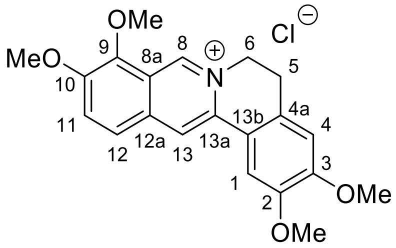

To a microwave vial containing ketone **10** (163 mg, 0.316 mmol) was added a 1 M solution of NH_4Cl in 3:1 EtOH/H_2O (3.2 mL) and the resulting mixture was stirred at 90 °C for 18 h. The temperature was then increased to 110 °C and the reaction was stirred for a further 48 h. The reaction mixture was concentrated *in vacuo* and the residue redissolved in H_2O (10 mL). A 20% w/w aqueous solution of NaOH (10 mL) was added and the solution was stirred for 5 min then extracted with CH_2Cl_2 (5 × 15 mL). The combined organic extracts were poured into a separating funnel containing H_2O (20 mL) before 1 M aqueous HCl (20 mL) was added to regenerate berberine chloride. The resulting mixture was washed with H_2O (3 × 20 mL) and the aqueous washes were combined and concentrated *in vacuo*. The solid residue was triturated with EtOAc (5 × 10 mL) to afford palmatine chloride as an orange-yellow solid (108 mg, 0.278 mmol, 88%).

M.p. 203-206 °C (lit. 206-208 °C); **^{1}H NMR** (400 MHz, DMSO-d$_6$) δ_H 9.91 (1 H, s, $HC(8)$), 9.10 (1 H, s, $HC(13)$), 8.21 (1 H, d, J = 9.2, $HC(11)$), 8.04 (1 H, d, J = 9.1, $HC(12)$), 7.73 (1 H, s, $HC(1)$), 7.09 (1 H, s, $HC(4)$), 4.96 (2 H, t, J = 6.1, C(6)H_2), 4.10 (3 H, s, C(9)OCH_3), 4.07 (3 H, s, C(10)OCH_3), 3.94 (3 H, s, C(2)OCH_3), 3.87 (3 H, s, C(3)OCH_3), 3.23 (2 H, t, J = 6.2, C(5)H_2); **^{13}C NMR** (101 MHz, DMSO-d$_6$) δ_C 151.5 ($C(3)$), 150.2 ($C(10)$), 148.7

(*C*(2)), 145.5 (H*C*(8)), 143.6 (*C*(9)), 137.7 (*C*(13a)), 133.1 (*C*(12a)), 128.6 (*C*(4a)), 126.7 (H*C*(11)), 123.4 (H*C*(12)), 121.3 (*C*(8a)), 119.9 (H*C*(13)), 118.9 (*C*(13b)), 111.3 (H*C*(4)), 108.8 (H*C*(1)), 61.9 (C(9)OCH_3), 57.0 (C(10)OCH_3), 56.2 (C(2)OCH_3), 55.9 (C(3)OCH_3), 55.3 (*C*(6)H_2), 26.0 (*C*(5)H_2). Spectroscopic data were consistent with those previously reported.[10]

2-(2-(2-(1,3-Dioxolan-2-yl)-3,4-dimethoxyphenyl)propanoyl)-4,5-dimethoxyphenethyl pivalate (12)

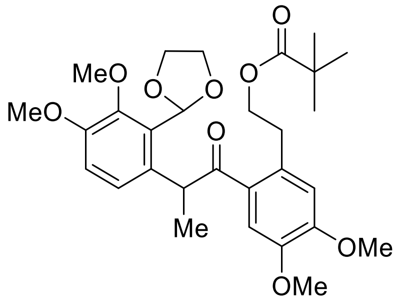

To a microwave vial fitted with a rubber septum was added aryl bromide **2** (203 mg, 0.702 mmol), Cs_2CO_3 (572 mg, 1.76 mmol) and $(Amphos)_2PdCl_2$ (24.9 mg, 0.0351 mmol). The septum was replaced with a microwave cap and the vessel was evacuated and backfilled with argon before a solution of ketone **10** (433 mg, 1.40 mmol) in anhydrous THF (3.5 mL) was added *via* syringe. The reaction mixture was stirred at 90 ºC for 18 h then cooled to room temperature. MeI (87.4 µL, 1.40 mmol) was added *via* syringe and the reaction was stirred at 70 °C for 24 h, after which it was cooled to room temperature and quenched with H_2O (5.0 mL). The resulting mixture was extracted with EtOAc (3 × 10 mL) and the combined organic extracts were dried over $MgSO_4$, filtered and concentrated *in vacuo*. Purification by flash column chromatography (SiO_2, 7:3 petrol/EtOAc) afforded ketone **12** as an off-white solid (284 mg, 0.535 mmol, 76%).

M.p. 136-138 °C; **^{1}H NMR** (400 MHz, $CDCl_3$) δ_H 7.48 (1 H, s, HC_{Ar}), 6.79 (1 H, d, $J = 8.7$, HC_{Ar}), 6.70 (1 H d, $J = 8.7$, HC_{Ar}), 6.70 (1 H, s, HC_{Ar}), 6.34 (1 H, s, $HC(OR)_2$), 5.02 (1 H, q, $J = 6.7$ $HC(CH_3)$), 4.38 (2 H, t, $J = 6.7$, CH_2OPiv), 4.19-4.14 (2 H, m, $OCH_aH_bCH_aH_bO$), 4.08-4.03 (2 H, m, $OCH_aH_bCH_aH_bO$), 3.87 (3 H, s, OCH_3), 3.81 (3 H, s, OCH_3), 3.80 (3 H, s, OCH_3), 3.74 (3 H, s, OCH_3), 3.31-3.17 (2 H, m, $CH_aH_bCH_2$OPiv), 1.49 (3 H, d, $J = 6.7$, $HC(CH_3)$), 1.18 (9 H, s, $(CH_3)_3$); **^{13}C NMR** (101 MHz, $CDCl_3$) δ_C 202.7 (*C*=O), 178.6 (RO*C*=O), 151.0, 150.7, 149.4, 146.7 (4 × C_{Ar}OMe), 135.7, 134.0, 128.6, 125.7 (4 × C_{Ar}), 124.0, 114.7, 114.2, 113.6 (4 × HC_{Ar}), 99.7 ($HC(OR)_2$), 65.2 (CH_2OPiv), 64.9, 64.5 (OCH_2CH_2O), 61.8, 56.4, 55.7, 55.6 (4 × OCH_3), 45.0 ($HC(CH_3)$), 38.7 ($C(CH_3)_3$), 34.2 (CH_2CH_2OPiv), 27.3 ($(CH_3)_3$), 19.8 ($HC(CH_3)$); **IR** ν_{max} (neat)/cm^{-1} 2970, 1722, 1671, 1568, 1492, 1456, 1391, 1349, 1267, 1205, 1156, 1137, 1056, 1030; **HRMS** (ESI$^+$) $C_{29}H_{38}NaO_9$ requires 553.2408, found [M+Na$^+$] 553.2403 (+0.6 ppm).

2-(7,8-Dimethoxy-4-methylisoquinolin-3-yl)-4,5-dimethoxyphenethyl pivalate (13)

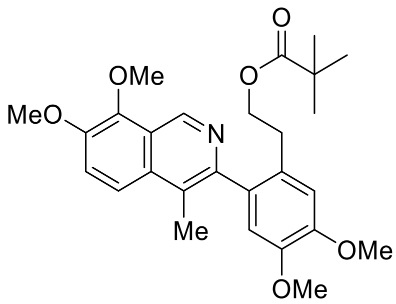

To a microwave vial containing ketone **12** (108 mg, 0.204 mmol) was added a 1 M solution of NH_4Cl in 3:1 $EtOH/H_2O$ (2.0 mL) and the resulting mixture was stirred at 90 °C for 24 h. After cooling to room temperature, NH_4HCO_3 (323 mg, 4.08 mmol) was added and the reaction was stirred at 90 °C for 18 h. After cooling to room temperature, the reaction mixture was diluted with water (2 mL) and extracted with EtOAc (3 × 5 mL). The combined organic extracts were dried over $MgSO_4$, filtered and concentrated *in vacuo*. Purification by flash column chromatography (SiO_2, 7:3petrol/EtOAc) afforded isoquinoline **13** as a yellow oil (89.3 mg, 0.191 mmol, 94%).

^{1}H NMR (400 MHz, $CDCl_3$) δ_H 9.47 (1 H, s, *H*C(1)), 7.77 (1 H, d, *J* = 9.2, *H*C(5)), 7.56 (1 H, d, *J* = 9.2, *H*C(6)), 6.90 (1 H, s, HC_{Ar}), 6.76 (1 H, s, HC_{Ar}), 4.10 (3 H, s, OCH_3), 4.06-4.04 (2 H, m, CH_2OPiv), 4.04 (3 H, s, OCH_3), 3.93 (3 H, s, OCH_3), 3.84 (3 H, s, OCH_3), 2.88-2.77 (1 H, m, $CH_aH_bCH_2$OPiv), 2.68-2.59 (1 H, m, $CH_aH_bCH_2$OPiv), 2.42 (3 H, s, CH_3), 1.11 (9 H, s, $(CH_3)_3$; **^{13}C NMR** (101 MHz, $CDCl_3$) δ_C 178.4 (*C*=O), 149.7, 148.5, 148.3, 147.3 (4 × C_{Ar}OMe), 144.8 (H*C*(1)), 144.1 (*C*(3)), 133.3, 131.6, 128.6, 124.5, 123.0 (5 × C_{Ar}), 119.8 (H*C*(5)), 119.6 (H*C*(6)), 113.0 (2 × HC_{Ar}), 64.4 (CH_2OPiv), 61.7, 56.9, 55.9, 55.9 (4 × OCH_3), 38.5 ($C(CH_3)_3$), 32.0 (CH_2CH_2OPiv), 27.1 ($(CH_3)_3$), 15.1 (CH_3); **IR** ν_{max} (neat)/cm^{-1} 2959, 1723, 1569, 1517, 1462, 1374, 1263, 1207, 1158, 1079, 1024; **HRMS** (ESI$^+$) $C_{27}H_{34}NO_6$ requires 468.2381, found [M+H$^+$] 468.2385 (−0.4 ppm).

2-(1-Ethoxy-7,8-dimethoxy-4-methyl-1H-isochromen-3-yl)-4,5-dimethoxyphenethyl pivalate (S4)

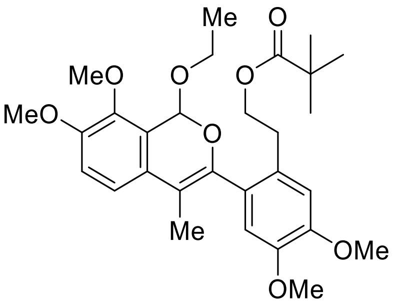

To a screw-capped tube containing ketone **12** (326 mg, 0.614 mmol) was added a 1 M solution of NH_4Cl in 3:1 $EtOH/H_2O$ (6.1 mL) and the reaction was stirred at 90 °C for 18 h. After cooling to room temperature, the reaction was diluted with H_2O (5 mL) and the resulting mixture was extracted with EtOAc (3 × 15 mL). The combined organic extracts were dried over $MgSO_4$, filtered and concentrated *in vacuo*. Purification by flash column chromatography (SiO_2, 8:2 petrol/EtOAc) afforded isochromene **S4** as an off-white solid (113 mg, 0.220 mmol, 36%).

M.p. 33-35 °C; **^{1}H NMR** (400 MHz, $CDCl_3$) δ_H 7.05 (1 H, d, *J* = 8.6, HC_{Ar}), 7.00 (1 H, d, *J* = 8.6, HC_{Ar}), 6.85 (1 H, s, HC_{Ar}), 6.77 (1 H, s, HC_{Ar}), 6.34 (1 H, s, *H*C(OR)$_2$), 4.22 (2 H, t, *J* = 6.7, CH_2OPiv), 4.05-4.00 (1 H, m, $OCH_aH_bCH_3$), 3.93 (3 H, s, OCH_3), 3.91 (3 H, s, OCH_3), 3.90 (3 H, s, OCH_3), 3.85 (3 H, s, OCH_3), 3.85-3.83 (1 H, m, $OCH_aH_bCH_3$), 3.06-2.87 (2 H, m, $CH_aH_bCH_2$OPiv), 1.86 (3 H, s, CH_3), 1.28 (3 H, t, *J* = 7.1, OCH_2CH_3), 1.16 (9 H, s, $(CH_3)_3$); **^{13}C NMR** (101 MHz, $CDCl_3$) δ_C 178.4 (*C*=O), 151.3, 148.8, 147.2, 145.1 (4 × C_{Ar}OMe), 144.7 (C=*C*OR), 129.8, 127.8 (2 × C_{Ar}), 125.1 (*C*=COR), 122.2 (C_{Ar}), 117.5, 113.2, 113.1, 112.4 (4 × HC_{Ar}), 107.2 (C_{Ar}), 93.6 (H*C*(OR)$_2$), 64.6 (CH_2OPiv), 63.8 (OCH_2CH_3), 60.9, 55.9, 55.8, 55.8 (4 × OCH_3), 38.6 ($C(CH_3)_3$), 31.5 (CH_2CH_2OPiv), 27.1 ($(CH_3)_3$), 15.1 (OCH_2CH_3), 13.2 (CH_3); **IR** ν_{max} (neat)/cm^{-1} 2972, 1725, 1514, 1498, 1462, 1354, 1278, 1248, 1228, 1155, 1058, 1026; **HRMS** (ESI$^+$) $C_{29}H_{38}NaO_8$ requires 537.2459, found [M+Na$^+$] 537.2460 (−0.3 ppm).

2-(2-(7,8-Dimethoxy-4-methylisoquinolin-3-yl)-4,5-dimethoxyphenyl)ethan-1-ol (S5)

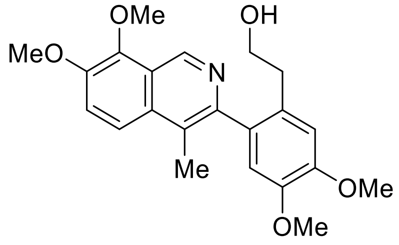

To a solution of isoquinoline **13** (53.0 mg, 0.113 mmol) in THF (1.1 mL) at −78 °C was added a 1 M solution of DIBAL-H in hexanes (283 µL, 0.283 mmol) dropwise. The reaction mixture was stirred at −78 °C for 4 h then warmed to room temperature and quenched by the dropwise addition of H_2O (5 mL). The resulting mixture was extracted with EtOAc (3 × 10 mL) and the combined organic extracts were dried over $MgSO_4$, filtered and concentrated *in vacuo*. Purification by flash column chromatography (SiO_2, 3:7 petrol/EtOAc) afforded alcohol **S5** as a colourless oil (40.0 mg, 0.104 mmol, 92%).

^{1}H NMR (400 MHz, $CDCl_3$) δ_H 9.44 (1 H, s, *H*C(1)), 7.77 (1 H, dd, *J* = 9.2, 0.8, *H*C(5)), 7.57 (1 H, d, *J* = 9.3, *H*C(6)), 6.91 (1 H, s, HC_{Ar}), 6.74 (1 H, s, HC_{Ar}), 4.08 (3 H, s, OCH_3), 4.03 (3 H, s, OCH_3), 3.95 (3 H, s, OCH_3), 3.93-3.89 (1 H, m, CH_aH_bOH), 3.83 (3 H, s, OCH_3), 3.81-3.76 (CH_aH_bOH), 2.69-2.66 (2 H, m, CH_2CH_2OH), 2.51 (3 H, s, CH_3); **^{13}C NMR** (101 MHz, $CDCl_3$) δ_C 149.0, 148.8, 148.6, 146.7, 144.1 (5 × C_{Ar}), 143.9 (H*C*(1)), 132.4, 132.1, 131.0, 125.1, 122.8 (5 × C_{Ar}), 120.0 (H*C*(6)), 119.7 (H*C*(5)), 113.6, 112.5 (2 × HC_{Ar}), 63.6 (CH_2OH), 61.7, 56.9, 56.0, 55.9 (4 × OCH_3), 35.2 (CH_2CH_2OH), 15.6 (CH_3); **IR** ν_{max} (neat)/cm^{-1} 3232, 2936, 2845, 1570, 1515, 1451, 1374, 1261, 1213, 1158, 1079, 1021; **HRMS** (ESI$^+$) $C_{22}H_{26}NO_5$ requires 384.1806, found [M+H$^+$] 384.1796 (−2.6 ppm).

Dehydrocorydaline chloride

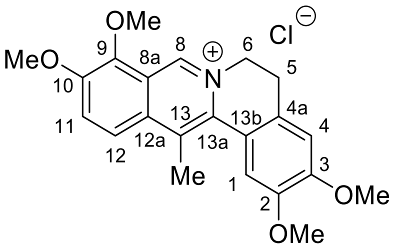

To a solution of alcohol **S5** (49.1 mg, 0.128 mmol) in anhydrous MeCN (5.0 mL) at 0 °C was added thionyl chloride (92.9 μL, 1.28 mmol) and the reaction mixture was warmed to room temperature then stirred at 50 °C for 3 h. The reaction mixture was then concentrated *in vacuo* and the solid residue redissolved in EtOH (5.0 mL) and stirred at 110 °C for 18 h. Concentration *in vacuo* afforded dehydrocorydaline as an orange-yellow solid (47.9 mg, 0.119 mmol, 93%).

M.p. 173-175 °C (lit. 162-163 °C); **^{1}H NMR** (500 MHz, DMSO-d$_6$) δ_H 9.91 (1 H, s, *H*C(8)), 8.22 (1 H, d, *J* = 9.3, *H*C(11)), 8.19 (1 H, d, *J* = 9.3, *H*C(12)), 7.39 (1 H, s, *H*C(1)), 7.18 (1 H, s, *H*C(4)), 4.85 (2 H, br. t, *J* = 5.6, C(6)H_2), 4.10 (3 H, s, C(9)OCH_3), 4.09 (3 H, s, C(10)OCH_3), 3.89 (3 H, s, C(3)OCH_3), 3.85 (3 H, s, C(2)OCH_3), 3.14 (2 H, br. t, *J* = 5.6, C(5)H_2), 2.98 (3 H, s, CH_3); **^{13}C NMR** (126 MHz, DMSO-d$_6$) δ_C 150.6 (*C*(3)), 150.2 (*C*(10)), 147.2 (*C*(2)), 144.0 (*C*(9)), 144.0 (H*C*(8)), 136.0 (*C*(13a)), 133.1 (*C*(12a)), 131.8 (*C*(4a)), 129.7 (*C*(13)), 125.9 (H*C*(11)), 121.3 (H*C*(12)), 120.7 (*C*(8a)), 119.1 (*C*(13b)), 114.3 (H*C*(1)), 110.9 (H*C*(4)), 62.0 (C(9)O*C*H$_3$), 57.0 (C(10)O*C*H$_3$), 56.8 (*C*(6)H$_2$), 56.2 (C(2)O*C*H$_3$), 55.9 (C(3)O*C*H$_3$), 26.8 (*C*(5)H$_2$), 17.7 (*C*H$_3$); **^{13}C NMR** (176 MHz, (CDCl$_3$) δ_C 151.3 (*C*(3)), 150.6 (*C*(10)), 147.7 (*C*(2)), 146.3 (*C*(9)), 146.3 (H*C*(8)), 136.5 (*C*(13a)), 133.8 (*C*(12a)), 132.2 (*C*(4a)), 128.8 (*C*(13)), 125.6 (H*C*(12)), 121.8 (*C*(8a)), 120.1 (H*C*(11)), 119.3 (*C*(13b)), 114.0 (H*C*(4)), 110.8 (H*C*(1)), 63.8 (*C*(6)H$_2$), 57.8 (C(3)O*C*H$_3$), 57.2 (C(9)O*C*H$_3$), 56.8 (C(10)O*C*H$_3$), 56.3 (C(2)O*C*H$_3$), 28.6 (*C*(5)H$_2$), 18.4 (C(13)*C*H$_3$). Spectroscopic data were consistent with those previously reported.[11,12]

2-(2-Bromo-5-fluorophenyl)-1,3-dioxolane (14)

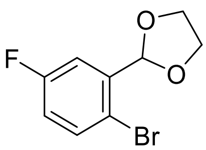

2-Bromo-5-fluorobenzaldehyde (2.07 g, 10.2 mmol) was subjected to **General Procedure 1**. Purification by flash column chromatography (SiO$_2$, 9:1 petrol/EtOAc) afforded acetal **14** as a colourless oil (2.49 g, 10.1 mmol, 99%).

^{1}H NMR (400 MHz, CDCl$_3$) δ_H 7.51 (1 H, dd, *J* = 8.7, 5.1, *H*C$_{Ar}$), 7.33 (1 H, dd, *J* = 9.2, 3.1, *H*C$_{Ar}$), 6.95 (1 H, m, *H*C$_{Ar}$), 6.04 (1 H, d, ^{5}J = 1.3, *H*C(OR)$_2$), 4.17-4.02 (4 H, m, OCH_2CH_2O); **^{13}C NMR** (101 MHz, CDCl$_3$) δ_C 161.9 (d, ^{1}J = 247.8, *C*$_{Ar}$F), 138.8 (d, ^{3}J = 6.3, *C*$_{Ar}$), 134.2 (d, ^{3}J = 8.0, H*C*$_{Ar}$), 117.6 (d, ^{2}J = 22.4, H*C*$_{Ar}$), 116.7 (d, ^{4}J = 3.2, *C*$_{Ar}$Br), 115.0 (d, ^{2}J = 24.9, H*C*$_{Ar}$), 65.4 (O*C*H$_2$*C*H$_2$O); **^{19}F{^{1}H} NMR** (377 MHz, CDCl$_3$) −113.9 (C$_{Ar}$*F*);

IR ν_{max} (neat)/cm^{-1} 2890, 1583, 1468, 1415, 1391, 1264, 1159, 1118, 1082, 1030; **HRMS** (FI$^+$) $C_9H_8{}^{[79]}BrFO_2$ requires 245.9692, found [M$^+$] 245.9693 (+0.5 ppm).

2-(6-(2-(2-(1,3-Dioxolan-2-yl)-4-fluorophenyl)acetyl)benzo[d][1,3]dioxol-5-yl)ethyl pivalate (15)

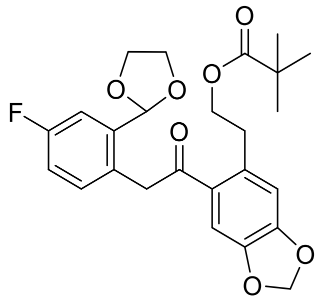

To a microwave vial fitted with a rubber septum was added aryl bromide **14** (330 mg, 1.34 mmol), ketone **4** (781 mg, 2.67 mmol), Cs_2CO_3 (1.09 g, 3.34 mmol) and $(Amphos)_2PdCl_2$ (47.3 mg, 0.0668 mmol). The septum was replaced with a microwave cap and the vessel was evacuated and backfilled with argon before the solids were dissolved in anhydrous THF (6.7 mL). The reaction mixture was stirred at 90 ºC for 24 h then cooled to room temperature. The crimped cap was removed and replaced with a rubber septum and the flask was purged with argon whilst additional $(Amphos)_2PdCl_2$ (47.3 mg, 0.0668 mmol) was added. A new microwave cap was fitted and the vessel purged with argon for 5 mins before stirring at 90 °C for a further 24 h. After cooling to room temperature, the reaction was quenched with H_2O (5 mL). The resulting mixture was extracted with EtOAc (3 × 20 mL) and the combined organic extracts were dried over $MgSO_4$, filtered and concentrated *in vacuo*. Purification by flash column chromatography (SiO_2, 75:25 petrol/Et_2O) afforded ketone **15** as an off-white solid (456 mg, 0.995 mmol, 74%).

M.p. 78-82 °C; **^{1}H NMR** (400 MHz, $CDCl_3$) δ_H 7.36 (1 H, s, HC_{Ar}), 7.32 (1 H, dd, J = 9.7, 2.8, HC_{Ar}), 7.16-7.13 (1 H, m, HC_{Ar}), 7.04 (1 H, td, J = 8.2, 2.8, HC_{Ar}), 6.78 (1 H, s, HC_{Ar}), 6.04 (2 H, s, OCH_2O), 5.84 (1 H, s $HC(OR)_2$), 4.32 (2 H, s, CH_2C=O), 4.25 (2 H, t, J = 6.6, CH_2OPiv), 4.02-3.99 (2 H, m, $OCH_aH_bCH_aH_bO$), 3.97-3.94 (2 H, m, $OCH_aH_bCH_aH_bO$), 3.11 (2 H, t, J = 6.6, CH_2CH_2OPiv), 1.16 (9 H, s, $(CH_3)_3$); **^{13}C NMR** (101 MHz, $CDCl_3$) δ_C 198.2 (C=O), 178.4 (ROC=O), 161.8 (d, ^1J = 246.3, C_{Ar}F), 150.0 (C_{Ar}OR), 146.1 (C_{Ar}OR), 138.3 (d, ^3J = 6.3, C_{Ar}), 135.4 (C_{Ar}), 133.2 (d, ^3J = 7.9, HC_{Ar}), 130.6 (C_{Ar}), 129.1 (d, ^4J = 3.2, C_{Ar}), 115.7 (d, ^2J = 20.7, HC_{Ar}), 113.8 (d, ^2J = 22.3, HC_{Ar}), 112.4 (HC_{Ar}), 109.1 (HC_{Ar}), 101.8 (OCH$_2$O), 101.6 (HC(OR)$_2$, 65.0 (OCH$_2$$CH_2O, CH_2$OPiv), 44.3 (CH$_2$C=O), 38.7 (C(CH$_3$)$_3$), 33.7 (CH$_2CH_2$OPiv), 27.2 (CH$_3$)$_3$; **^{19}F{^{1}H} NMR** (377 MHz, $CDCl_3$) −115.1 ($C_{Ar}F$); **IR** ν_{max} (neat)/cm^{-1} 2976, 2900, 1721, 1686, 1611, 1489, 1375, 1266, 1241, 1156, 1078, 1036; **HRMS** (ESI$^+$) $C_{25}H_{27}FNaO_7$ requires 481.1633, found [M+Na$^+$] 481.1620 (−2.8 ppm).

2-(6-(7-Fluoroisoquinolin-3-yl)benzo[d][1,3]dioxol-5-yl)ethyl pivalate (S6)

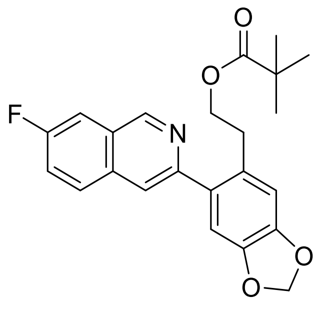

To a microwave vial containing ketone **15** (55.0 mg, 0.120 mmol) was added a 1 M solution of NH_4Cl in 3:1 EtOH/H_2O (1.2 mL) and the resulting mixture was stirred at 60 °C for 4 h. After cooling to room temperature, NH_4HCO_3 (190 mg, 2.40 mmol) was added and the reaction was stirred at 90 °C for 18 h. After cooling to room temperature, the reaction mixture was diluted with water (2 mL) and extracted with EtOAc (3 × 5 mL). The combined organic extracts were dried over $MgSO_4$, filtered and concentrated *in vacuo*. Purification by flash column chromatography (SiO_2, 9:1 petrol/Et_2O) afforded isoquinoline **S6** as a colourless oil (30.4 mg, 0.0769 mmol, 64%).

^{1}H NMR (400 MHz, $CDCl_3$) δ_H 9.26 (1 H, s, *H*C(1)), 7.87 (1 H, dd, *J* = 9.0, 5.2, *H*C(5)), 7.74 (1 H, s, *H*C(4)), 7.61 (1 H, dd, *J* = 8.7, 2.5, *H*C(8)), 7.50 (1 H, td, *J* = 8.7, 2.6, *H*C(6)), 6.95 (1 H, s, HC_{Ar}), 6.87 (1 H, s, HC_{Ar}), 6.00 (2 H, s, OCH_2O), 4.19 (2 H, t, *J* = 6.9, CH_2OPiv), 3.01 (2 H, t, *J* = 6.9, CH_2CH_2OPiv), 1.14 (9 H, s, $(CH_3)_3$); **^{13}C NMR** (101 MHz, $CDCl_3$) δ_C 178.4 (*C*=O), 160.8 (d, ^1J = 249.4, *C*(7)), 152.8 (d, ^6J = 3.2, *C*(3)), 151.0 (d, ^4J = 5.6, H*C*(1)), 147.5, 146.2 (2 × C_{Ar}OR), 134.0, 133.3 (2 × C_{Ar}), 130.1 (*C*(4a)), 129.4 (d, ^3J = 8.0, H*C*(5)), 127.7 (^3J = 8.4, *C*(8a)), 121.2 (d, ^2J = 25.3, H*C*(6)), 120.0 (H*C*(4)), 110.5 (d, ^2J = 19.8, H*C*(8)), 110.5, 110.5 (2 × HC_{Ar}), 101.2 (OCH_2O), 64.9 (CH_2OPiv), 38.6 ($C(CH_3)_3$), 32.3 (CH_2CH_2OPiv), 27.1 ($(CH_3)_3$); **^{19}F{^{1}H} NMR** (377 MHz, $CDCl_3$) −111.2 (C(7)*F*); **IR** ν_{max} (neat)/cm^{-1} 2972, 1721, 1619, 1494, 1459, 1373, 1283, 1249, 1230, 1150, 1037; **HRMS** (ESI$^+$) $C_{23}H_{23}FNO_4$ requires 396.1606, found [M+H$^+$] 396.1597 (−2.1 ppm).

2-(6-(7-Fluoroisoquinolin-3-yl)benzo[d][1,3]dioxol-5-yl)ethan-1-ol (S7)

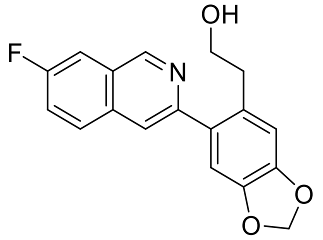

To a solution of isoquinoline **S6** (43.1 mg, 0.109 mmol) in THF (1.1 mL) at −78 °C was added a 1 M solution of DIBAL-H in hexanes (272 µL, 0.272 mmol) dropwise. The reaction mixture was stirred at −78 °C for 5 h then warmed to room temperature and quenched by the dropwise addition of H_2O (5 mL). The resulting mixture was extracted with EtOAc (3 × 10 mL) and the combined organic extracts were dried over $MgSO_4$, filtered and concentrated *in vacuo*. Purification by flash column chromatography (SiO_2, 7:3 petrol/EtOAc) afforded alcohol **S7** as a white solid (32.1 mg, 0.103 mmol, 95%).

M.p. 115-118 °C; **^{1}H NMR** (400 MHz, $CDCl_3$) δ_H 9.20 (1 H, s, *H*C(1)), 7.89 (1 H, dd, *J* = 9.0, 5.2, *H*C(5)), 7.82 (1 H, s, *H*C(4)), 7.63 (1 H, dd, *J* = 8.6, 2.4, *H*C(8)), 7.53 (1 H, td, *J* = 8.7, 2.5, *H*C(6)), 6.92 (1 H, s, HC_{Ar}), 6.88 (1 H, s, HC_{Ar}), 6.55 (1 H, br. s, O*H*), 6.00 (2 H, s, OCH_2O), 3.98 (2 H, t, *J* = 5.7, CH_2OH), 2.82 (2 H, t, *J* = 5.7, CH_2CH_2OH); **^{13}C NMR** (101 MHz, $CDCl_3$) δ_C 160.9 (d, ^{1}J = 249.4, *C*(7)), 152.0 (*C*(3)), 149.6 (d, ^{4}J = 4.8, H*C*(1)), 148.3, 146.2 (2 × $C_{Ar}OR$), 133.9, 133.1, 132.6 (3 × C_{Ar}), 129.4 (d, ^{3}J = 8.7, H*C*(5)), 127.6 (d, ^{3}J = 8.6, C_{Ar}), 121.8 (d, ^{2}J = 25.4, H*C*(6)), 120.5 (H*C*(4)), 110.7 (d, ^{2}J = 20.7, H*C*(8)), 110.0 (2 × HC_{Ar}), 101.3 (OCH_2O), 63.8 (CH_2OH), 35.2 (CH_2CH_2OH); **^{19}F{^{1}H} NMR** (377 MHz, $CDCl_3$) −110.4 (C(7)*F*); **IR** ν_{max} (neat)/cm^{-1} 3222, 2853, 1619, 1494, 1461, 1376, 1248, 1232, 1141, 1042; **HRMS** (ESI$^+$) $C_{18}H_{15}FNO_3$ requires 312.1031, found [M+H$^+$] 312.1022 (−2.6 ppm).

10-Fluoro-5,6-dihydro-[1,3]dioxolo[4,5-g]isoquinolino[3,2-a]isoquinolin-7-ium chloride (16)

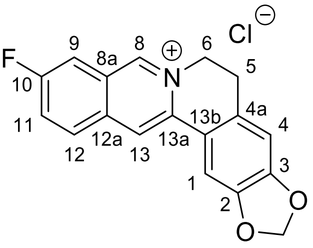

To a solution of alcohol **S6** (27.4 mg, 0.0880 mmol) in anhydrous MeCN (3.0 mL) at 0 °C was added thionyl chloride (63.8 µL, 0.880 mmol) and the reaction mixture was warmed to room temperature and stirred at 50 °C for 2 h. The resulting suspension was concentrated *in vacuo* and the solid triturated with Et_2O (5 × 5 mL) to afford protoberberine **16** as a yellow solid (23.7 mg, 0.0719 mmol, 82%).

M.p. decomposed 299 °C; **^{1}H NMR** (400 MHz, DMSO-d$_6$) δ_H 10.12 (1 H, s, *H*C(8)), 9.14 (1 H, s, *H*C(13)), 8.36-8.33 (1 H, m, *H*C(12)), 8.28 (1 H, dd, *J* = 8.7, 2.0, *H*C(9)), 8.14 (1 H, td, *J* = 8.8, 2.3, *H*C(11)), 7.82 (1 H, s, *H*C(1)), 7.11 (1 H, s, *H*C(4)), 6.18 (2 H, s, OCH_2O), 4.90 (2 H, t, *J* = 6.0, C(6)H_2), 3.23 (2 H, t, *J* = 6.0, C(5)H_2); **^{13}C NMR** (101 MHz, DMSO-d$_6$) δ_C 161.5 (d, ^{1}J = 250.8, *C*(10)), 150.2 (*C*(2)), 149.6 (d, ^{4}J = 5.1, H*C*(8)), 147.7 (*C*(3)), 139.5 (*C*(13a)), 136.0 (*C*(12a)), 131.1 (*C*(13b)), 130.9 (d, ^{3}J = 9.5, H*C*(12)), 127.3 (d, ^{2}J = 26.8, H*C*(11), 126.5 (d, ^{3}J = 11.2, *C*(8a)), 120.7 (H*C*(13)), 120.2 (*C*(4a)), 112.85 (d, ^{2}J = 23.7, H*C*(9)), 108.5 (H*C*(4)), 105.7 (H*C*(1)), 102.2 (OCH_2O), 55.4 (*C*(6)H_2), 26.2 (*C*(5)H_2); **^{19}F{^{1}H} NMR** (377 MHz, $CDCl_3$) −106.5 (C(10)*F*); **IR** ν_{max} (neat)/cm^{-1} 3357, 2999, 2921, 1606, 1497, 1476, 1371, 1253, 1223, 1163, 1094, 1034; **HRMS** (ESI$^+$) $C_{18}H_{13}NO_2F$ requires 294.0925, found [M$^+$] 294.0918 (−2.2 ppm).

NMR Comparison Tables

Berberine chloride

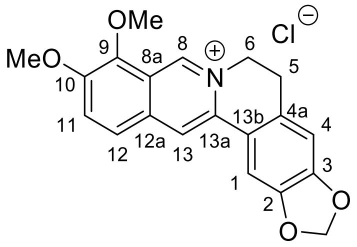

	δ_H			δ_C		
Atom	Lit.[2] (DMSO-d_6)	Observed (DMSO-d_6)	Δ	Lit.[2] (DMSO-d_6)	Observed (DMSO-d_6)	Δ
1	7.79 s	7.79 s	0.00	105.4	105.4	0.0
2	-	-	-	147.6	147.6	0.0
3	-	-	-	149.8	149.8	0.0
4	7.09 s	7.08 s	−0.01	108.4	108.4	0.0
4a	-	-	-	130.6	130.6	0.0
5	3.22 t (6.2)	3.21 t (6.2)	−0.01	26.4	26.3	−0.1
6	4.95 t (6.3)	4.95 t (6.3)	0.00	55.2	55.1	−0.1
8	9.91 s	9.90 s	−0.01	145.4	145.4	0.0
8a	-	-	-	121.4	121.4	0.0
9	-	-	-	143.6	143.6	0.0
10	-	-	-	150.4	150.3	−0.1
11	8.20 d (9.1)	8.19 d (9.1)	−0.01	126.7	126.7	0.0
12	8.01 d (9.1)	8.01 d (9.1)	0.00	123.5	123.5	0.0
12a	-	-	-	132.9	133.0	0.1
13	8.96 s	8.97 s	0.01	120.2	120.2	0.0
13a	-	-	-	137.4	137.4	0.0
13b	-	-	-	120.4	120.4	0.0
OCH_2O	6.17 s	6.17 s	0.00	102.1	102.0	−0.1
9-OCH_3	4.10 s	4.10 s	0.00	62.0	61.9	−0.1
10-OCH_3	4.07 s	4.07 s	0.00	57.1	57.1	0.0

Pseudocoptisine chloride

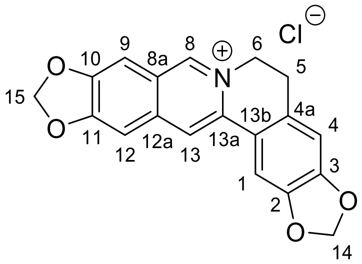

	δ_H					δ_C	
Atom	Lit.[13*] ($CDCl_3$)	Lit.[14†] (CD_3OD)	Observed (CD_3OD)	Δ		Lit.[14] (CD_3OD)	Observed (CD_3OD)
1	7.84 s	7.85 s	7.64 s	−0.19		150.8	158.5
2	-	-	-	-		148.6	153.3
3	-	-	-	-		147.9	152.5
4	6.91 s	6.94 s	6.96 s	0.02		147.6	150.1
4a	-	-	-	-		144.5	146.7
5	3.24 t (6.0)	3.24 m	3.23 t (6.4)	−0.01		144.0	141.3
6	4.88 t (6.0)	4.88 t (6.7)	4.78 t (6.5)	−0.10		133.1	141.0
8	9.72 s	9.72 s	9.31 s	−0.41		130.6	132.2
8a	-	-	-	-		121.9	125.8
9	7.84 s	7.85 s	7.55 s	−0.30		121.3	121.9
10	-	-	-	-		121.1	120.4
11	-	-	-	-		120.7	109.6
12	7.59 s	7.63 s	7.51 s	−0.12		112.4	106.7
12a	-	-	-	-		108.2	105.6
13	8.67 s	8.71 s	8.55 s	−0.16		105.2	104.9
13a	-	-	-	-		104.6	104.2
13b	-	-	-	-		102.5	103.9
14	6.10 s	6.09 s	6.11 s	0.02		56.0	56.4
15	6.91 s	6.45 s	6.35 s	−0.10		27.0	28.3

*Due to poor solubility of our sample (yellow solid), we were unable to obtain NMR data in $CDCl_3$. Lit.: brown powder. Low solubility in $CHCl_3$ has been reported previously.[5]

†Counterion not specified.

2-(6-([1,3]Dioxolo[4,5-g]isoquinolin-7-yl)benzo[d][1,3]dioxol-5-yl)ethan-1-ol (S2)

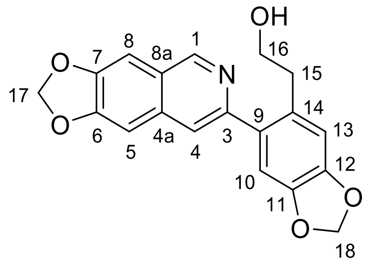

	δ_H			δ_C		
Atom	Lit.[6] ($CDCl_3$)	Observed ($CDCl_3$)	Δ	Lit.[6] ($CDCl_3$)	Observed ($CDCl_3$)	Δ
1	9.00 s	8.95 s	−0.05	147.7	147.9	0.2
3	-	-	-	151.0	151.4	0.4
4	7.63 s	7.63 s	0.00	120.5	120.3	−0.2
4a	-	-	-	124.4	124.4	0.0
5	7.10 s	7.10 s	0.00	102.6	102.5	−0.1
6	-	-	-	148.8	148.6	−0.2
7	-	-	-	151.9	151.6	−0.3
8	7.22 s	7.22 s	0.00	103.2	103.0	−0.2
8a	-	-	-	135.8	135.5	−0.3
10	not reported	6.91 s	-	110.0	109.9	−0.1
11	-	-	-	146.2	146.1	−0.1
12	-	-	-	148.2	148.1	−0.1
13	6.85 s	6.87 s	−0.02	110.1	110.0	−0.1
15	5.08 t (6.0)*	2.82 t (5.6)	-	35.3	35.2	−0.1
16	3.97 t (6.0)	3.98 t (5.6)	0.01	63.8	63.8	0.0
17	6.12 s	6.13 s	0.01	101.9	101.8	−0.1
18	5.98 s	6.00 s	0.02	101.3	101.2	−0.1

* δ assumed incorrect.

Tetrahydropseudocoptisine

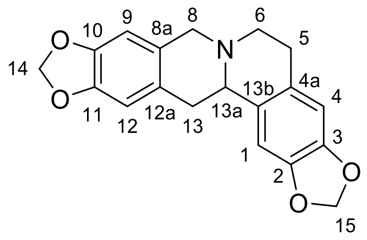

	δ_H		
Atom	Lit.[8] ($CDCl_3$)	Observed ($CDCl_3$)	Δ
1	6.72 s	6.73 s	0.01
2	-	-	-
3	-	-	-
4	6.58	6.59 s	0.01
4a	-	-	-
13(H_a)	3.19-3.10 m	3.17 dd (16.1, 3.9)	−0.03*
5(H_a)		3.14-3.07 m	
6(H_a)			
13(H_b)	2.83-2.59 m	2.80 dd (15.7, 11.4)	−0.04*
5(H_b)		2.71-2.54 m	
6(H_b)			
8(H_a)	3.92-3.87 m	3.91 d (14.6)	0.01*
8(H_b)	3.65-3.51 m	3.64 d (14.6)	0.01*
13a		3.54 dd (11.2, 3.6)	
8a	-	-	-
9	6.53 s	6.55 s	0.02
10	-	-	-
11	-	-	-
12	6.60 s	6.62 s	0.02
12a	-	-	-
13b	-	-	-
14	5.90 s	5.91 s	0.01
15	5.91 s	5.92 s	0.01

* With respect to midpoint of multiplets.

	δ_C		
Atom	Lit.[9] ($CDCl_3$)	Observed ($CDCl_3$)	Δ
1	105.5	105.4	−0.1
2	146.3	146.1	−0.2
3	146.3	146.1	−0.2
10	146.2	145.9	−0.3
11	146.0	145.8	−0.2
4	108.5	108.4	−0.1
4a	130.9	130.7	−0.2
13b	127.8	127.7	−0.1
5	29.6	29.5	−0.1
6	51.2	51.2	0.0
8	58.6	58.5	−0.1
8a	127.3	127.2	−0.1
9	106.1	106.0	−0.1
12	108.5	108.4	−0.1
12a	127.3	127.2	−0.1
13	37.0	36.9	−0.1
13a	59.9	59.8	−0.1
14	100.7	100.6	−0.1
15	100.8	100.8	0.0

Palmatine chloride

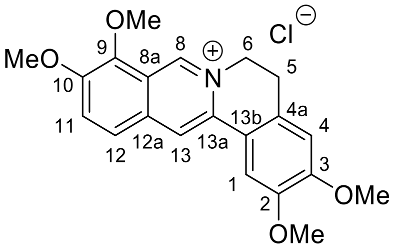

	δ_H			δ_C		
Atom	Lit.[10] (DMSO-d_6)	Observed (DMSO-d_6)	Δ	Lit.[10] (DMSO-d_6)	Observed (DMSO-d_6)	Δ
1	7.72 s	7.73 s	0.01	109.2	108.8	−0.4
2	-	-	-	148.9	148.7	−0.2
3	-	-	-	151.7	151.5	−0.2
4	7.08 s	7.09 s	0.01	111.5	111.3	−0.2
4a	-	-	-	128.8	128.6	−0.2
5	3.22 t (6.2)	3.23 t (5.8)	0.01	26.1	26.0	−0.1
6	4.95 t (6.1)	4.96 t (5.8)	0.01	55.5	55.3	−0.2
8	9.88 s	9.91 s	0.03	145.6	145.5	−0.1
8a	-	-	-	121.5	121.3	−0.2
9	-	-	-	143.8	143.6	−0.2
10	-	-	-	150.4	150.2	−0.2
11	8.20 d (9.2)	8.21 d (9.0)	0.01	127.0	126.7	−0.3
12	8.03 d (9.1)	8.04 d (9.0)	0.01	123.6	123.4	−0.2
12a	-	-	-	133.3	133.1	−0.2
13	9.07 s	9.10 s	0.03	120.1	119.9	−0.2
13a	-	-	-	137.9	137.7	−0.2
13b	-	-	-	119.1	118.9	−0.2
2-OCH_3	3.93 s	3.94 s	0.01	56.4	56.2	−0.2
3-OCH_3	3.86 s	3.87 s	0.01	56.0	55.9	−0.1
9-OCH_3	4.09 s	4.10 s	0.01	62.1	61.9	−0.2
10-OCH_3	4.06 s	4.07 s	0.01	57.2	57.0	−0.2

Dehydrocorydaline chloride

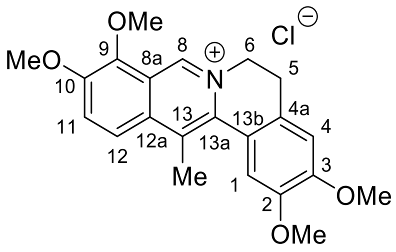

	δ_H			δ_C		
Atom	Lit.[11] (DMSO-d_6)	Observed (DMSO-d_6)	Δ	Lit.[12] ($CDCl_3$)	Observed ($CDCl_3$)	Δ
1	7.39 s	7.39 s	0.00	110.7	110.8	0.1
2	-	-	-	151.3	151.3	0.1
3	-	-	-	150.5	150.6	0.1
9	-	-	-	147.7	147.7	0.0
10	-	-	-	146.3	146.3	0.0
4a	-	-	-	136.3	136.5	0.2
8a	-	-	-	133.7	133.8	0.1
12a	-	-	-	132.2	132.2	0.0
13a	-	-	-	121.7	121.8	0.1
13b	-	-	-	119.2	119.3	0.1
4	7.18 s	7.18 s	0.00	113.9	114.0	0.1
5	3.16 t (5.4)	3.14 t (5.6)	−0.02	28.2	28.6	0.4
6	4.88 t (5.4)	4.85 t (5.6)	−0.03	63.2	63.8	0.6
8	9.93 s	9.91 s	−0.02	146.5	146.3	−0.2
11	8.20 s	8.22 d (9.3)	0.02	119.7	120.1	0.4
12	8.20 s	8.19 d (9.3)	−0.01	125.4	125.6	0.2
13	-	-	-	128.5	128.8	0.3
2-OCH_3	3.86 s	3.85 s	−0.01	56.2	56.3	0.1
3-OCH_3	3.90 s	3.89 s	−0.01	57.1	57.8	0.7
9-OCH_3	4.11 s	4.10 s	−0.01	56.9	57.2	0.3
10-OCH_3	4.10 s	4.09 s	−0.01	56.5	56.8	0.3
13-CH_3	2.99 s	2.98 s	−0.01	17.9	18.4	0.5

^{1}H NMR and ^{13}C NMR Spectra of Compounds

(2) 2-(6-Bromo-2,3-dimethoxyphenyl)-1,3-dioxolane ($CDCl_3$)

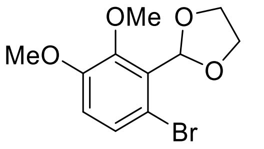

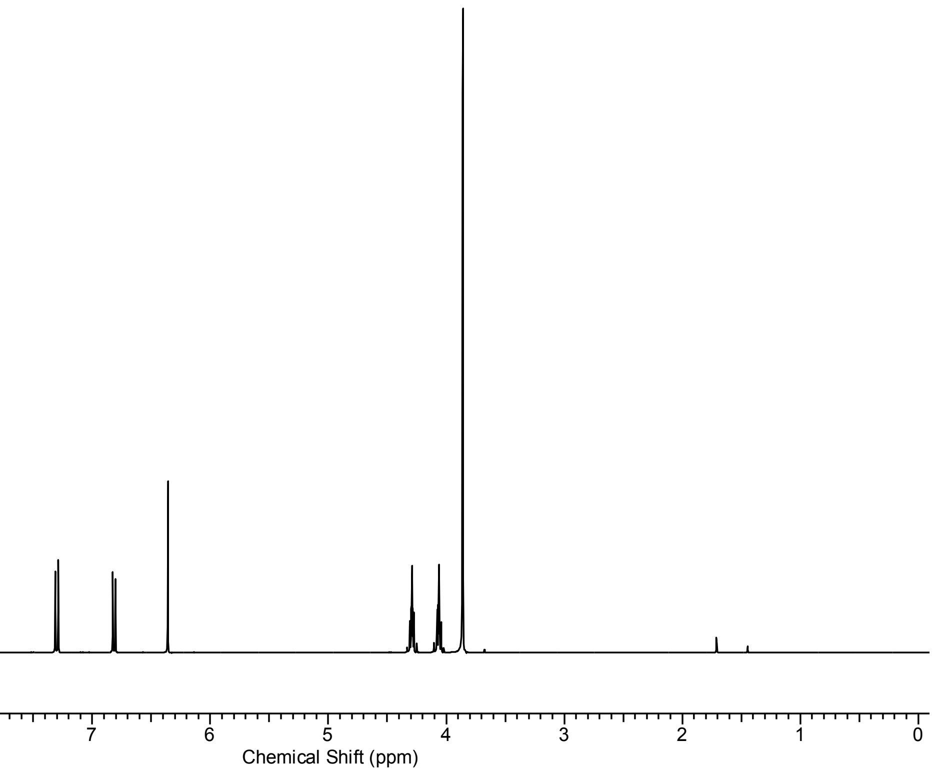

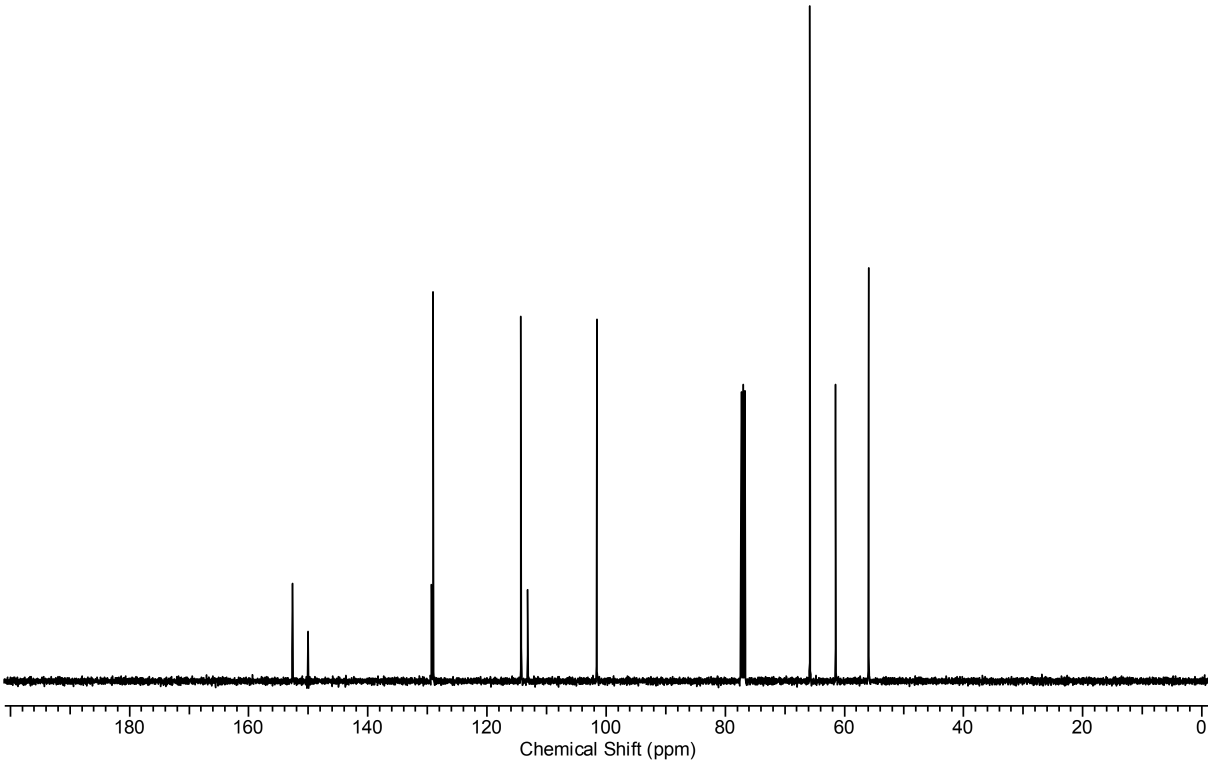

(S1) 2-(Benzo[d][1,3]dioxol-5-yl)ethyl pivalate ($CDCl_3$)

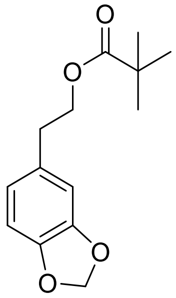

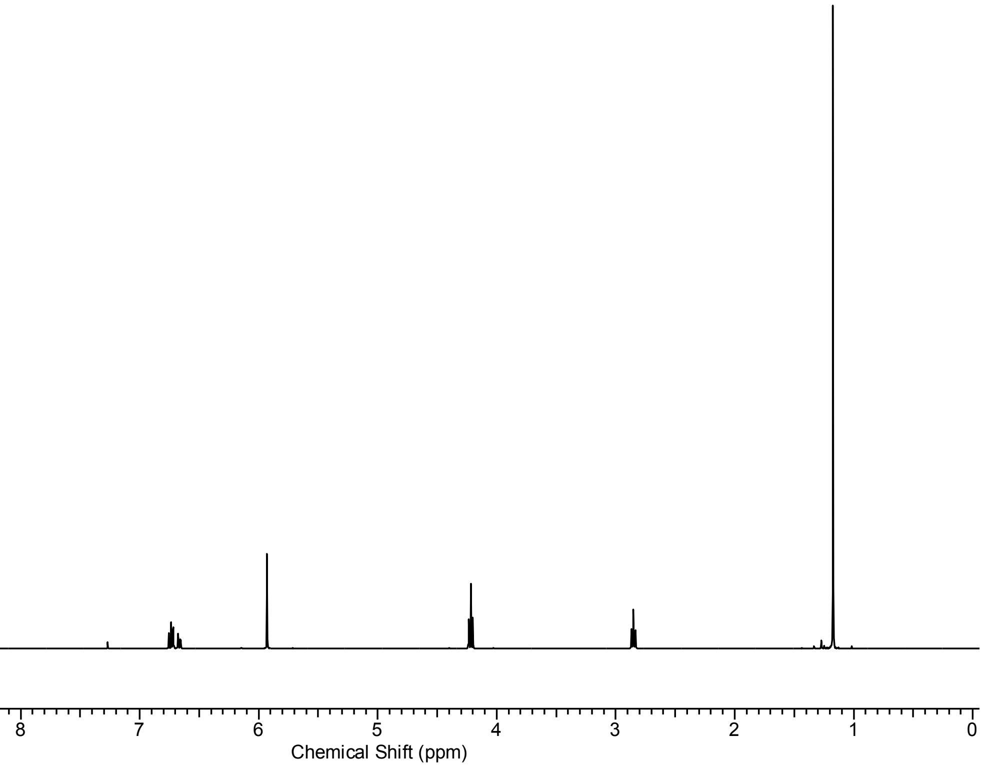

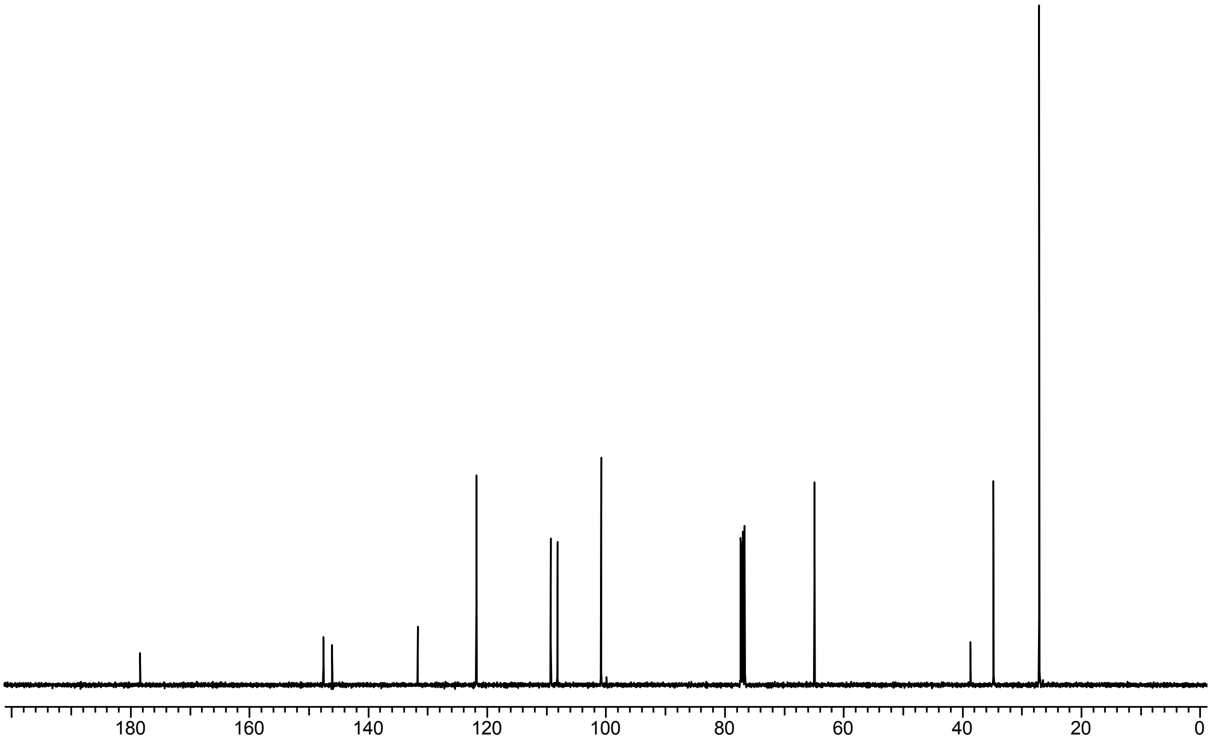

(4) 2-(6-Acetylbenzo[d][1,3]dioxol-5-yl)ethyl pivalate ($CDCl_3$)

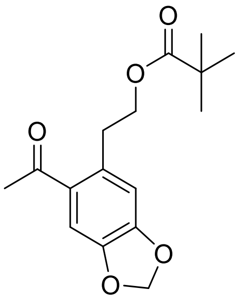

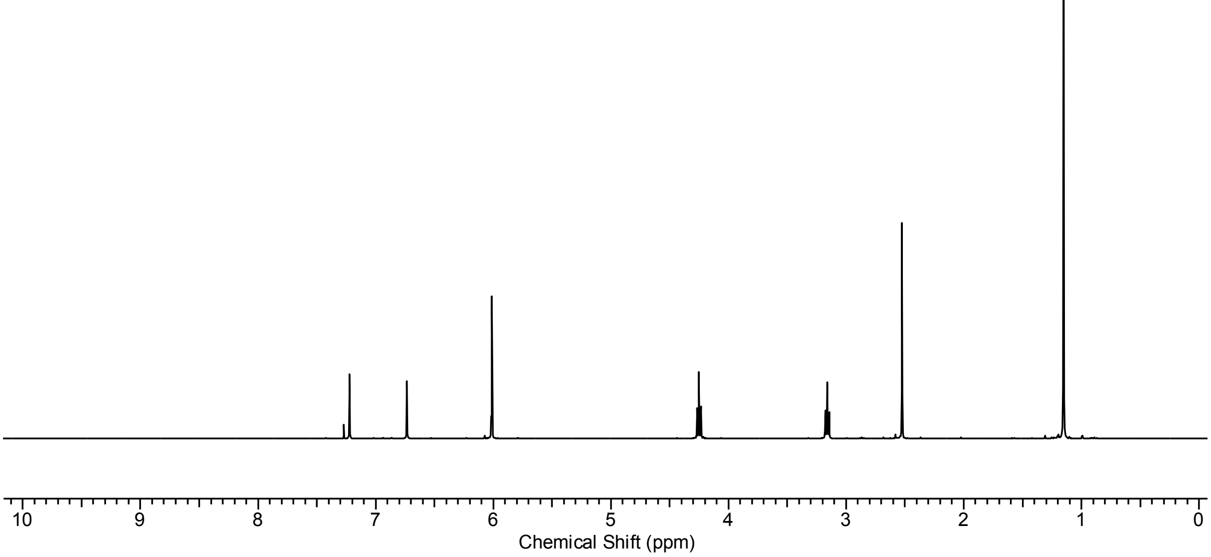

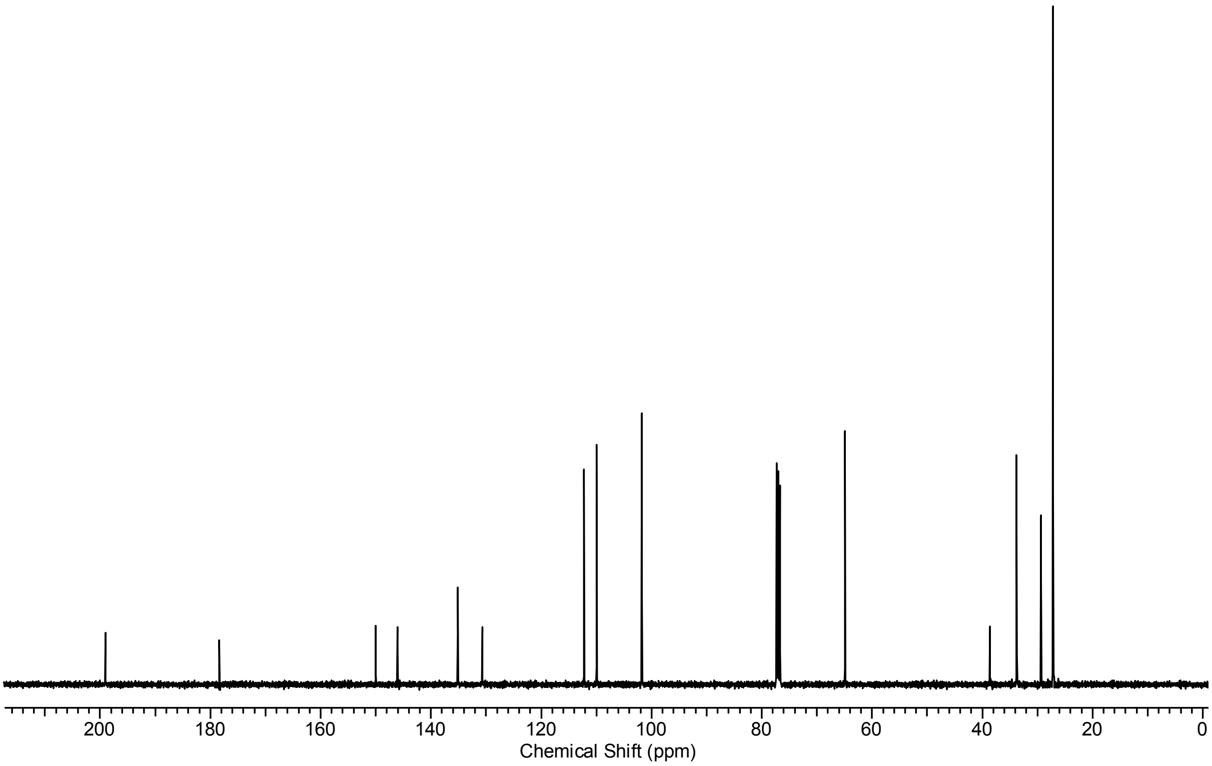

(5) 2-(6-(2-(2-(1,3-Dioxolan-2-yl)-3,4-dimethoxyphenyl)acetyl)benzo[d][1,3]dioxol-5-yl)ethyl pivalate ($CDCl_3$)

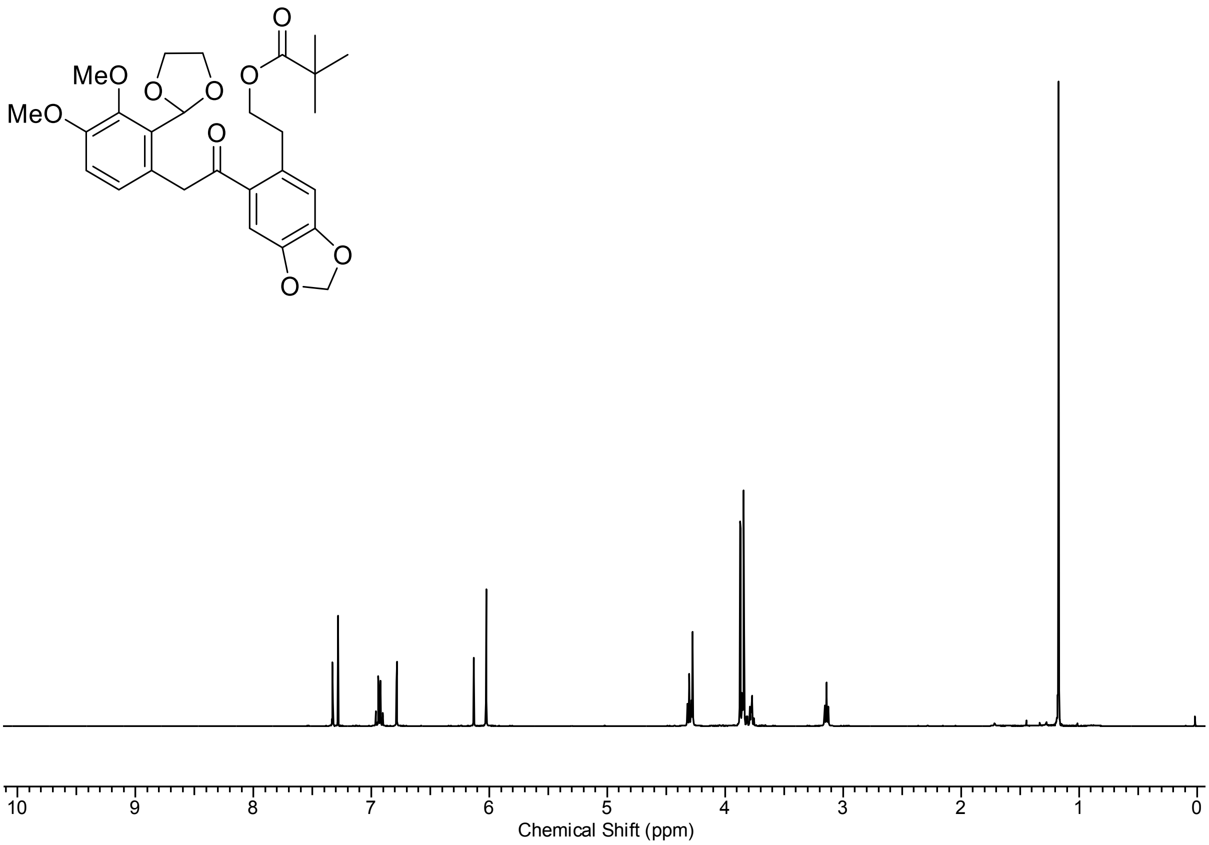

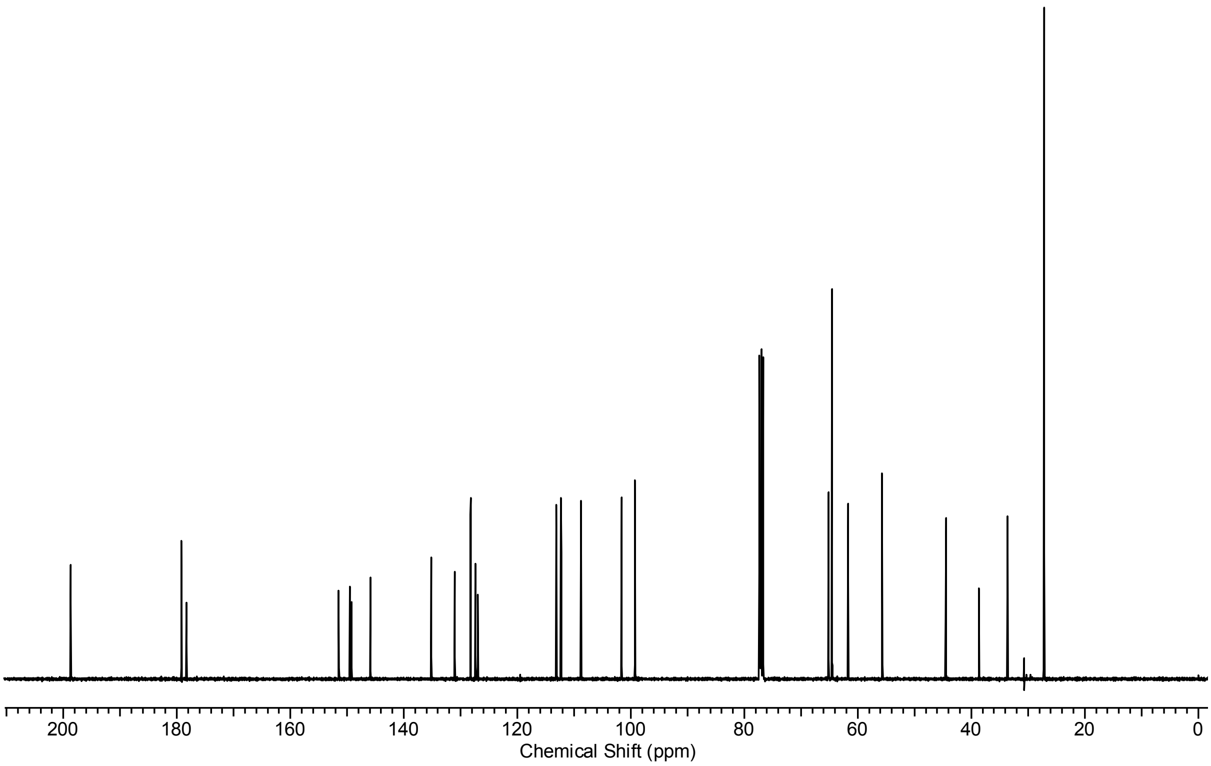

(6) 2-(6-(7,8-Dimethoxyisoquinolin-3-yl)benzo[d][1,3]dioxol-5-yl)ethyl pivalate ($CDCl_3$)

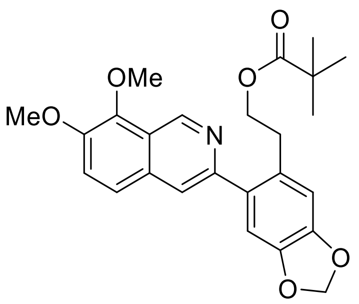

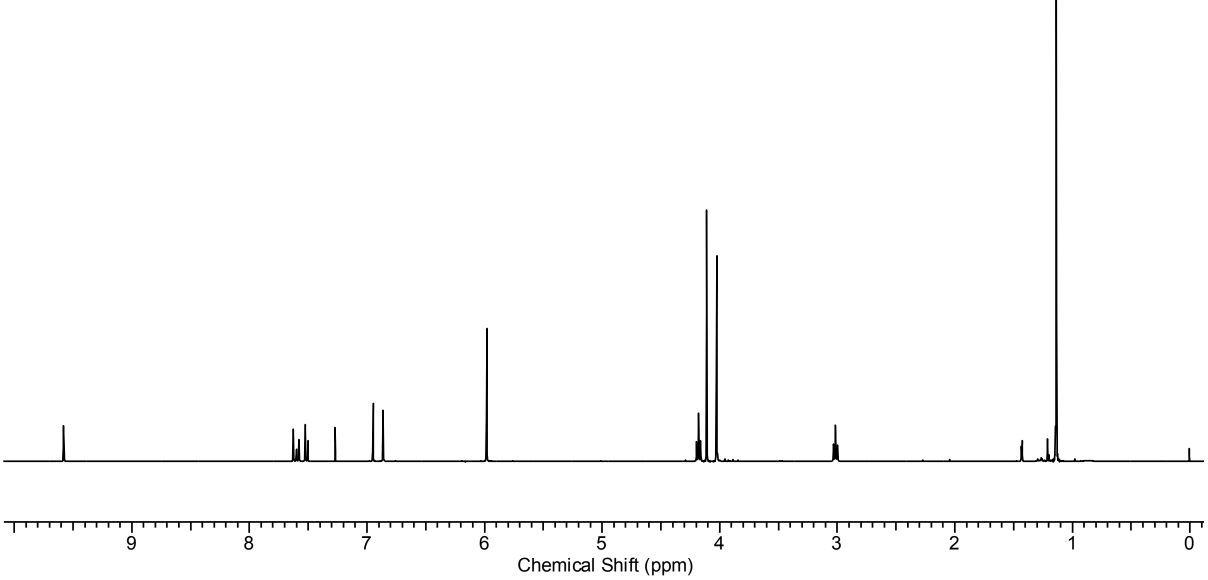

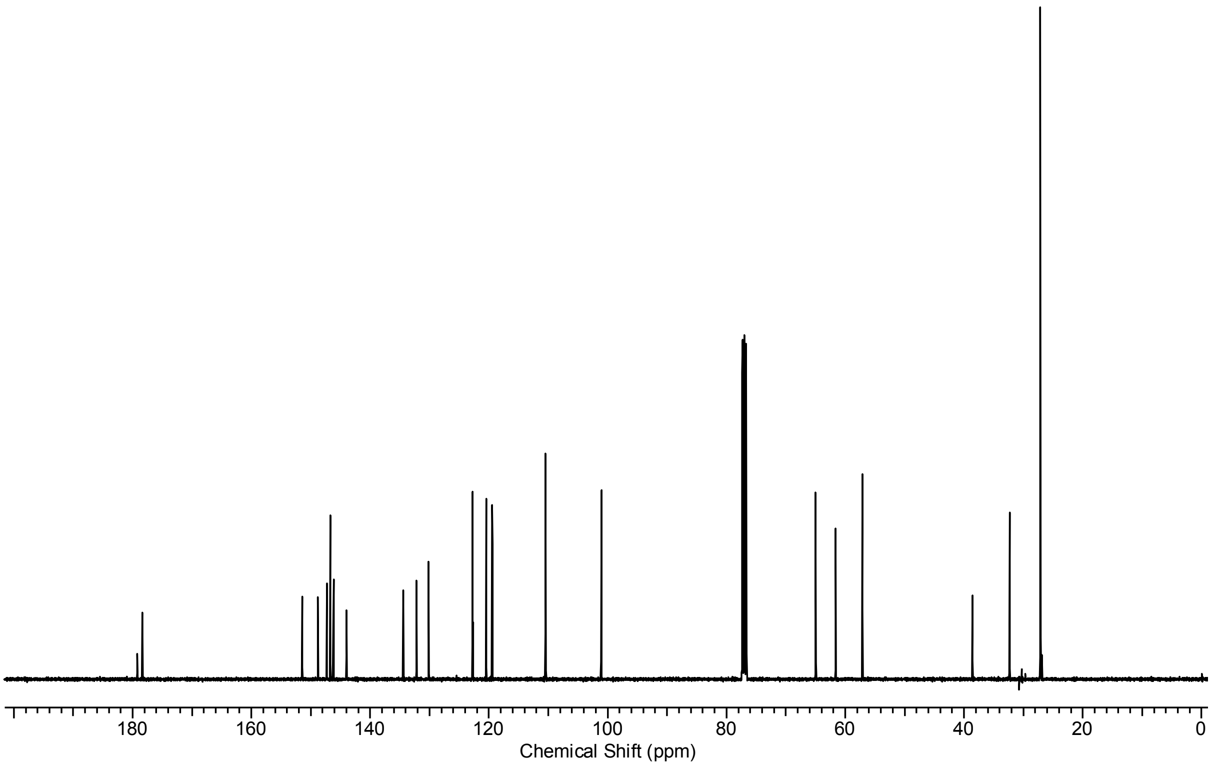

Berberine chloride (DMSO-d_6)

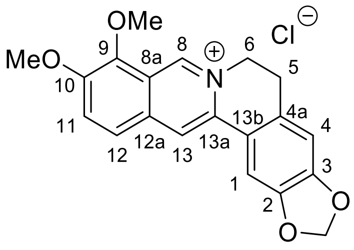

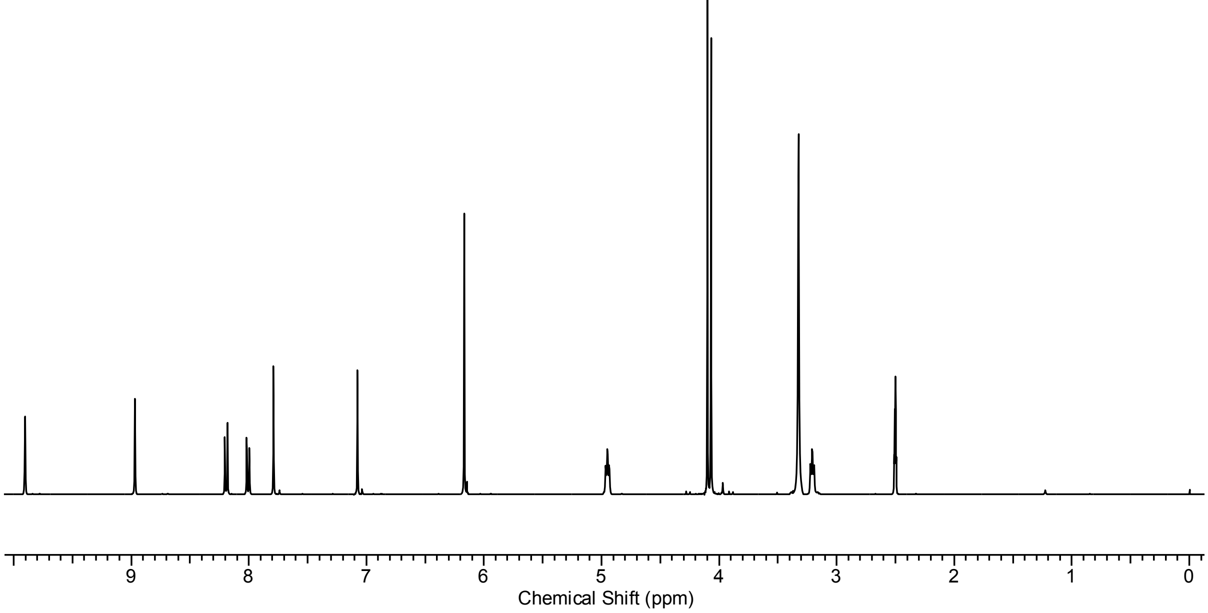

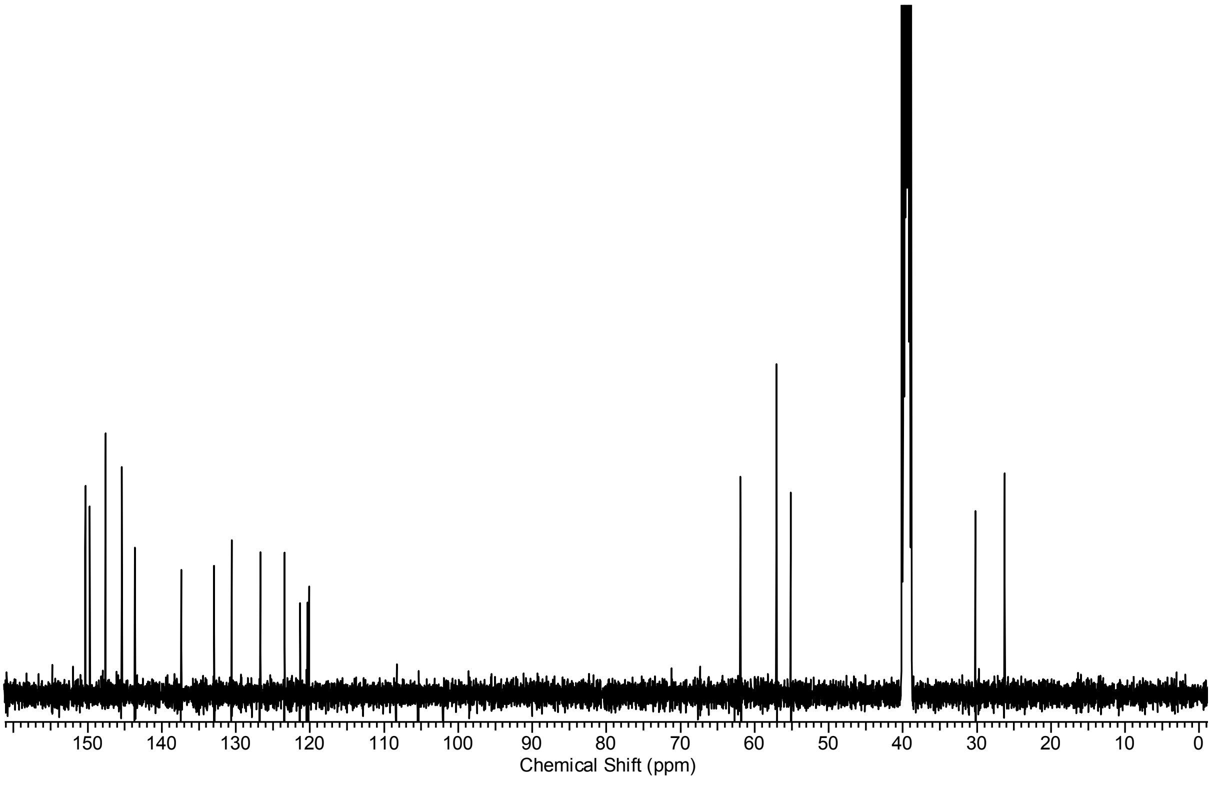

(7) 5-Bromo-6-(1,3-dioxolan-2-yl)benzo[d][1,3]dioxole ($CDCl_3$)

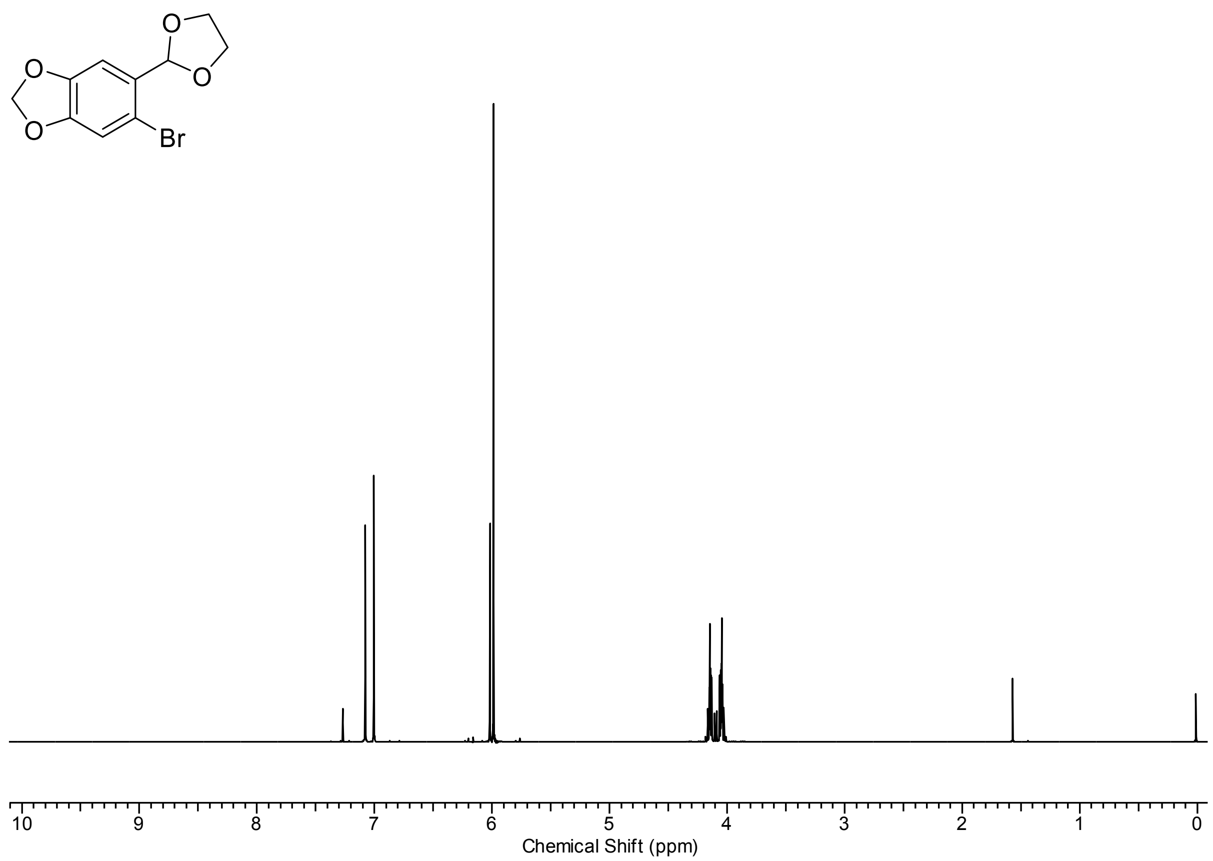

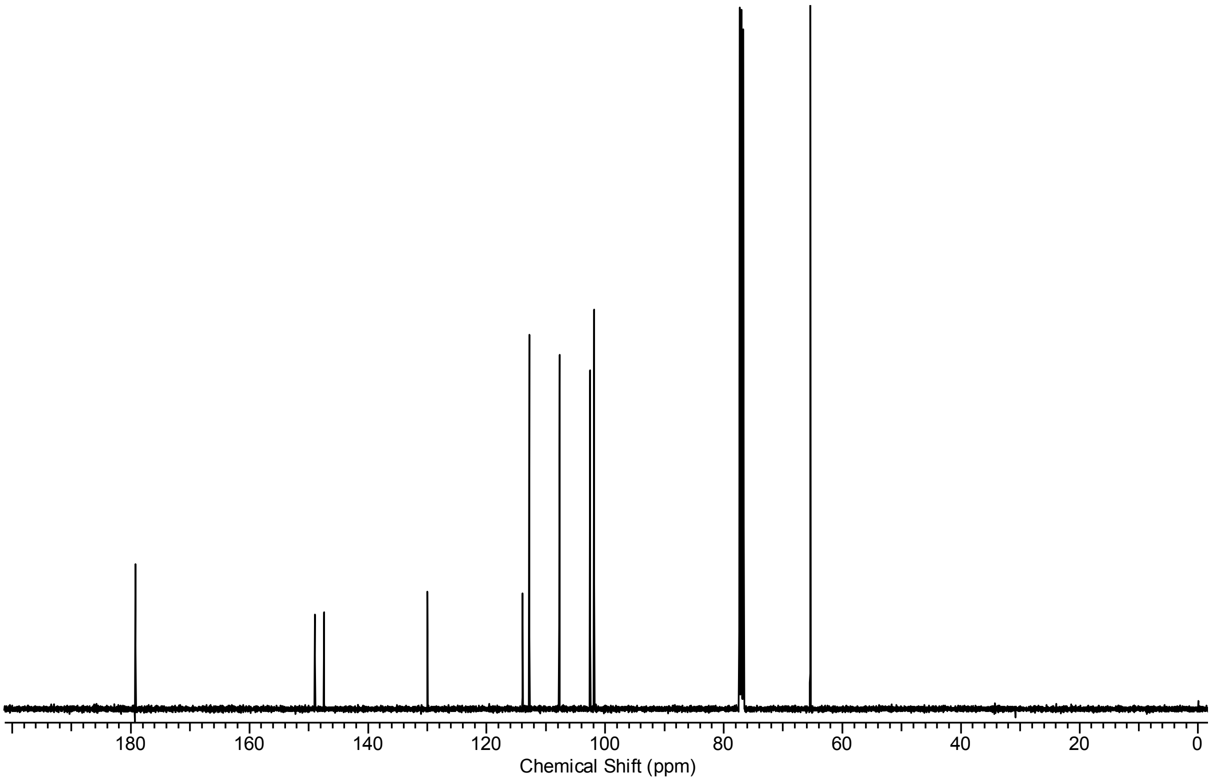

(8) 2-(6-(2-(6-(1,3-Dioxolan-2-yl)benzo[d][1,3]dioxol-5-yl)acetyl)benzo[d][1,3]dioxol-5-yl)ethyl pivalate ($CDCl_3$)

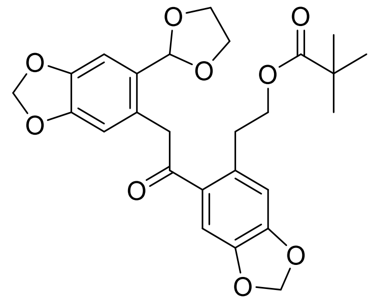

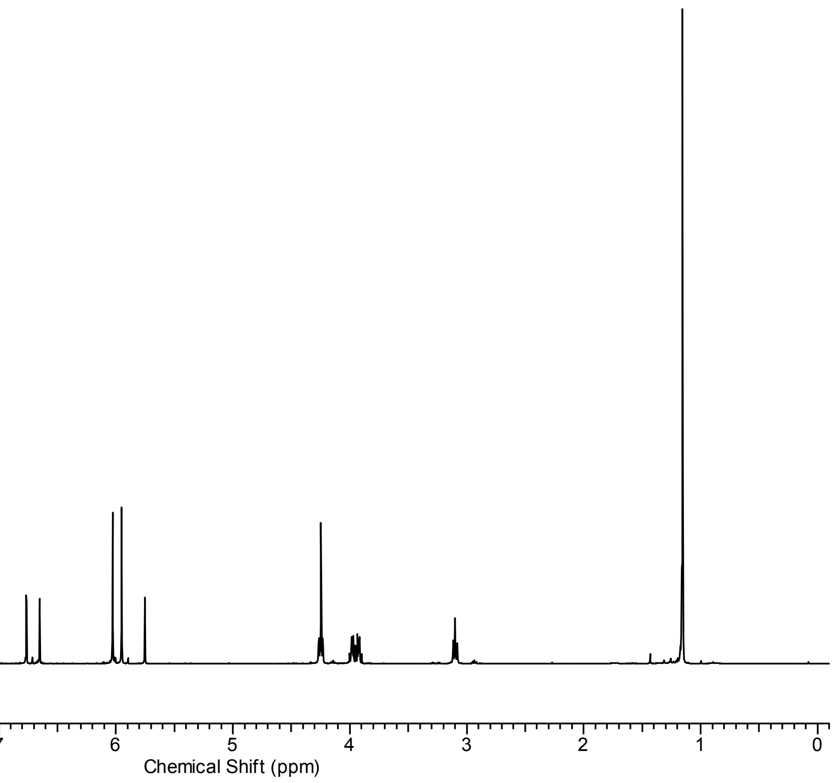

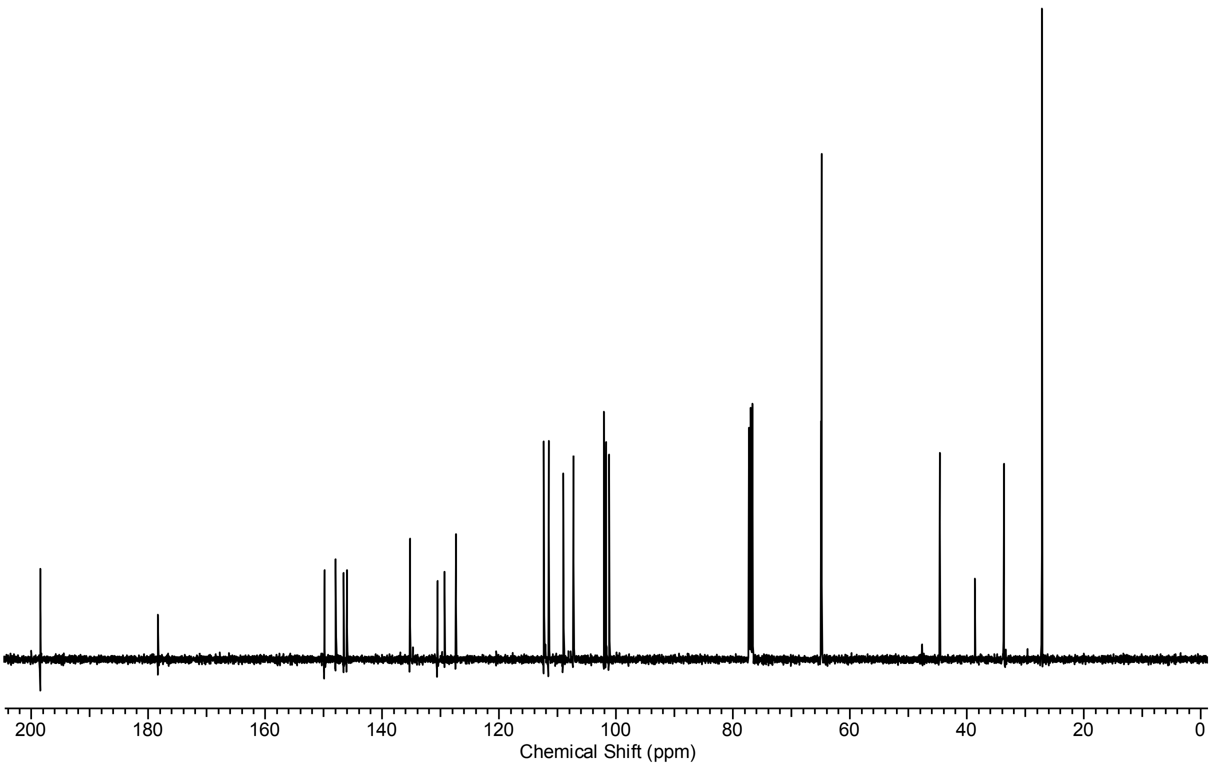

Pseudocoptisine chloride (DMSO-d_6)

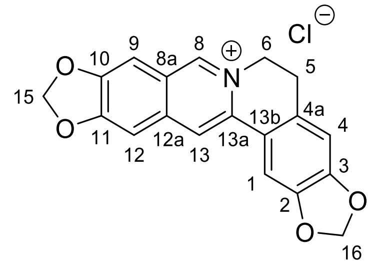

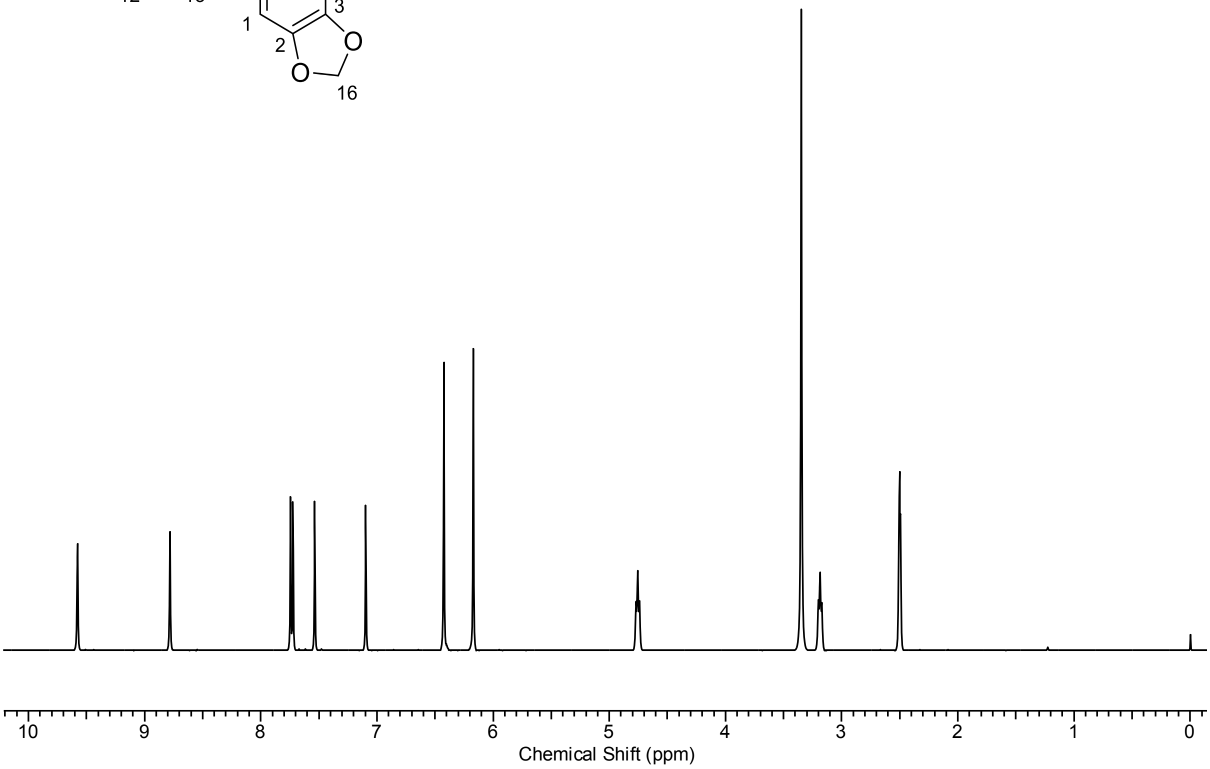

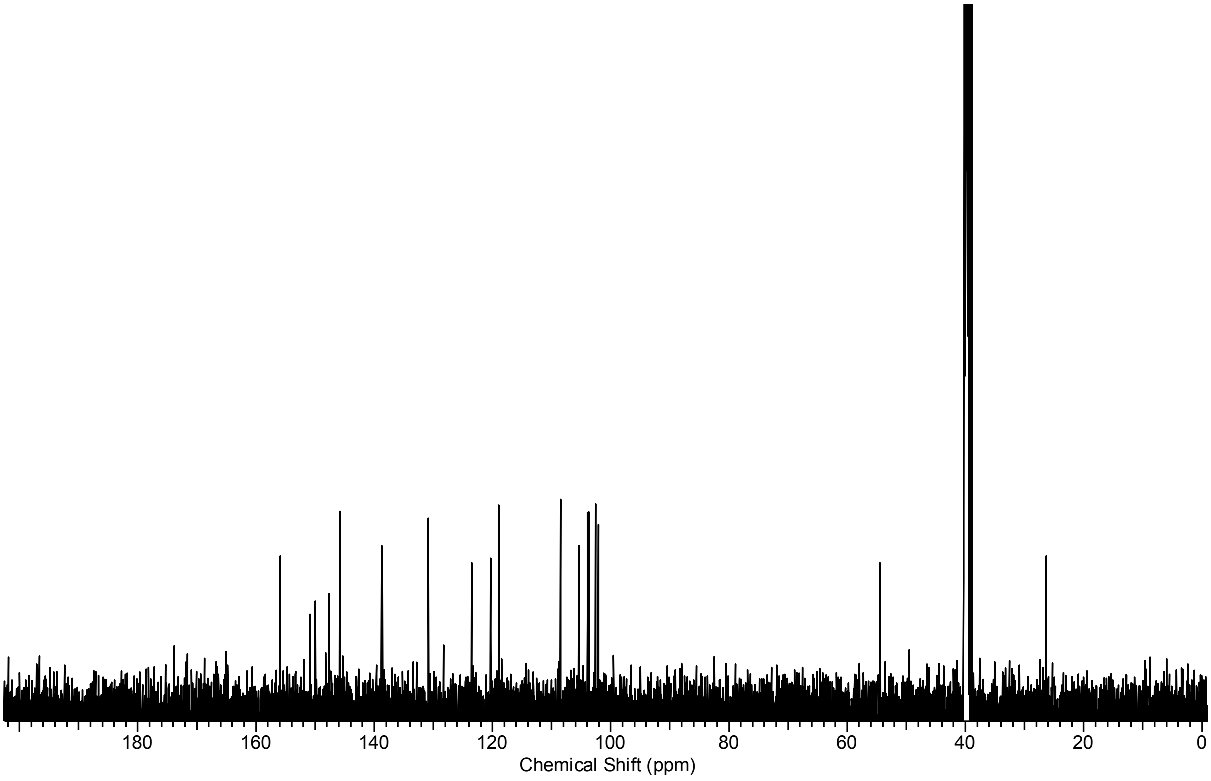

(S2) 2-(6-([1,3]Dioxolo[4,5-g]isoquinolin-7-yl)benzo[d][1,3]dioxol-5-yl)ethan-1-ol ($CDCl_3$)

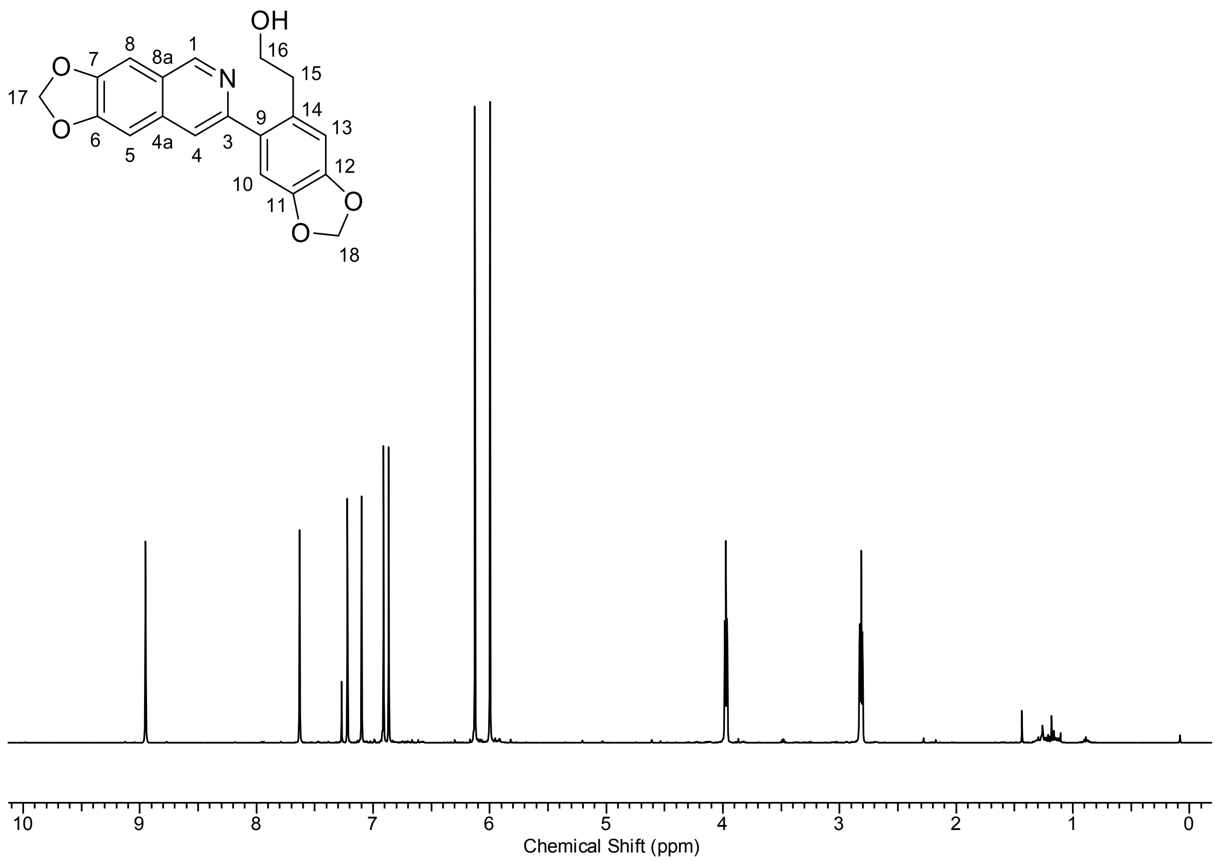

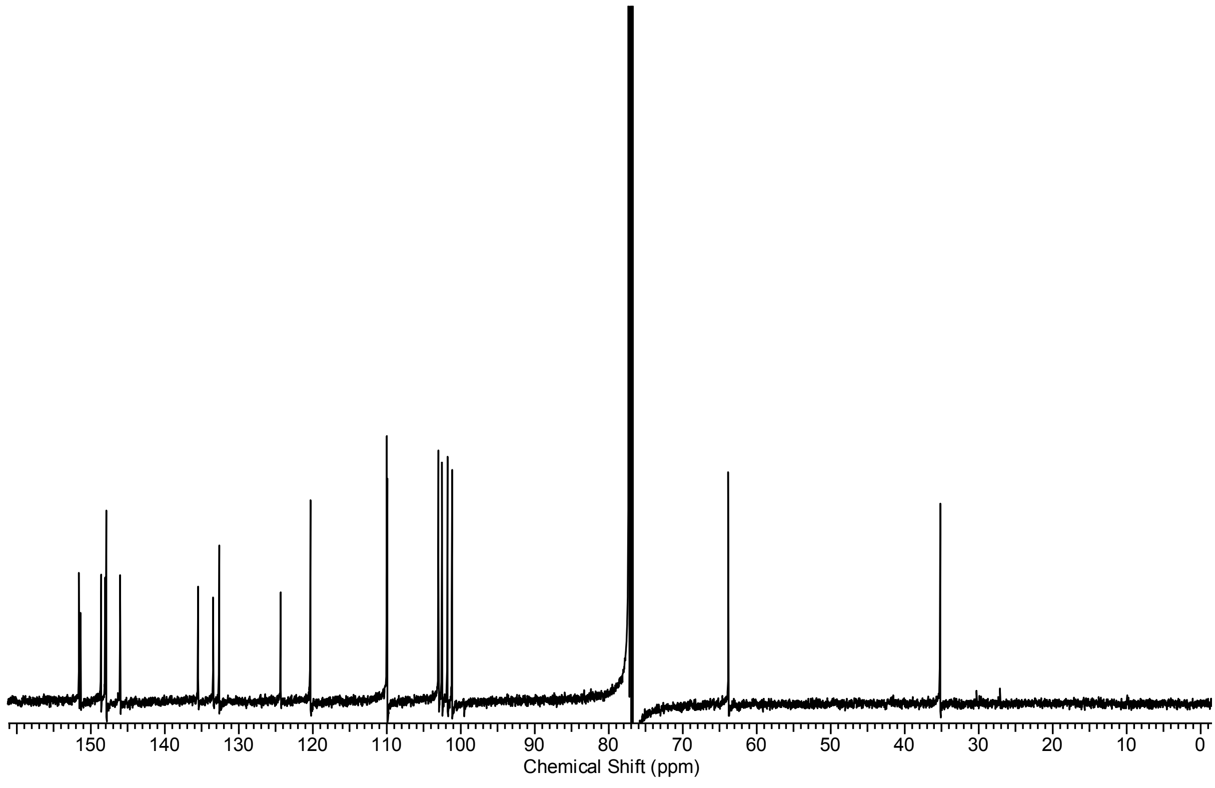

Tetrahydropseudocoptisine ($CDCl_3$)

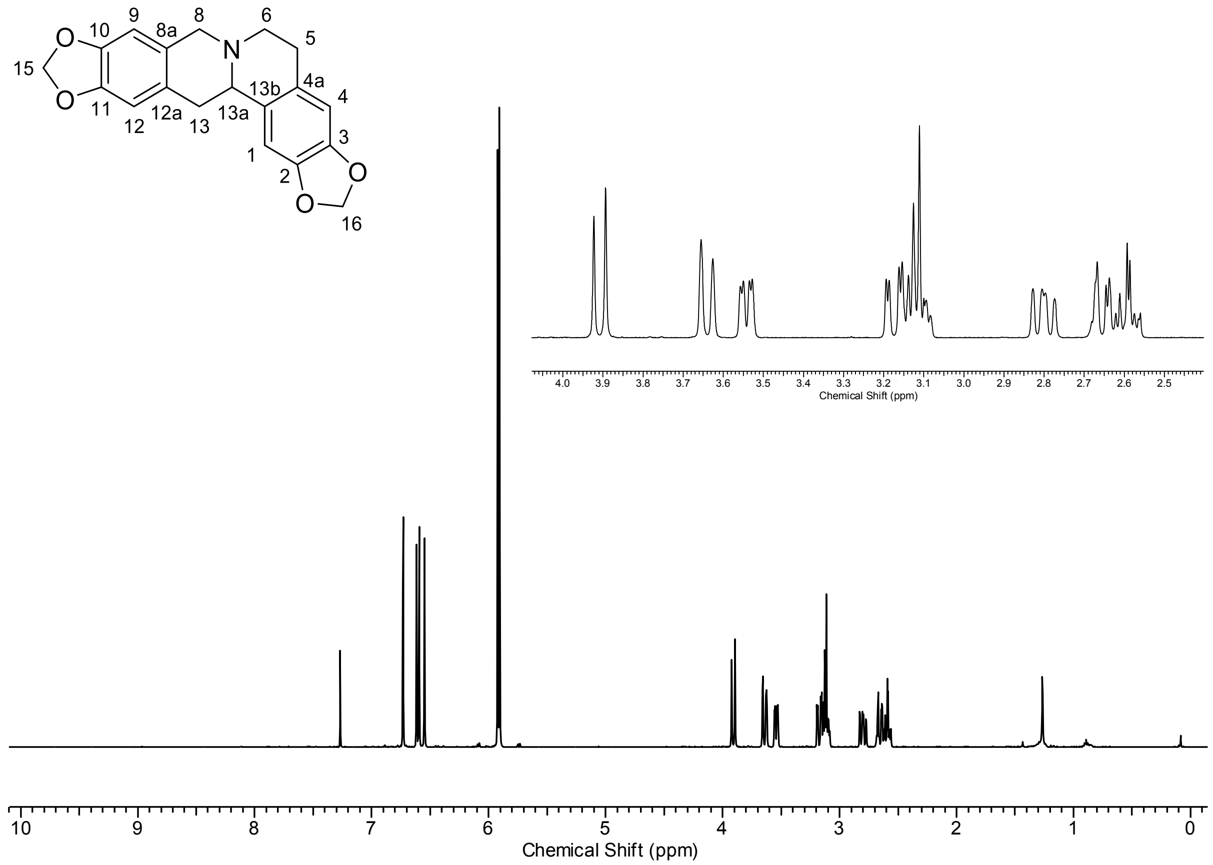

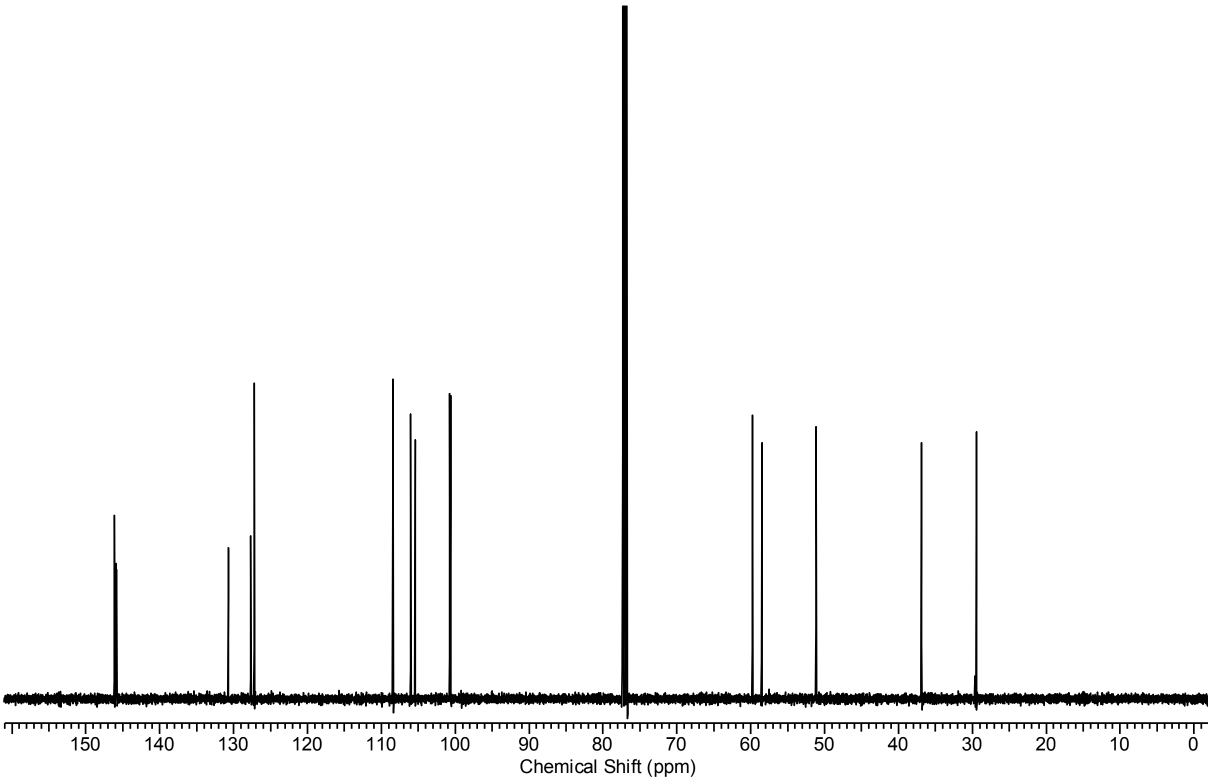

(S3) 3,4-Dimethoxyphenethyl pivalate ($CDCl_3$)

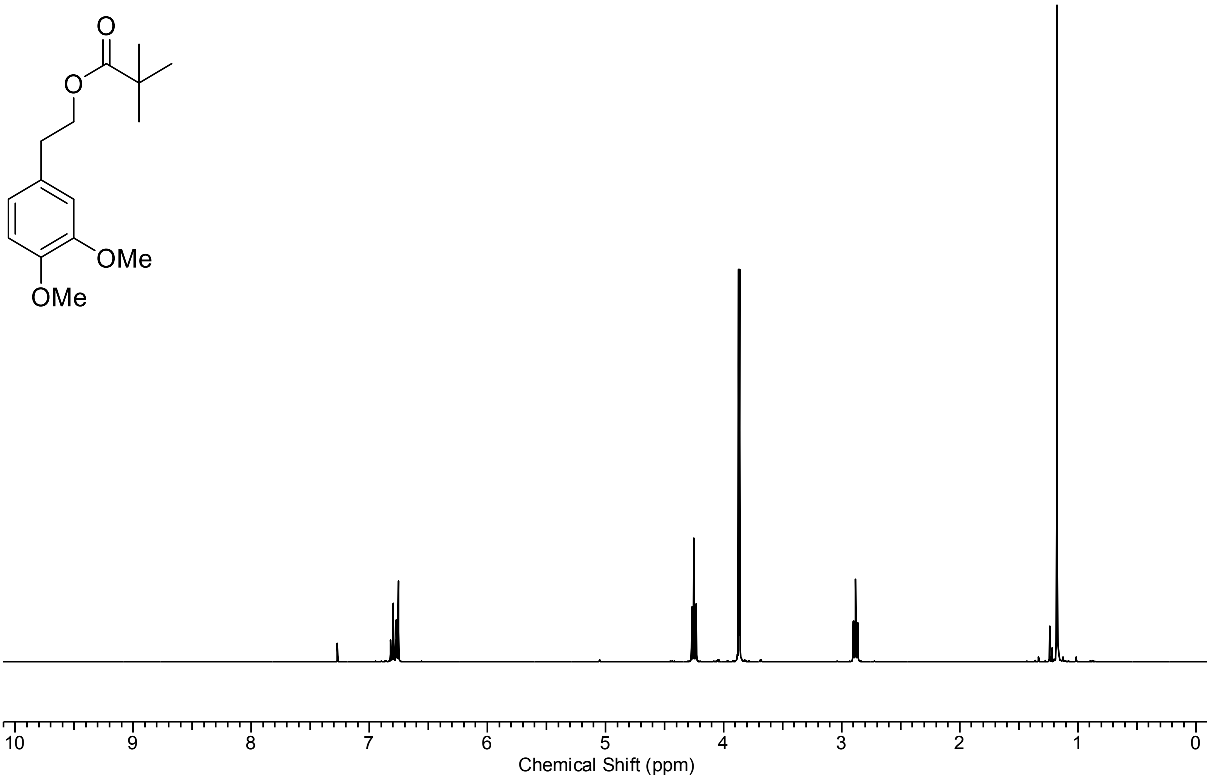

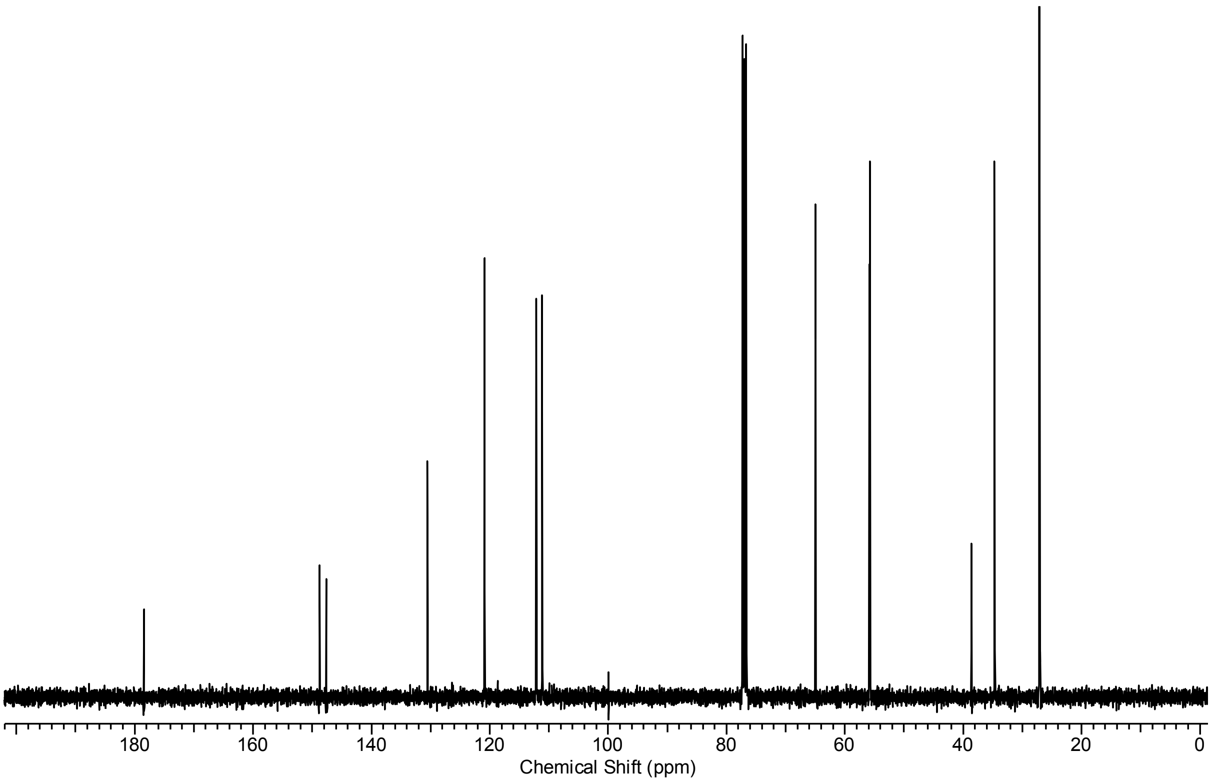

(10) 2-Acetyl-4,5-dimethoxyphenethyl pivalate ($CDCl_3$)

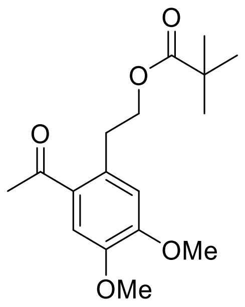

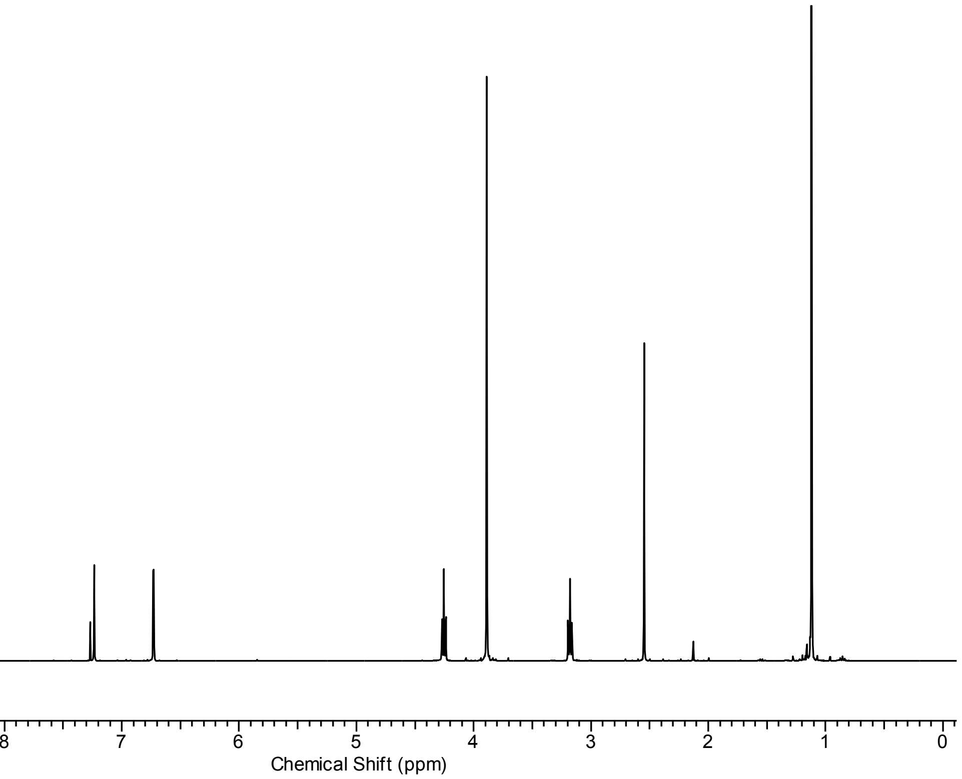

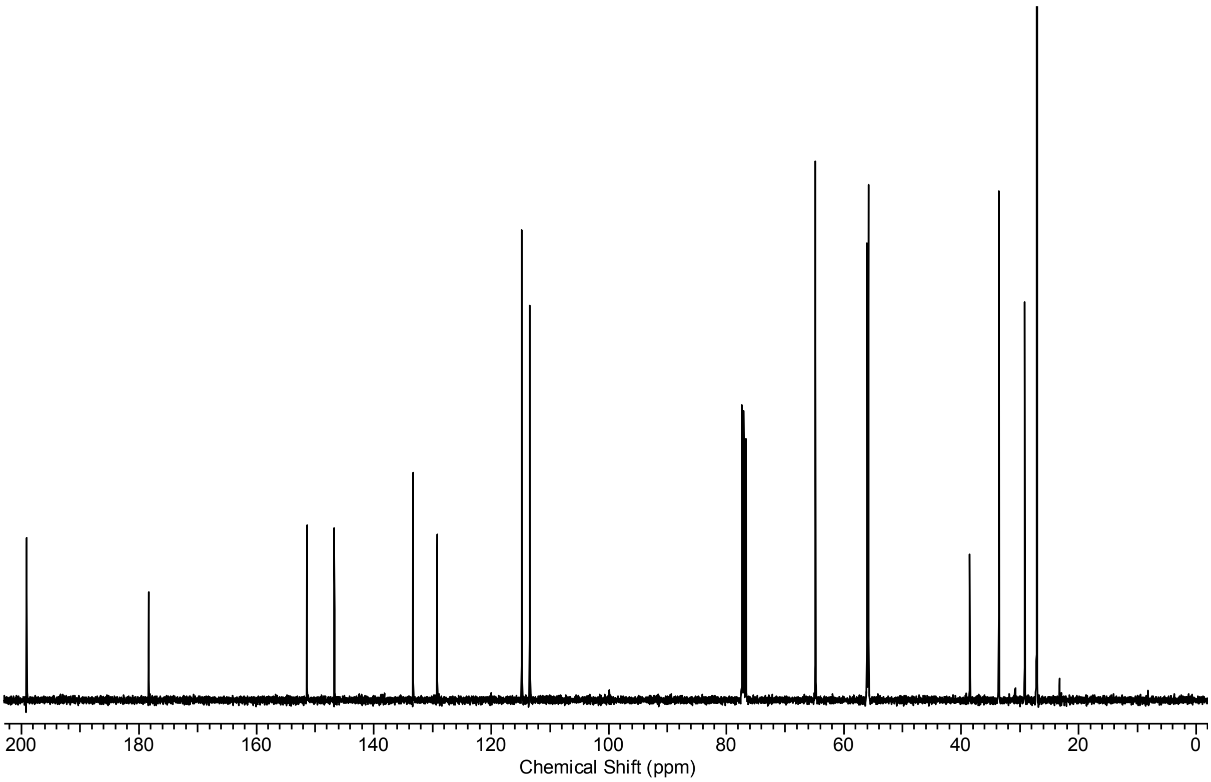

(11) 2-(2-(2-(1,3-Dioxolan-2-yl)-3,4-dimethoxyphenyl)acetyl)-4,5-dimethoxyphenethyl pivalate ($CDCl_3$)

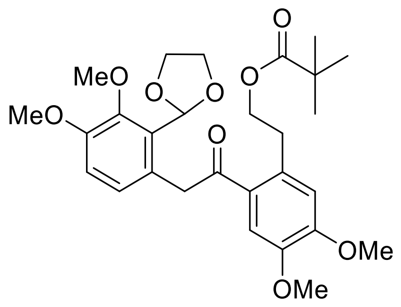

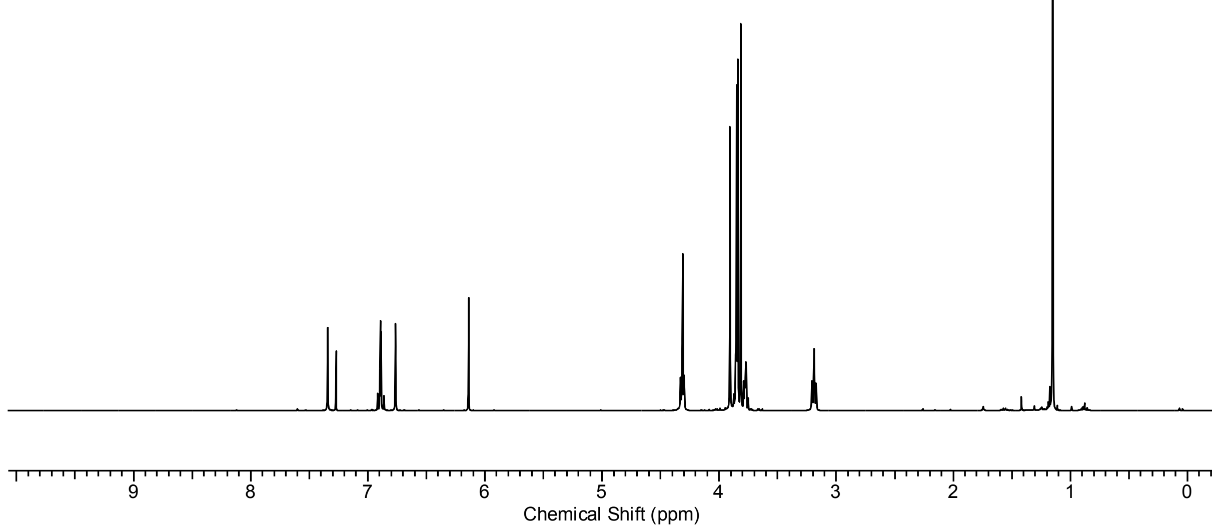

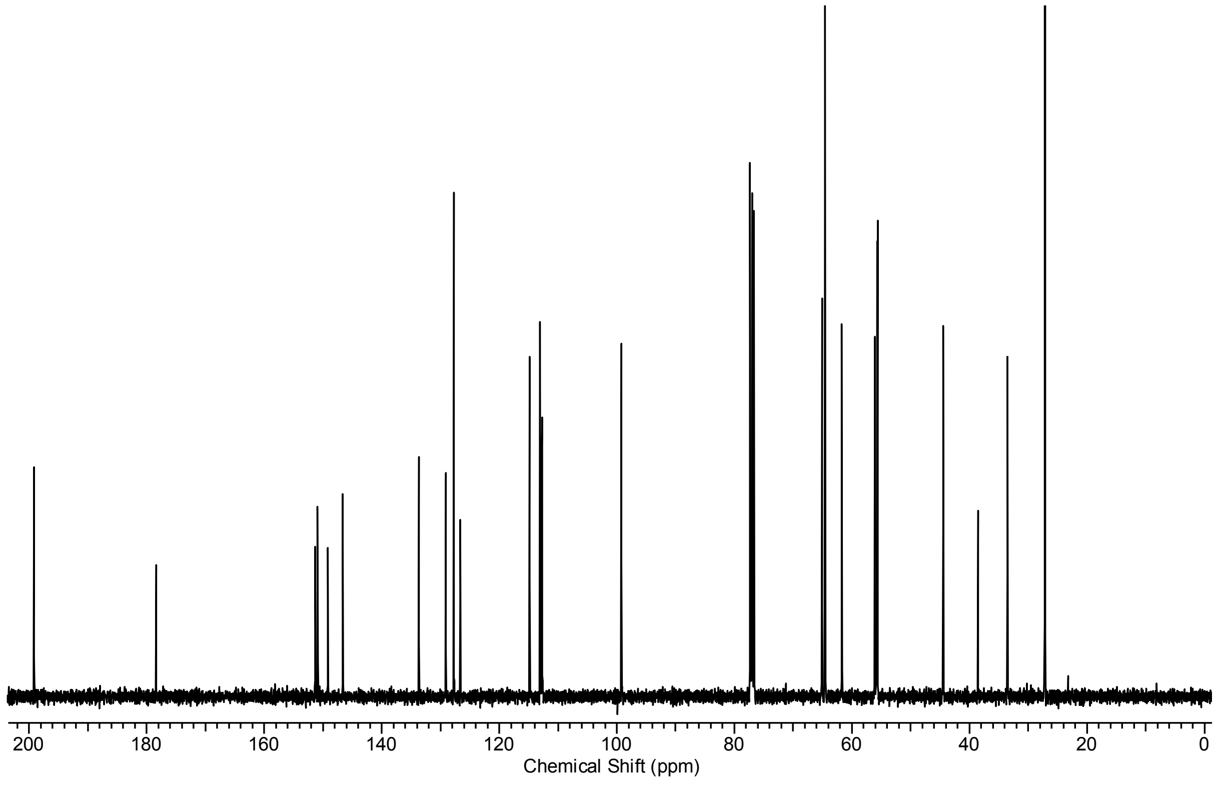

Palmatine chloride (DMSO-d_6)

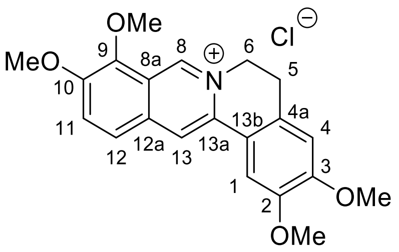

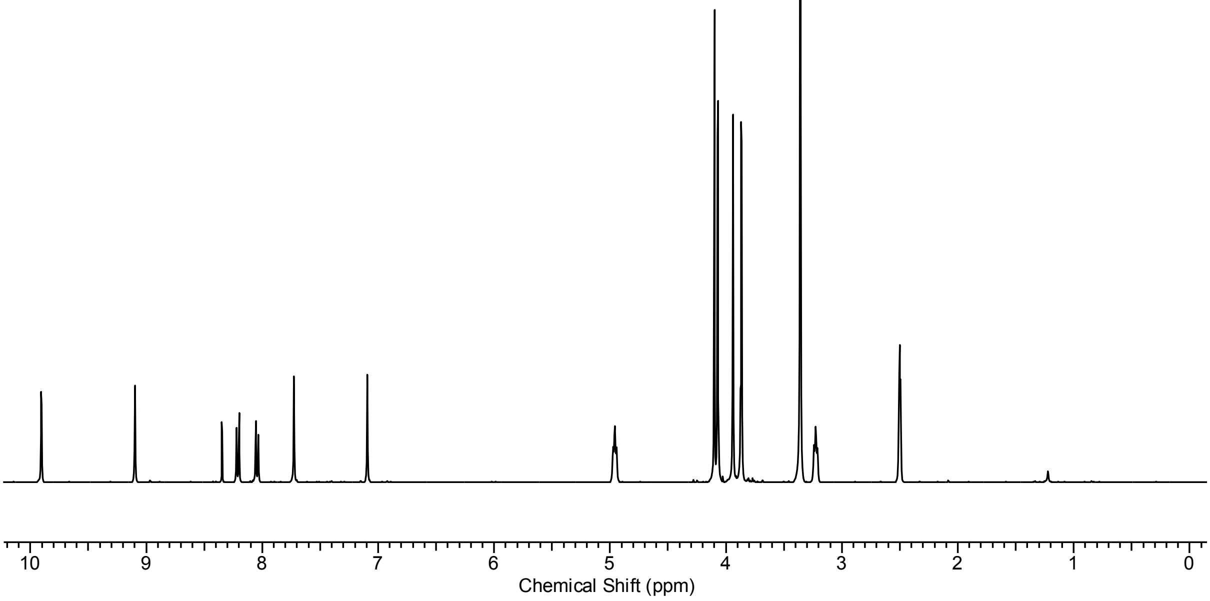

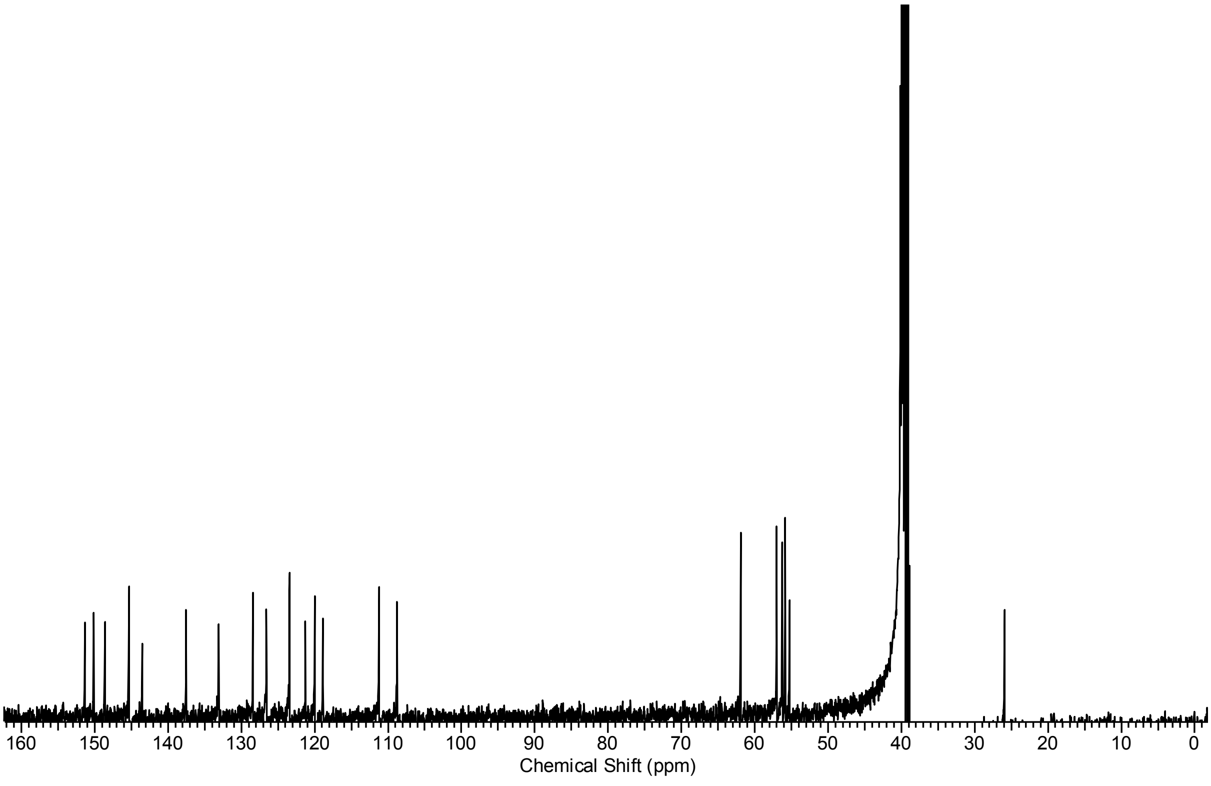

(12) 2-(2-(2-(1,3-Dioxolan-2-yl)-3,4-dimethoxyphenyl)propanoyl)-4,5-dimethoxyphenethyl pivalate ($CDCl_3$)

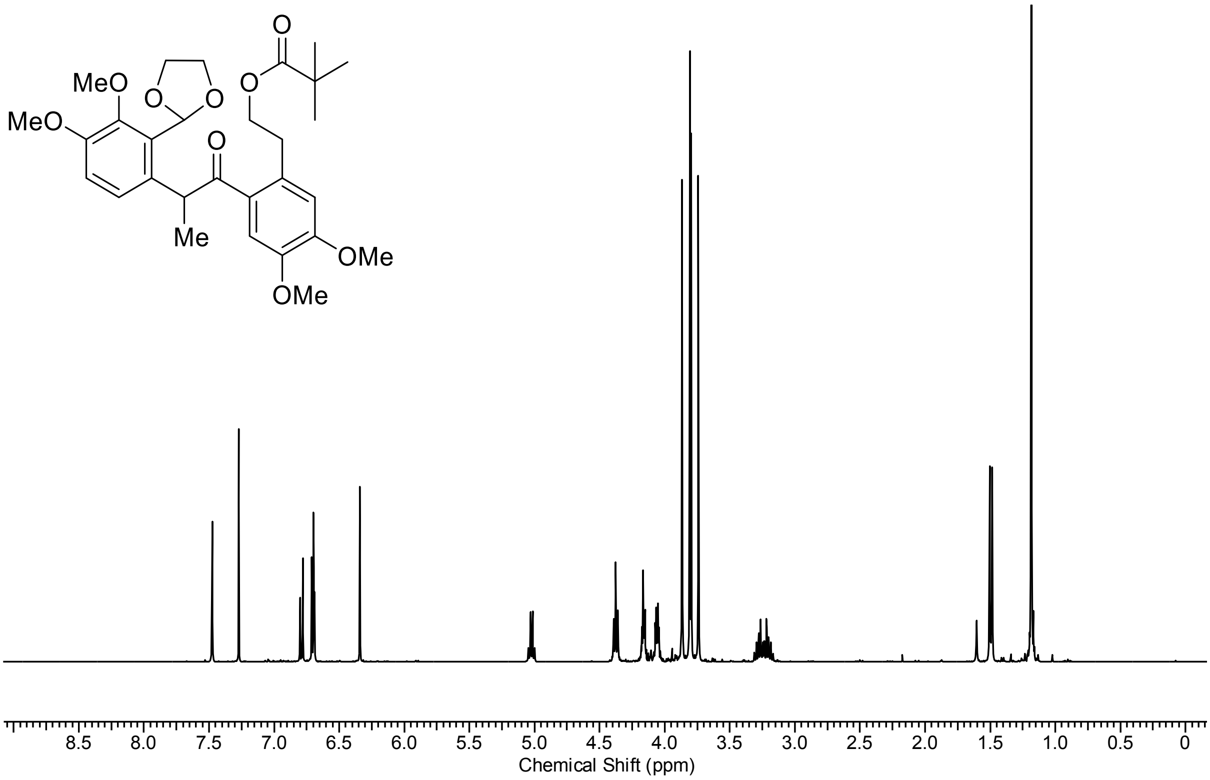

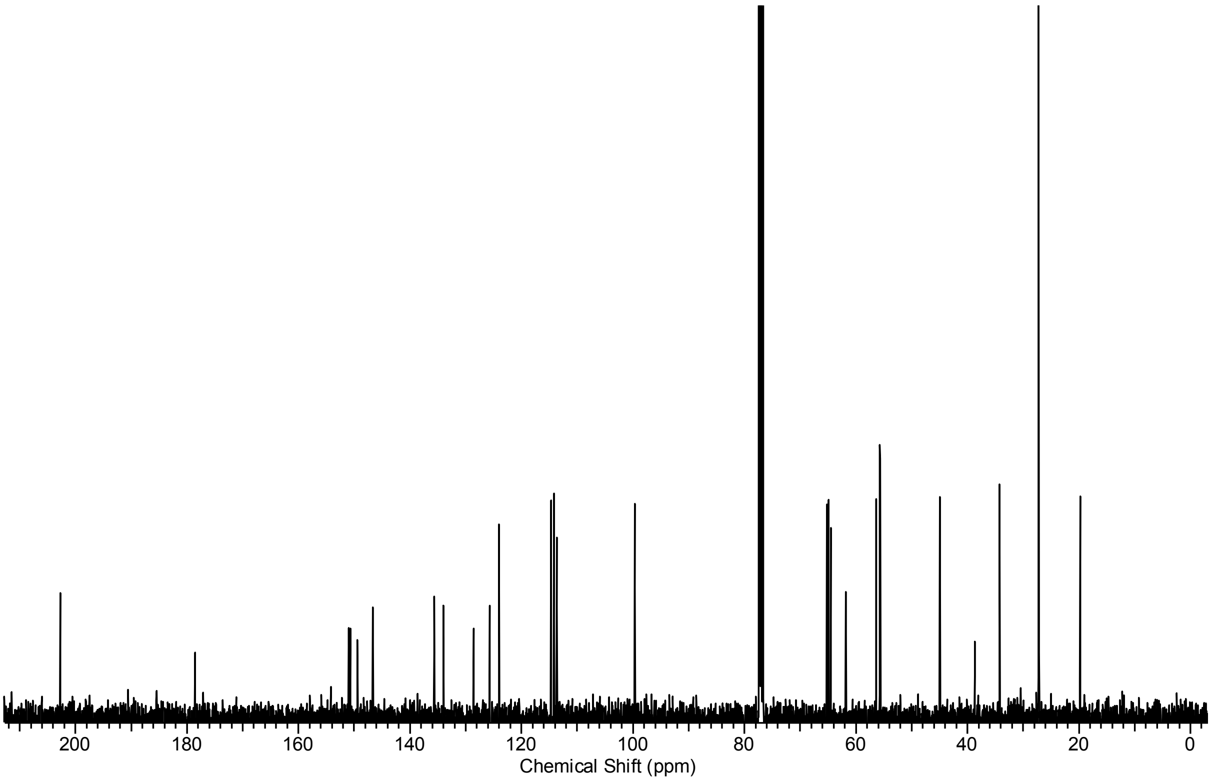

(13) 2-(7,8-Dimethoxy-4-methylisoquinolin-3-yl)-4,5-dimethoxyphenethyl pivalate ($CDCl_3$)

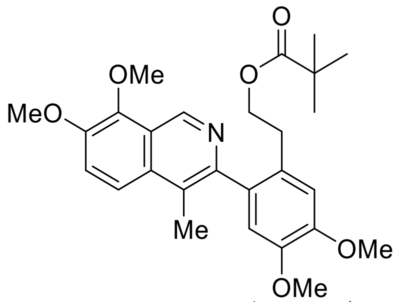

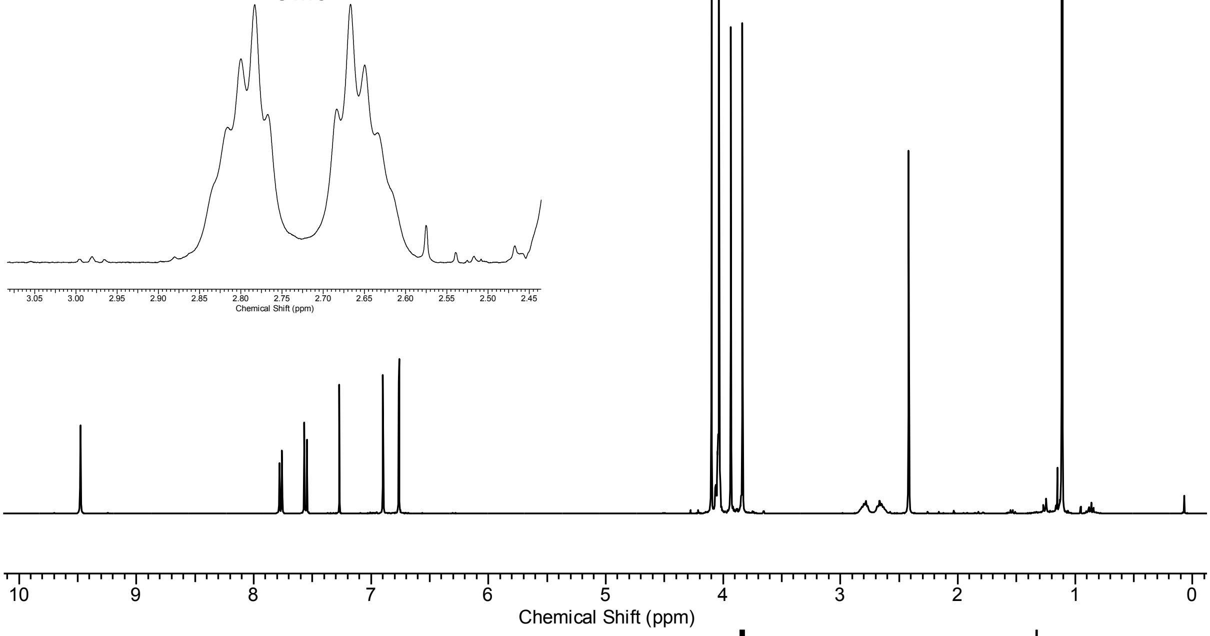

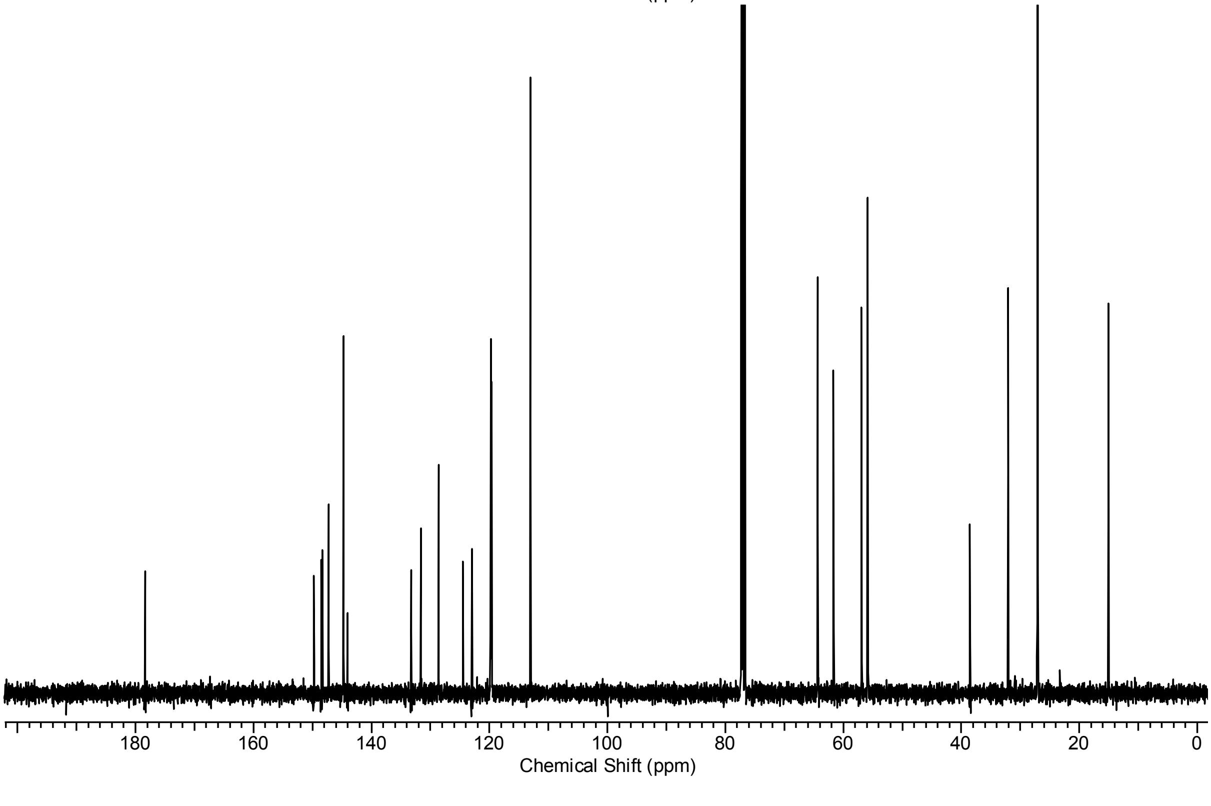

(S4) 2-(1-Ethoxy-7,8-dimethoxy-4-methyl-1H-isochromen-3-yl)-4,5-dimethoxyphenethyl pivalate (S4) ($CDCl_3$)

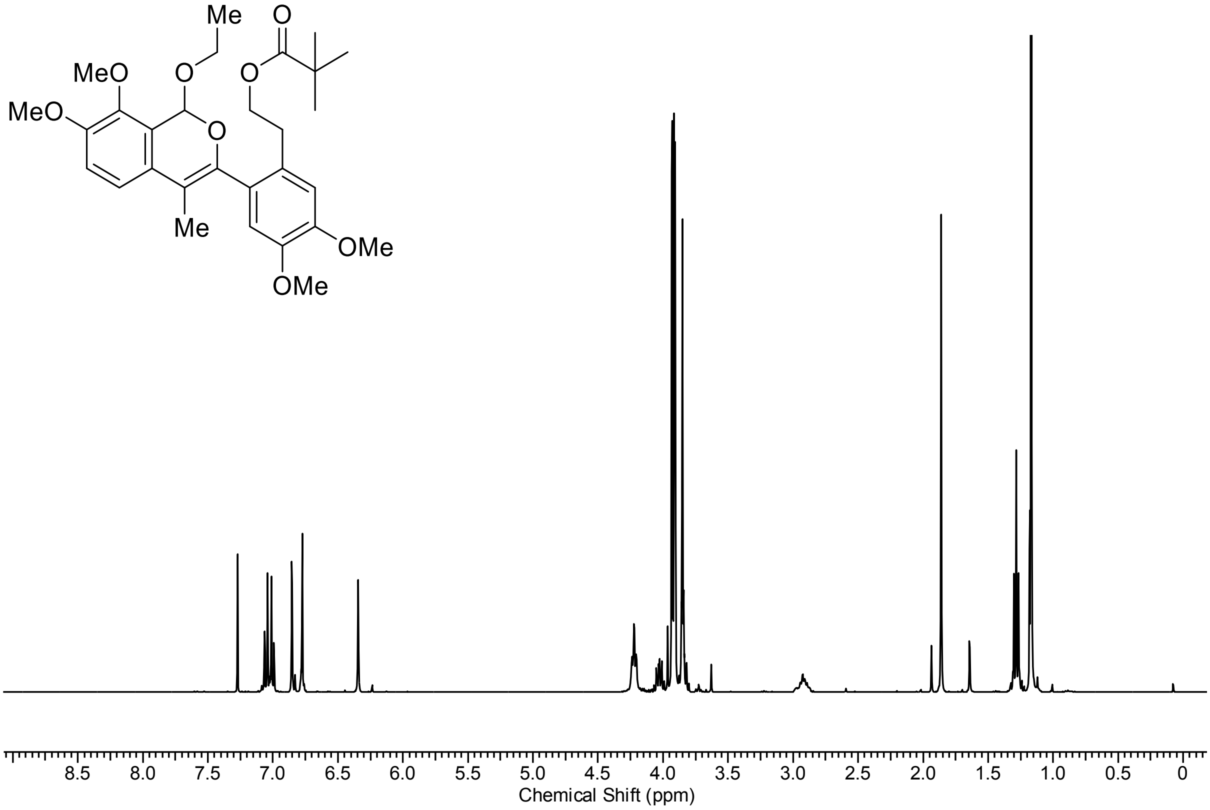

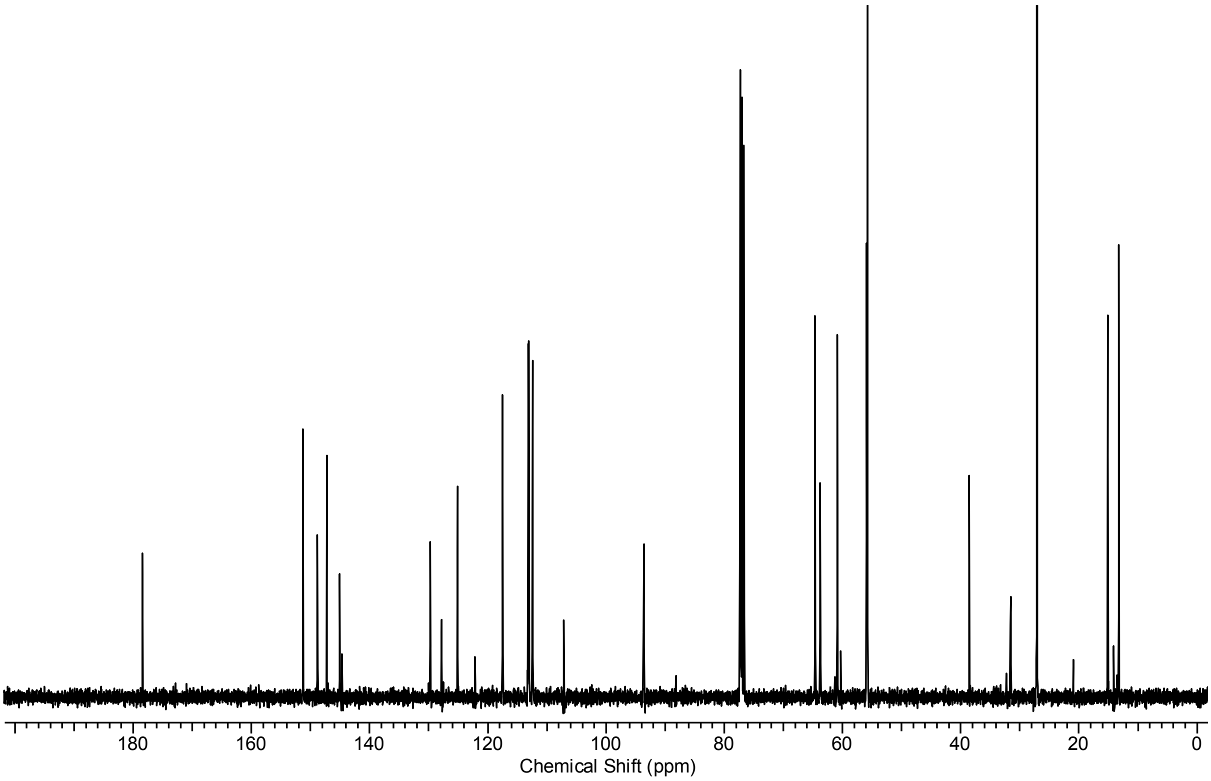

(S5) 2-(2-(7,8-Dimethoxy-4-methylisoquinolin-3-yl)-4,5-dimethoxyphenyl)ethan-1-ol ($CDCl_3$)

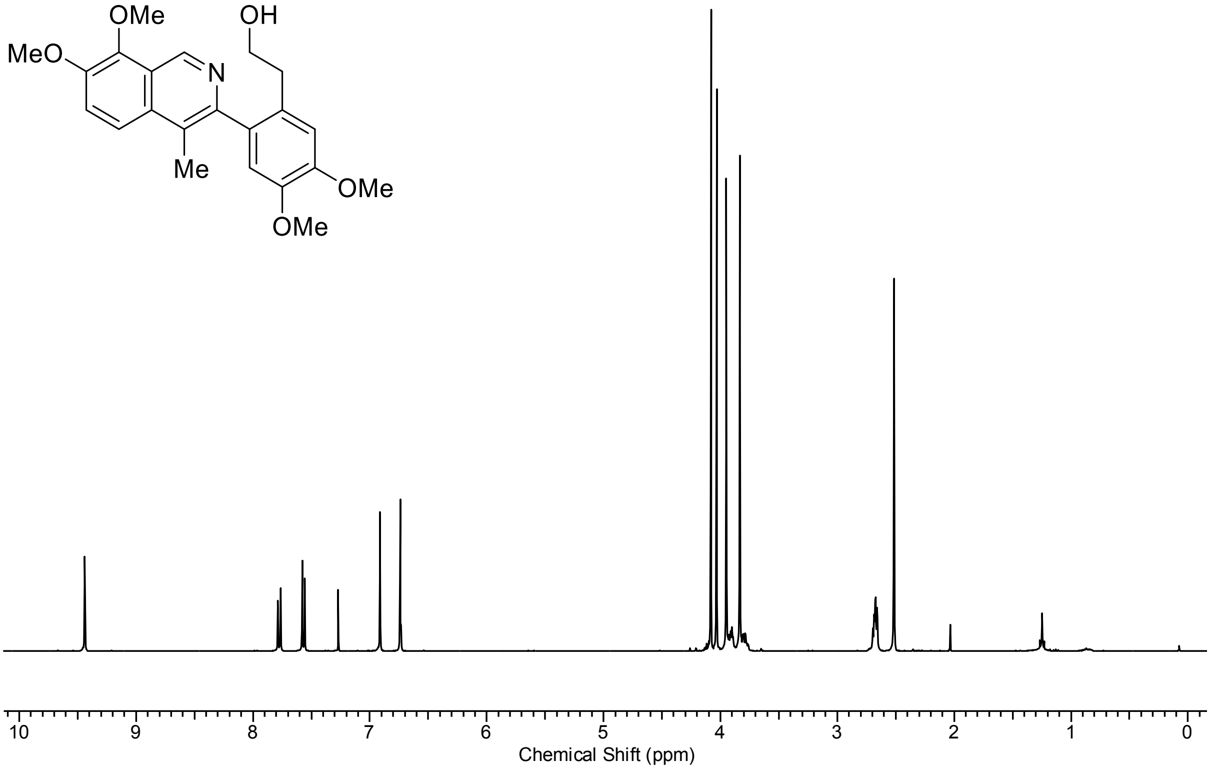

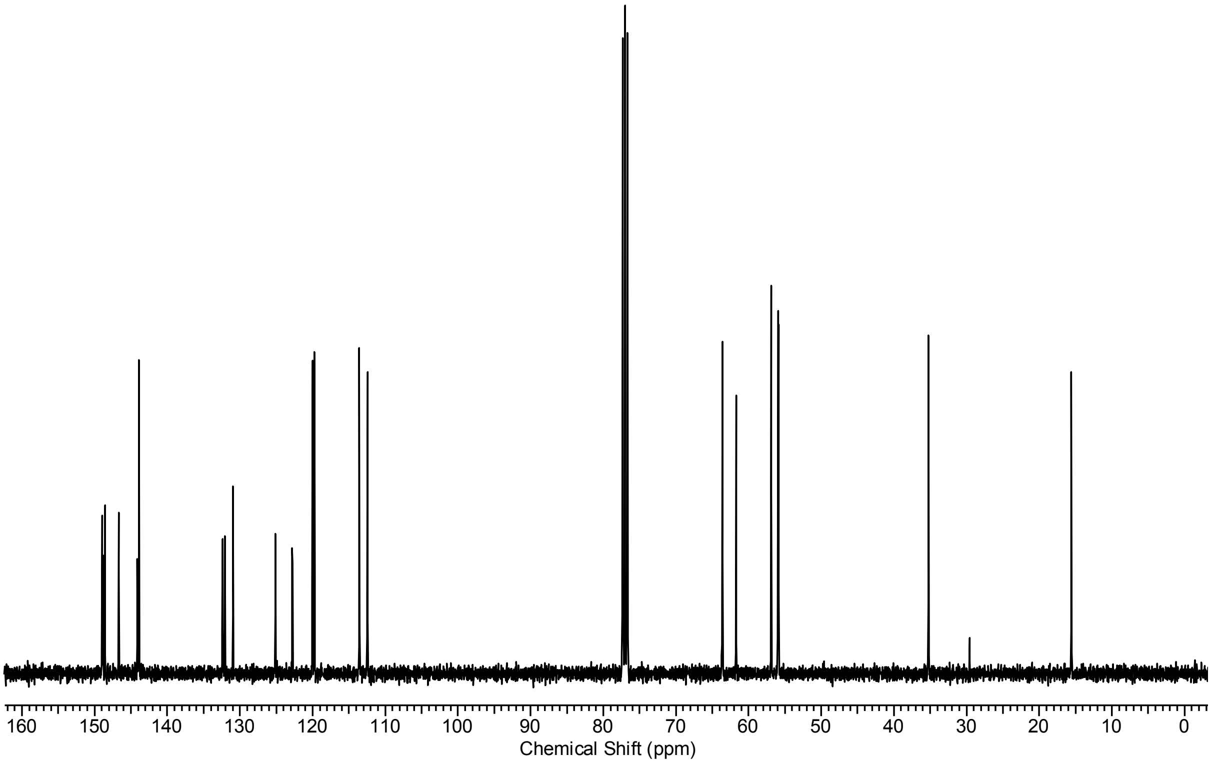

Dehydrocorydaline chloride (DMSO-d_6)

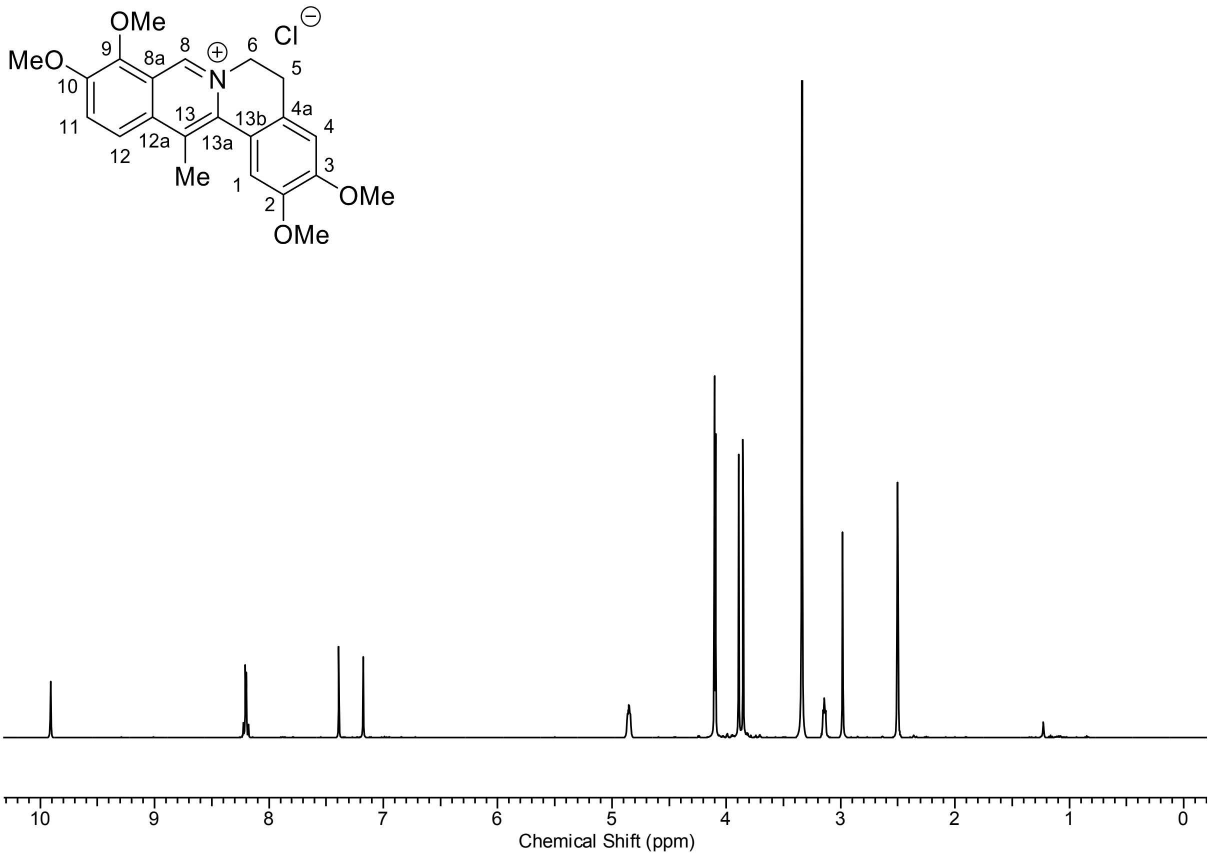

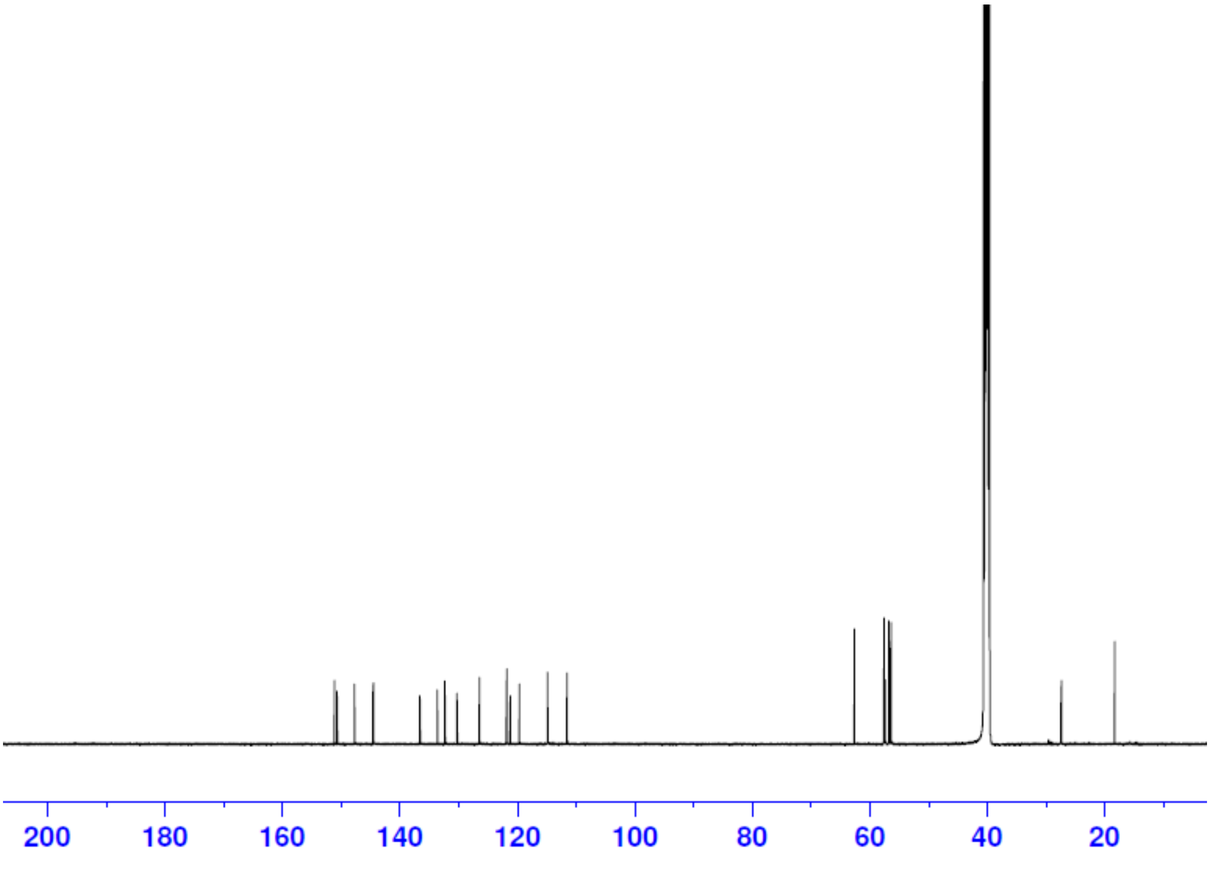

(14) 2-(2-Bromo-5-fluorophenyl)-1,3-dioxolane ($CDCl_3$)

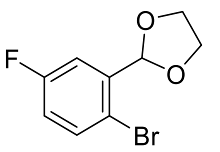

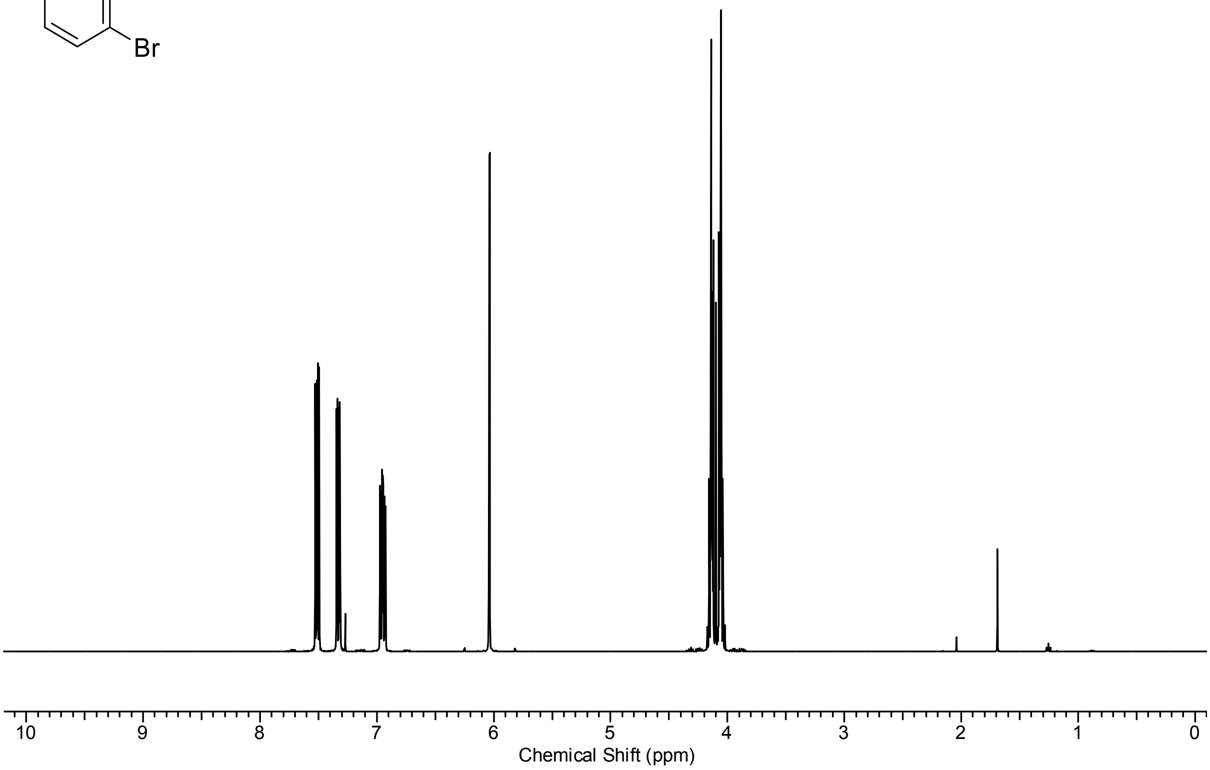

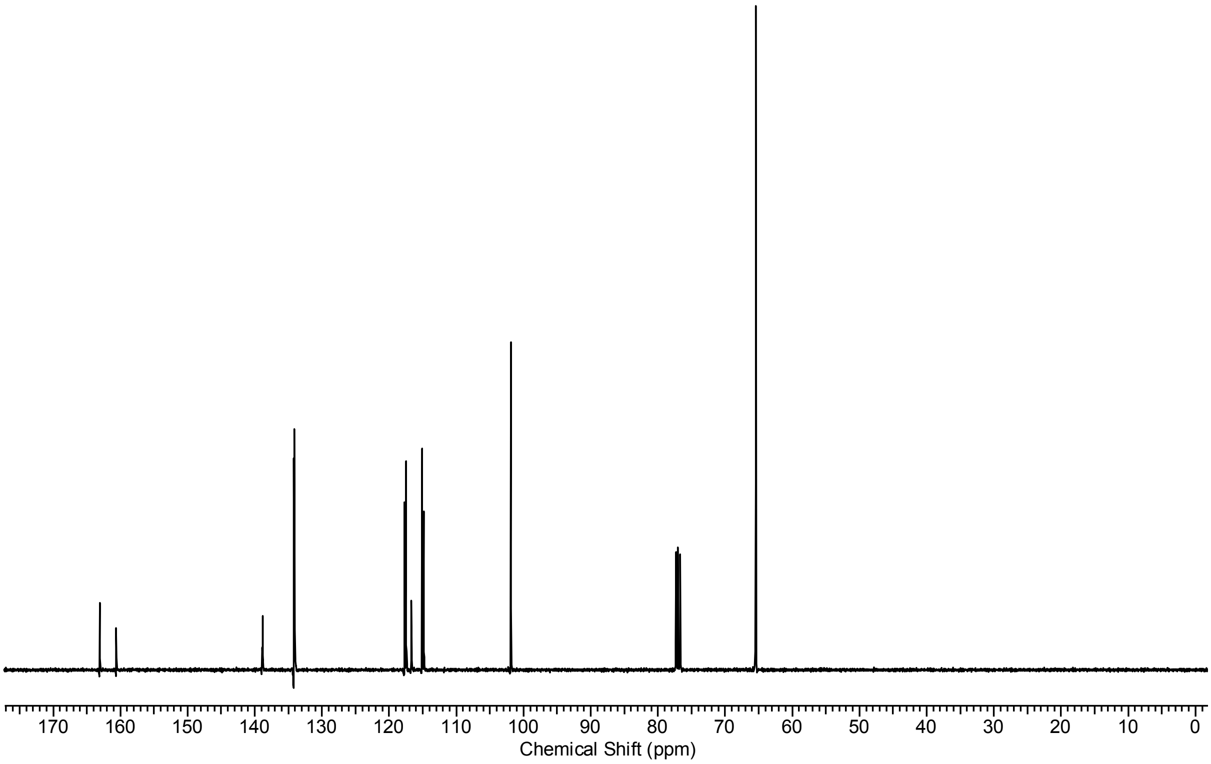

(15) 2-(6-(2-(2-(1,3-Dioxolan-2-yl)-4-fluorophenyl)acetyl)benzo[d][1,3]dioxol-5-yl)ethyl pivalate ($CDCl_3$)

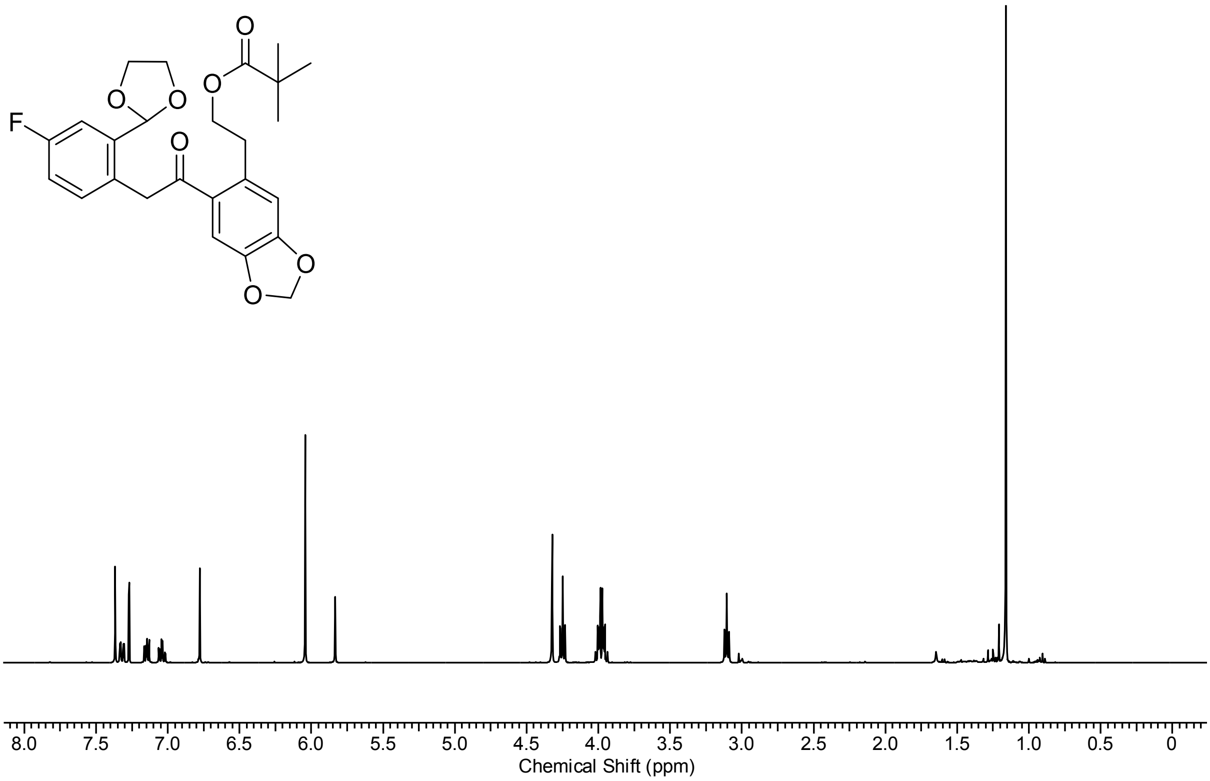

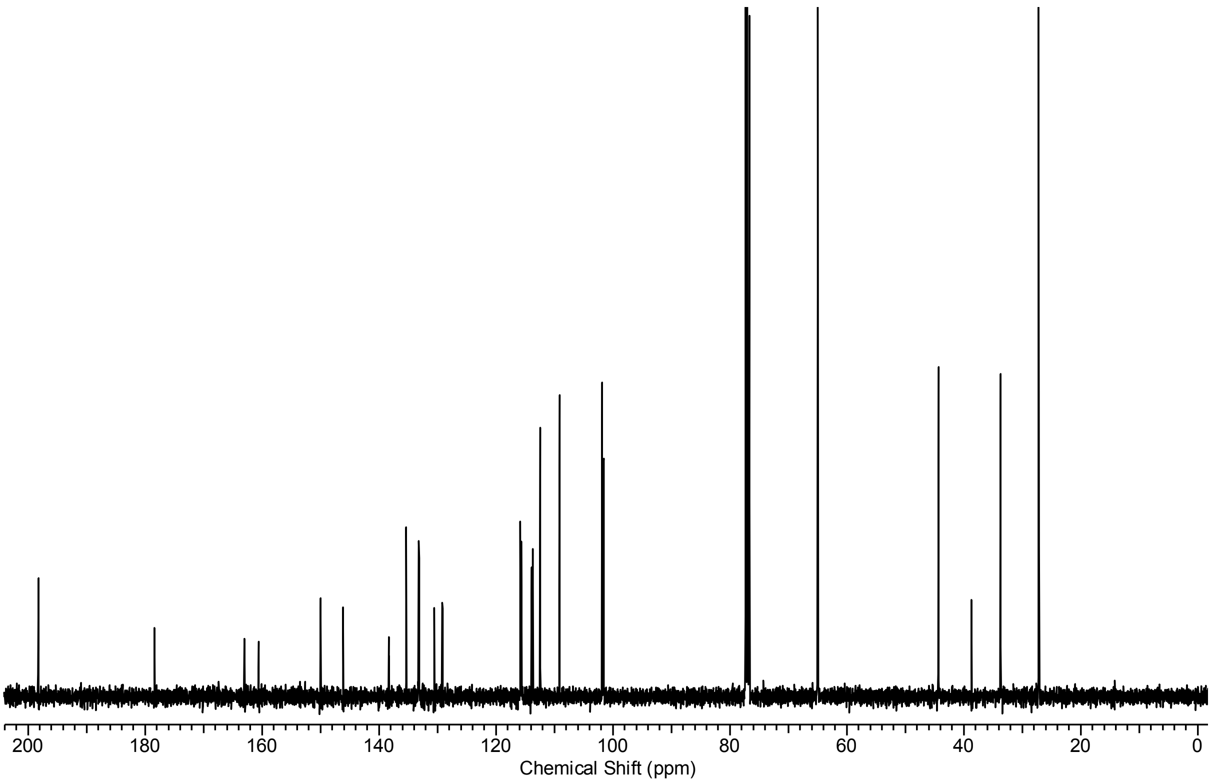

(S6) 2-(6-(7-Fluoroisoquinolin-3-yl)benzo[d][1,3]dioxol-5-yl)ethyl pivalate ($CDCl_3$)

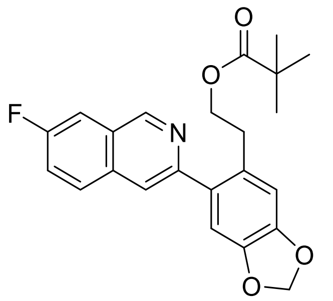

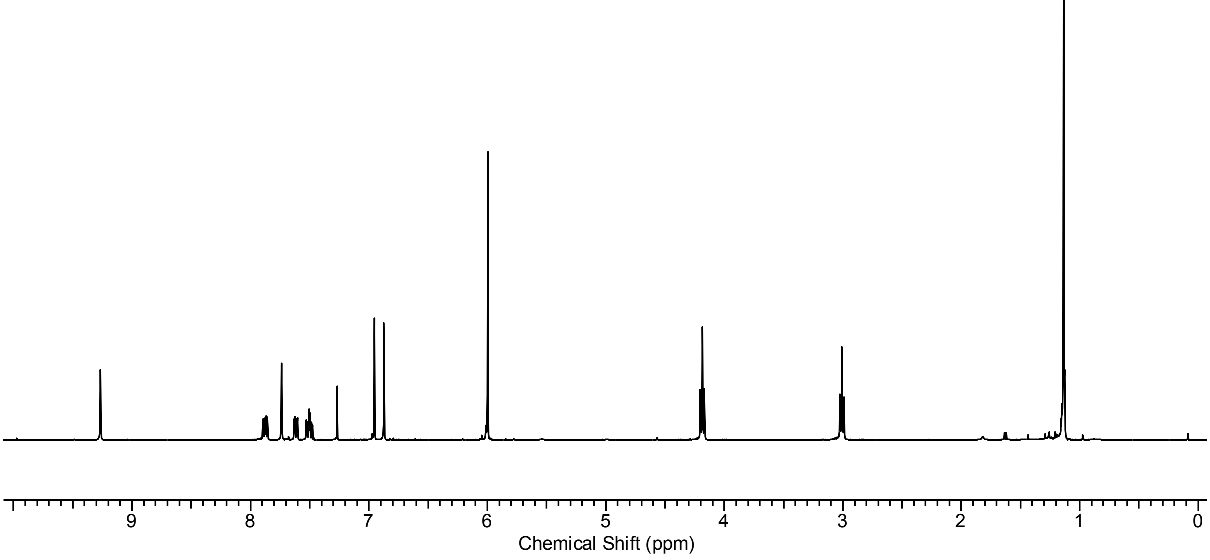

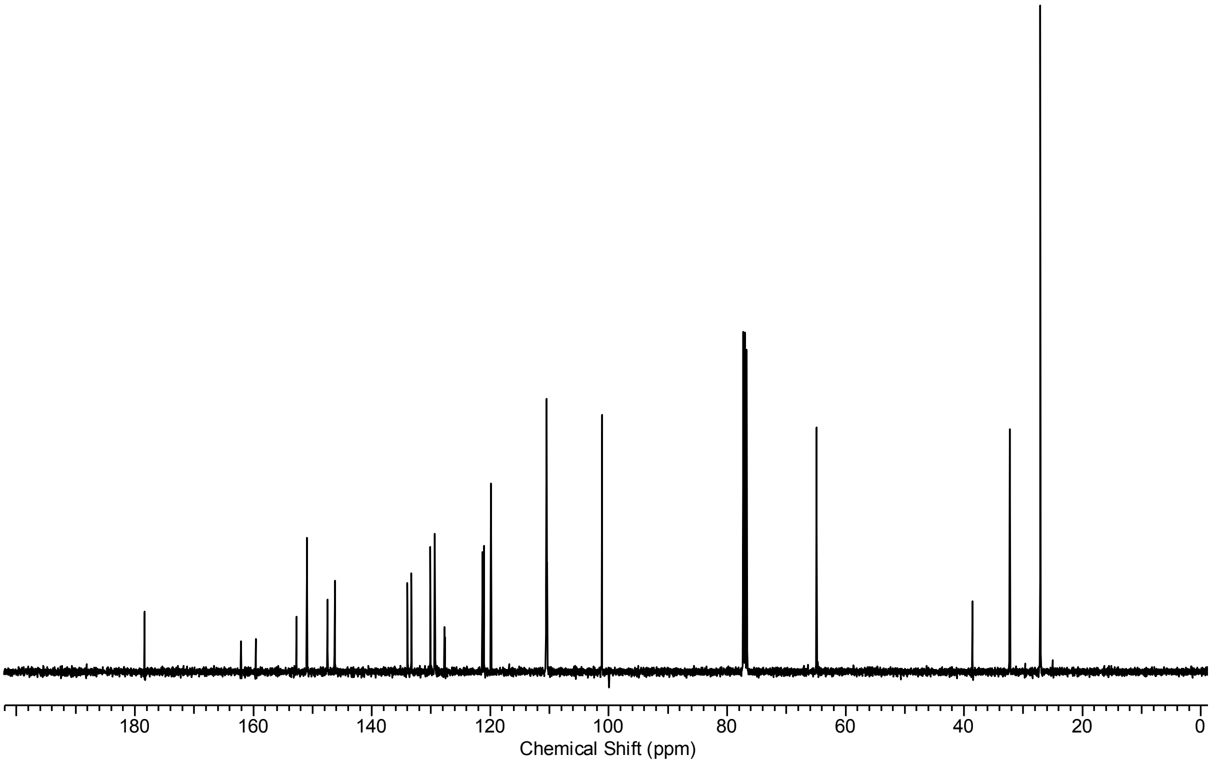

(S7) 2-(6-(7-Fluoroisoquinolin-3-yl)benzo[d][1,3]dioxol-5-yl)ethan-1-ol ($CDCl_3$)

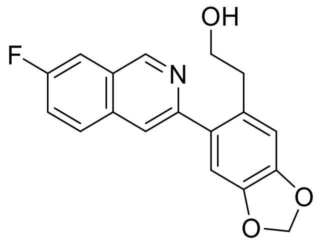

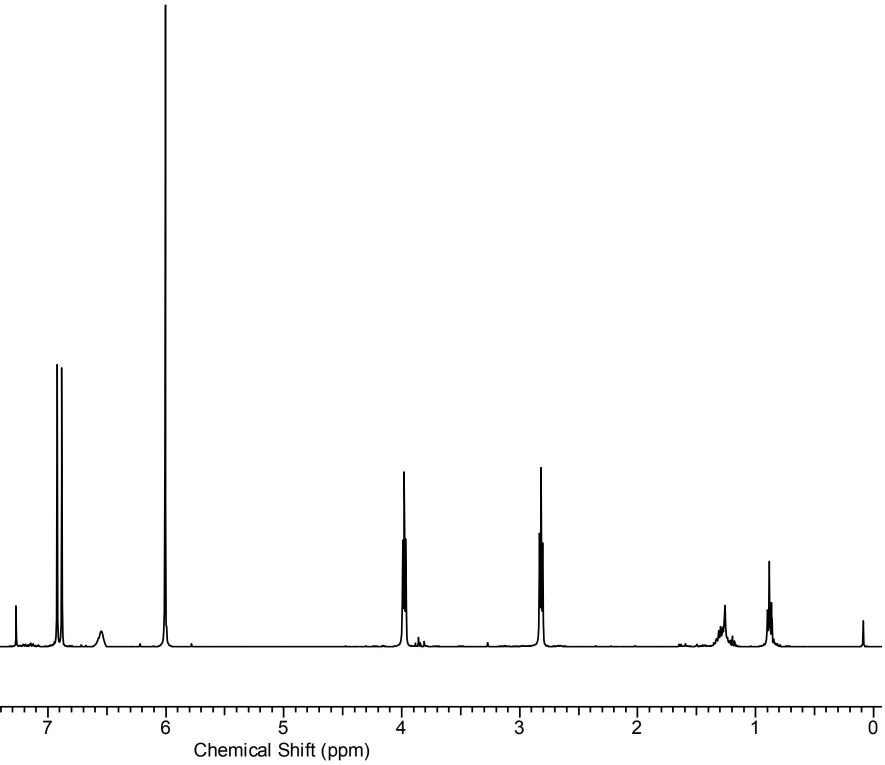

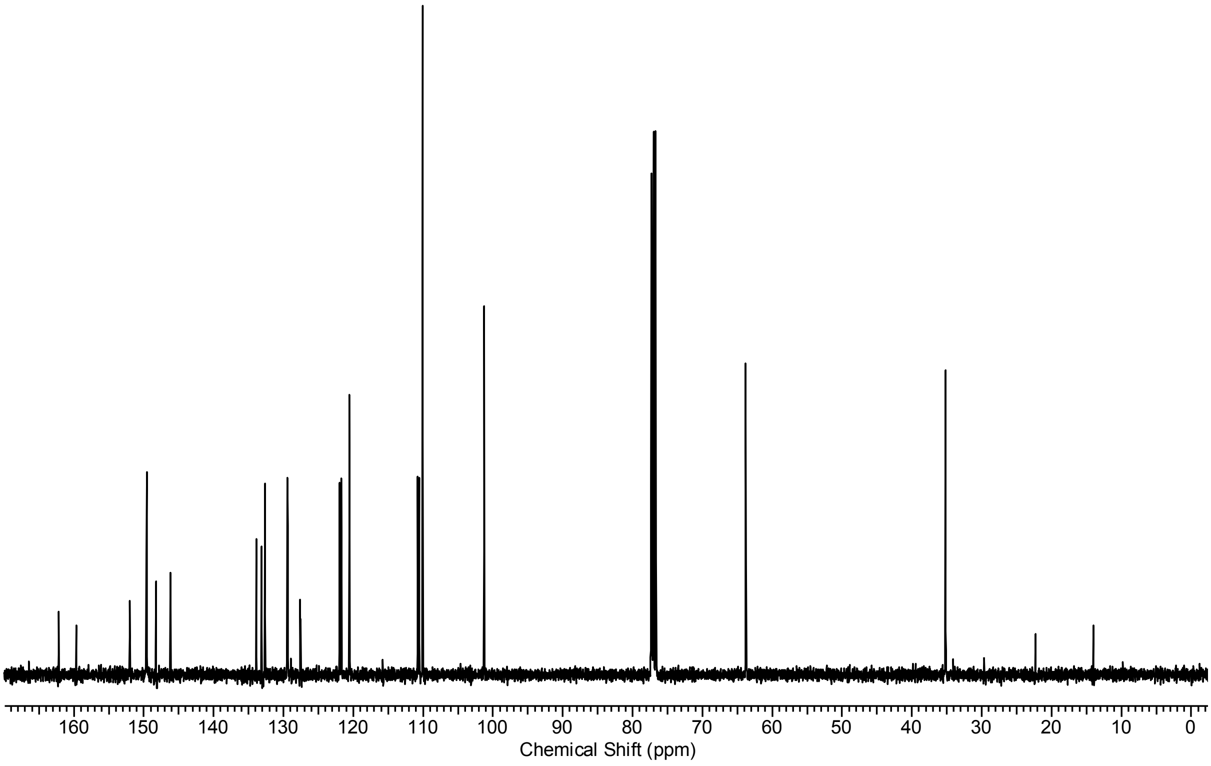

(16) 10-Fluoro-5,6-dihydro-[1,3]dioxolo[4,5-g]isoquinolino[3,2-a]isoquinolin-7-ium chloride ($DMSO-d_6$)

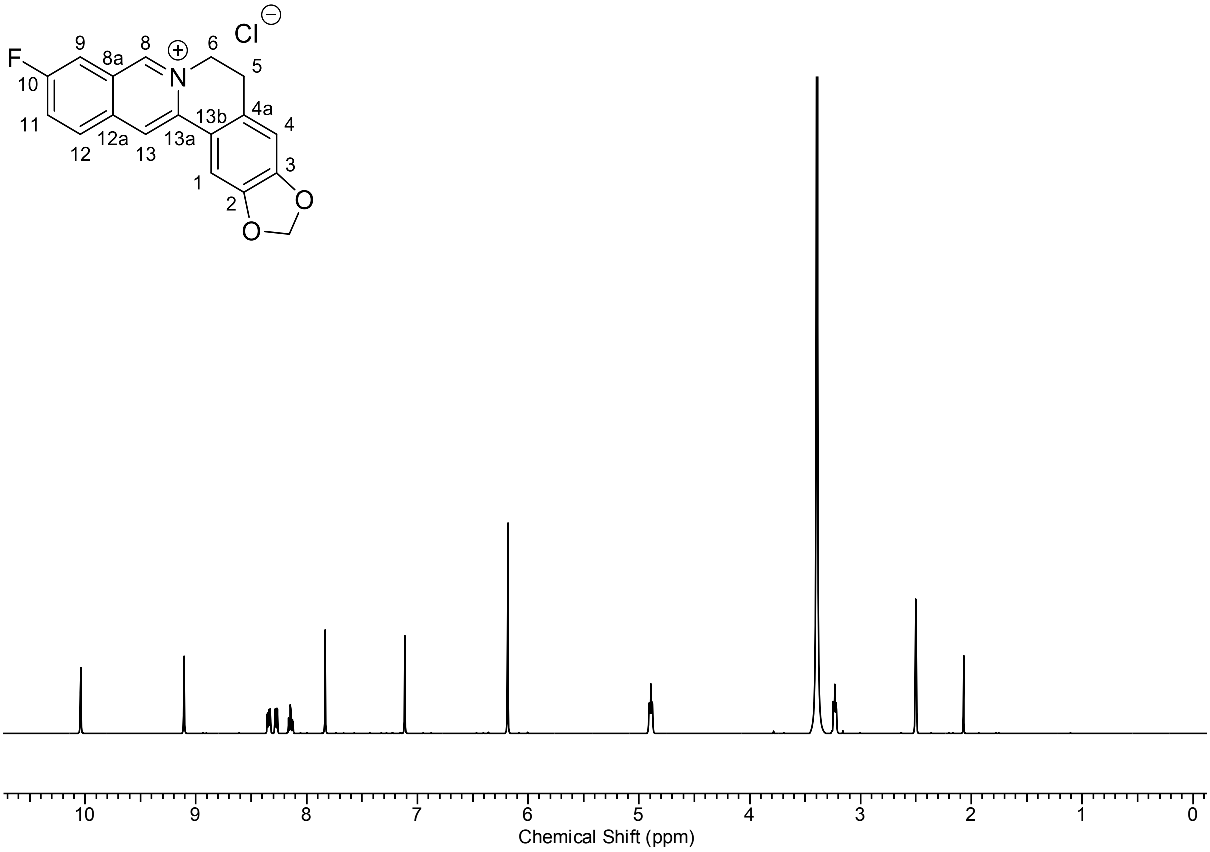

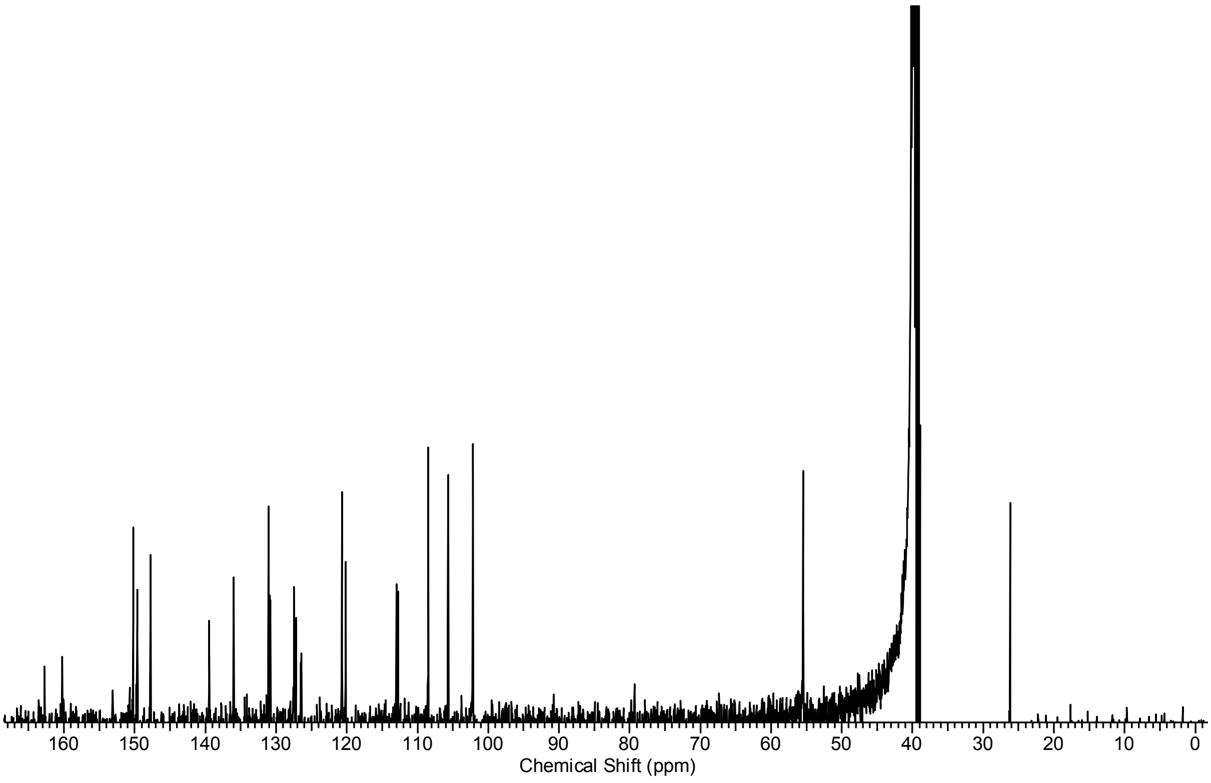

References

1. Geen, G. R.; Mann, I. S.; Mullane, M. Valerie; McKillop. A. *Tetrahedron* **1998**, *54*, 9875.

2. Blaskó, G.; Cordell, G. A.; Bhamarapravati, S.; Beecher, C. W. W. *Heterocycles* **1988**, *27*, 911.

3. Gao, C.-Y.; Lou, Z.-C.; Lin, F.-T.; Lin, M.-C.; Schiff, P. L. *Phytochemistry* **1987**, *26*, 3003.

4. Bałczewski, P.; Koprowski, M.; Bodzioch, A.; Marciniak, B.; Różycka-Sokołowska, E. *J. Org. Chem.* **2006**, *71*, 2899.

5. Moulis, C.; Gleye, J.; Stanislas, E. *Phytochemistry* **1977**, *16*, 1283.

6. McElhanon, J. R.; Shepodd, T. J. US 7449579 B1, Nov. 11, 2008.

7. Lenz, G. R. *J. Org. Chem.* **1977**, *42*, 1117.

8. Liu, H.; Xie, X.; Zhen, X.; Sun, H.; Li, J.; Zhu, L.; Li, Z.; Chen, Y.; Jiang, H.; Chen, K. US 2014/0088130 A

9. Moulis, C.; Stanislas, E.; Rossi, J.-C. *Org. Magn. Reson.* **1978**, *11*, 398.

10. Keawpradub, N.; Dej-adisai, S.; Yuenyongsawad, S. *Songklanakarin J. Sci. Technol.* **2005**, *27 (Suppl. 2)*, 455.

11. Hanaoka, M.; Yoshida, S.; Mukai, C. *Chem. Pharm. Bull.* **1989**, *37*, 3264.

12. Tong, S.; Yan, J.; Lou, J. *J. Liq. Chromatogr. Relat. Technol.* **2005**, *28*, 2979.

13. Hung, T. M.; Ngoc, T. M.; Youn, U. J.; Min, B. S.; Na, M.; Thuong, P. T.; Bae, K. *Biol. Pharm. Bull.* **2008**, *31*, 159.

14. Lee, J.-K.; Cho, J.-G.; Song, M.-C.; Yoo, J.-S.; Lee, D.-Y.; Yang, H.-J.; Han, K.-M.; Kim, D.-H.; Oh, Y.-J.; Jeong, T.-S.; Baek, N.-I. *J. Korean Soc. Appl. Biol. Chem.* **2009**, *52*, 646
